# Supplementary material for: Evaluation of central bank independence, macroprudential policy, and credit gap in developing countries
Source: PLoS One. 2023 May 16;18(5):e0285800. doi: 10.1371/journal.pone.0285800 (PMC10187913; doi:10.1371/journal.pone.0285800)
Supplement: S1 Data — (PDF) [file pone.0285800.s001.pdf]

Country	Period	cre_gap	mapp	cbi	inf	grow	ir	er
ALGERIA	2000Q1	0.0764	0.0833	0.3625	2.0878	3.3622	6.0000	73.4741
ALGERIA	2000Q2	-0.1512	0.0833	0.3625	-0.2508	3.5086	6.0000	73.9423
ALGERIA	2000Q3	-0.3911	0.0833	0.3625	-0.5326	3.6556	6.0000	77.9353
ALGERIA	2000Q4	-0.6403	0.0833	0.3625	0.0062	3.8197	6.0000	75.3428
ALGERIA	2001Q1	-0.5279	0.0833	0.3625	-0.1886	3.6187	6.0000	78.1552
ALGERIA	2001Q2	-0.4181	0.0833	0.3625	4.5644	3.4307	6.0000	80.1749
ALGERIA	2001Q3	-0.3071	0.0833	0.3625	5.4857	3.2346	6.0000	76.3439
ALGERIA	2001Q4	-0.1907	0.0833	0.3625	6.6922	3.0084	6.0000	77.8196
ALGERIA	2002Q1	0.4696	0.0833	0.3625	5.4903	3.5792	5.5000	80.2144
ALGERIA	2002Q2	1.1442	0.0833	0.3625	0.8539	4.1584	5.5000	79.9082
ALGERIA	2002Q3	1.8370	0.0833	0.3625	0.1744	4.8108	5.5000	79.9142
ALGERIA	2002Q4	2.5516	0.0833	0.3625	-0.8762	5.6093	5.5000	79.7234
ALGERIA	2003Q1	1.9996	0.0833	0.3625	-1.3344	5.6182	4.5000	79.6399
ALGERIA	2003Q2	1.4723	0.0833	0.3625	2.8604	5.8635	4.5000	78.3783
ALGERIA	2003Q3	0.9692	0.0833	0.3625	3.8941	6.3724	4.5000	77.0962
ALGERIA	2003Q4	0.4889	0.0833	0.3625	4.7661	7.2019	4.5000	72.6128
ALGERIA	2004Q1	0.2175	0.0833	0.3625	5.4448	6.1206	4.0000	71.4498
ALGERIA	2004Q2	-0.0355	0.0833	0.3625	3.7272	5.3317	4.0000	71.4880
ALGERIA	2004Q3	-0.2729	0.0833	0.3625	2.6530	4.7472	4.0000	72.5831
ALGERIA	2004Q4	-0.4969	0.0833	0.3625	2.2217	4.3016	4.0000	72.6137
ALGERIA	2005Q1	-0.4209	0.0833	0.3625	3.1672	4.4980	4.0000	72.9182
ALGERIA	2005Q2	-0.3361	0.0834	0.3625	3.6199	4.8056	4.0000	73.8386
ALGERIA	2005Q3	-0.2440	0.0835	0.3625	3.0857	5.2586	4.0000	73.6302
ALGERIA	2005Q4	-0.1462	0.0837	0.3625	0.7942	5.9078	4.0000	73.3799
ALGERIA	2006Q1	-0.2301	0.0843	0.3625	0.2222	4.7547	4.0000	73.9375
ALGERIA	2006Q2	-0.3109	0.0860	0.3625	1.7177	3.7414	4.0000	73.2834
ALGERIA	2006Q3	-0.3899	0.0910	0.3625	3.0893	2.7548	4.0000	72.1182
ALGERIA	2006Q4	-0.4681	0.1053	0.3625	4.1056	1.6845	4.0000	71.1582
ALGERIA	2007Q1	-0.3748	0.1460	0.3625	2.8733	2.2667	4.0000	70.8825
ALGERIA	2007Q2	-0.2818	0.1617	0.3625	2.0471	2.7107	4.0000	69.9916
ALGERIA	2007Q3	-0.1892	0.1716	0.3625	4.7459	3.0662	4.0000	67.6712
ALGERIA	2007Q4	-0.0972	0.1874	0.3625	4.7361	3.3729	4.0000	66.8299
ALGERIA	2008Q1	-0.2723	0.2280	0.3625	4.9453	3.1963	4.0000	65.3030
ALGERIA	2008Q2	-0.4475	0.2423	0.3625	5.5417	2.9855	4.0000	61.8851
ALGERIA	2008Q3	-0.6227	0.2473	0.3625	3.8803	2.7167	4.0000	60.7667
ALGERIA	2008Q4	-0.7970	0.2490	0.3625	4.6296	2.3601	4.0000	71.1826
ALGERIA	2009Q1	-0.0536	0.2497	0.3625	5.5882	2.3816	4.0000	73.1463
ALGERIA	2009Q2	0.6932	0.2499	0.3625	4.6503	2.2777	4.0000	73.0967
ALGERIA	2009Q3	1.4448	0.2500	0.3625	6.3201	2.0369	4.0000	72.5363
ALGERIA	2009Q4	2.2022	0.2500	0.3625	5.7374	1.6322	4.0000	72.7309
ALGERIA	2010Q1	1.8340	0.2500	0.4525	4.2263	2.2822	4.0000	73.7159
ALGERIA	2010Q2	1.4706	0.2500	0.4525	4.8113	2.7957	4.0000	75.3636
ALGERIA	2010Q3	1.1099	0.2500	0.4525	3.4009	3.2302	4.0000	74.7419
ALGERIA	2010Q4	0.7486	0.2500	0.4525	2.9462	3.6341	4.0000	74.9437
ALGERIA	2011Q1	0.2741	0.2500	0.4525	3.4360	3.4727	4.0000	72.1755
ALGERIA	2011Q2	-0.2094	0.2500	0.4525	3.9531	3.3079	4.0000	71.6635
ALGERIA	2011Q3	-0.7064	0.2500	0.4525	5.2182	3.1212	4.0000	73.9690
ALGERIA	2011Q4	-1.2214	0.2500	0.4525	5.0508	2.8919	4.0000	76.0563
ALGERIA	2012Q1	-1.3079	0.2500	0.4525	8.6234	3.0713	4.0000	74.0669
ALGERIA	2012Q2	-1.4196	0.2500	0.4525	9.1117	3.2024	4.0000	78.8582
ALGERIA	2012Q3	-1.5589	0.2499	0.4525	7.7274	3.3000	4.0000	79.3289
ALGERIA	2012Q4	-1.7273	0.2497	0.4525	8.6214	3.3748	4.0000	78.1025
ALGERIA	2013Q1	-1.3847	0.2491	0.4525	5.6849	3.2760	4.0000	79.0240
ALGERIA	2013Q2	-1.0712	0.2473	0.4525	3.5983	3.1519	4.0000	79.7345
ALGERIA	2013Q3	-0.7856	0.2424	0.4525	2.9411	2.9885	4.0000	81.4128
ALGERIA	2013Q4	-0.5257	0.2283	0.4525	0.6405	2.7676	4.0000	78.1524
ALGERIA	2014Q1	-0.4436	0.1883	0.4525	0.6956	3.0683	4.0000	78.4983
ALGERIA	2014Q2	-0.3818	0.1743	0.4525	1.7138	3.3204	4.0000	79.2669
ALGERIA	2014Q3	-0.3372	0.1693	0.4525	3.2745	3.5520	4.0000	82.7168
ALGERIA	2014Q4	-0.3063	0.1676	0.4525	5.7522	3.7891	4.0000	87.9039

ALGERIA	2015Q1	0.0908	0.1670	0.4525	5.1136	3.7438	4.0000	97.5474
ALGERIA	2015Q2	0.4815	0.1668	0.4525	5.2447	3.7253	4.0000	99.0178
ALGERIA	2015Q3	0.8696	0.1667	0.4525	4.6490	3.7316	4.0000	106.0524
ALGERIA	2015Q4	1.2585	0.1667	0.4525	3.7215	3.7635	4.0000	107.1317
ALGERIA	2016Q1	1.1021	0.1667	0.4525	4.7322	3.6375	4.0000	108.7168
ALGERIA	2016Q2	0.9516	0.1667	0.4525	6.9548	3.5264	4.0000	110.3718
ALGERIA	2016Q3	0.8083	0.1667	0.4525	6.7418	3.4180	4.0000	109.7371
ALGERIA	2016Q4	0.6730	0.1667	0.4525	6.3399	3.3000	4.0000	110.5274
ALGERIA	2017Q1	0.6368	0.1667	0.4525	7.2768	3.0207	3.5000	110.0140
ALGERIA	2017Q2	0.6089	0.1667	0.4525	5.0519	2.6874	3.5000	107.8459
ALGERIA	2017Q3	0.5887	0.1667	0.4525	4.1932	2.2630	3.5000	113.2077
ALGERIA	2017Q4	0.5754	0.1667	0.4525	5.2850	1.7000	3.5000	114.9327
ALGERIA	2018Q1	0.2927	0.1675	0.4525	5.2608	1.2750	0.9375	112.3782
ALGERIA	2018Q2	0.0140	0.1680	0.4525	4.9306	1.2500	0.9375	113.7834
ALGERIA	2018Q3	-0.2625	0.1689	0.4525	4.6003	1.2250	0.9375	115.1886
ALGERIA	2018Q4	-0.5386	0.1696	0.4525	4.2700	1.2000	0.9375	116.5938
ALGERIA	2019Q1	-0.6554	0.1705	0.4525	3.6904	1.1000	0.9375	117.2837
ALGERIA	2019Q2	-0.7748	0.1715	0.4525	3.1109	1.0000	0.9375	117.9737
ALGERIA	2019Q3	-0.8976	0.1726	0.4525	2.5313	0.9000	0.9375	118.6636
ALGERIA	2019Q4	-1.0242	0.1737	0.4525	1.9518	0.8000	0.9375	119.3536
ALGERIA	2020Q1	-0.4850	0.1747	0.4525	2.0676	-0.7702	0.9375	121.2094
ALGERIA	2020Q2	0.0513	0.1758	0.4525	2.1834	-2.3405	0.9375	123.0652
ALGERIA	2020Q3	0.5858	0.1769	0.4525	2.2993	-3.9107	0.9375	124.9210
ALGERIA	2020Q4	1.1197	0.1781	0.4525	2.4151	-5.4810	0.9375	126.7768
ALGERIA	2021Q1	-0.0882	0.2253	0.4525	5.9000	1.3000	3.0000	128.6100
ALGERIA	2021Q2	-0.0915	0.2268	0.4525	5.4000	6.5000	3.0000	134.9600
ALGERIA	2021Q3	-0.0949	0.2283	0.4525	9.6000	2.9000	3.0000	135.3100
ALGERIA	2021Q4	-0.0983	0.2297	0.4525	8.5000	3.2000	3.0000	135.4200
ARGENTINA	2000Q1	2.2344	0.2513	0.8026	-1.1752	-0.1768	8.2500	0.9995
ARGENTINA	2000Q2	2.2816	0.2528	0.8026	-0.7221	-0.3971	8.2500	0.9995
ARGENTINA	2000Q3	2.3953	0.2576	0.8026	-0.6310	-0.6122	8.2500	0.9995
ARGENTINA	2000Q4	2.5731	0.2717	0.8026	-0.9000	-1.9306	8.2500	0.9995
ARGENTINA	2001Q1	2.2953	0.3117	0.8026	0.7920	-2.0245	25.0000	0.9995
ARGENTINA	2001Q2	2.0719	0.3258	0.8026	2.1543	-0.1679	25.0000	0.9995
ARGENTINA	2001Q3	1.8960	0.3308	0.8026	3.2161	-4.9287	25.0000	0.9995
ARGENTINA	2001Q4	1.7593	0.3327	0.8026	4.0000	-10.5098	25.0000	0.9995
ARGENTINA	2002Q1	1.0422	0.3339	0.8026	13.1532	-16.3391	41.2500	2.9500
ARGENTINA	2002Q2	0.3441	0.3359	0.8026	22.2410	-13.5118	41.2500	3.7500
ARGENTINA	2002Q3	-0.3462	0.3409	0.8026	31.4576	-9.7709	41.2500	3.6900
ARGENTINA	2002Q4	-1.0400	0.3550	0.8026	41.0000	-3.4171	41.2500	3.3200
ARGENTINA	2003Q1	-1.5156	0.3950	0.7903	33.6869	5.4171	3.7500	2.9300
ARGENTINA	2003Q2	-2.0166	0.4091	0.7903	26.7473	7.7490	3.7500	2.7500
ARGENTINA	2003Q3	-2.5526	0.4140	0.7903	20.0329	10.1585	3.7500	2.8650
ARGENTINA	2003Q4	-3.1322	0.4157	0.7903	13.4000	11.7324	3.7500	2.9050
ARGENTINA	2004Q1	-2.8898	0.4163	0.7903	11.7559	11.2574	2.0000	2.8400
ARGENTINA	2004Q2	-2.7024	0.4166	0.7903	10.0164	7.0740	2.0000	2.9380
ARGENTINA	2004Q3	-2.5732	0.4166	0.7903	8.1445	1.6351	2.0000	2.9610
ARGENTINA	2004Q4	-2.5033	0.4167	0.7903	6.1000	-0.3262	2.0000	2.9590
ARGENTINA	2005Q1	-1.9799	0.4167	0.7903	7.2432	7.2188	4.2500	2.8970
ARGENTINA	2005Q2	-1.5138	0.4167	0.7903	8.1947	13.0780	4.2500	2.8670
ARGENTINA	2005Q3	-1.1020	0.4167	0.7903	8.9747	6.9720	4.2500	2.8900
ARGENTINA	2005Q4	-0.7404	0.4167	0.7903	9.6000	7.7829	4.2500	3.0120
ARGENTINA	2006Q1	-0.3529	0.4167	0.7903	9.8522	7.8496	7.2500	3.0620
ARGENTINA	2006Q2	-0.0057	0.4167	0.7903	9.9685	5.5716	7.2500	3.0660
ARGENTINA	2006Q3	0.3066	0.4167	0.7903	9.9512	9.3805	7.2500	3.0840
ARGENTINA	2006Q4	0.5892	0.4167	0.7903	9.8000	9.6770	7.2500	3.0420
ARGENTINA	2007Q1	0.8304	0.4167	0.7903	9.7686	8.3589	8.7500	3.0800
ARGENTINA	2007Q2	1.0521	0.4167	0.7903	9.5993	9.8594	8.7500	3.0730
ARGENTINA	2007Q3	1.2586	0.4167	0.7903	9.2887	8.4278	8.7500	3.1300
ARGENTINA	2007Q4	1.4535	0.4167	0.7903	8.8300	9.2673	8.7500	3.1290
ARGENTINA	2008Q1	1.1466	0.4167	0.7903	9.0041	6.9123	10.0000	3.1480

ARGENTINA	2008Q2	0.8330	0.4167	0.7903	9.0241	5.4527	10.0000	3.0050
ARGENTINA	2008Q3	0.5139	0.4167	0.7903	8.8903	6.0060	10.0000	3.1150
ARGENTINA	2008Q4	0.1901	0.4167	0.7903	8.6000	-1.9790	10.0000	3.4330
ARGENTINA	2009Q1	0.0837	0.4167	0.7903	8.6223	-6.1888	10.2500	3.7000
ARGENTINA	2009Q2	-0.0267	0.4167	0.7903	8.4823	-11.2745	10.2500	3.7770
ARGENTINA	2009Q3	-0.1409	0.4167	0.7903	8.1771	-5.6512	10.2500	3.8230
ARGENTINA	2009Q4	-0.2586	0.4167	0.7903	7.7000	0.2820	10.2500	3.7800
ARGENTINA	2010Q1	-0.3366	0.4167	0.7903	11.3551	5.7133	9.0000	3.8580
ARGENTINA	2010Q2	-0.4173	0.4167	0.7903	14.9062	16.2442	9.0000	3.9110
ARGENTINA	2010Q3	-0.5000	0.4167	0.7903	18.4295	9.5077	9.0000	3.9400
ARGENTINA	2010Q4	-0.5836	0.4167	0.7903	22.0000	8.6098	9.0000	3.9560
ARGENTINA	2011Q1	-0.4058	0.4167	0.7903	22.0818	8.2926	10.0000	4.0340
ARGENTINA	2011Q2	-0.2261	0.4167	0.7903	22.2889	4.4433	10.0000	4.0900
ARGENTINA	2011Q3	-0.0425	0.4167	0.7903	22.6260	6.4094	10.0000	4.1850
ARGENTINA	2011Q4	0.1469	0.4167	0.7903	23.1000	5.2171	10.0000	4.2840
ARGENTINA	2012Q1	0.3157	0.4167	0.7602	23.4086	1.5642	9.7500	4.3590
ARGENTINA	2012Q2	0.4944	0.4167	0.7602	23.8709	-4.6318	9.7500	4.5250
ARGENTINA	2012Q3	0.6849	0.4167	0.7602	24.4970	-1.1183	9.7500	4.6770
ARGENTINA	2012Q4	0.8886	0.4167	0.7602	25.3000	0.5558	9.7500	4.8980
ARGENTINA	2013Q1	0.9345	0.4167	0.7602	23.9515	0.6540	13.0000	5.1020
ARGENTINA	2013Q2	0.9952	0.4167	0.7602	22.7683	6.2460	13.0000	5.3750
ARGENTINA	2013Q3	1.0705	0.4167	0.7602	21.7253	2.5584	13.0000	5.7750
ARGENTINA	2013Q4	1.1598	0.4167	0.7602	20.8000	-0.0510	13.0000	6.5010
ARGENTINA	2014Q1	0.6562	0.4167	0.7602	22.7451	-0.8890	18.0000	7.9520
ARGENTINA	2014Q2	0.1630	0.4167	0.7602	24.8297	-2.0489	18.0000	8.0830
ARGENTINA	2014Q3	-0.3222	0.4167	0.7602	27.0985	-4.2385	18.0000	8.3800
ARGENTINA	2014Q4	-0.8022	0.4167	0.7602	29.6000	-2.8157	18.0000	8.5100
ARGENTINA	2015Q1	-0.6553	0.4167	0.7602	29.8971	0.0266	22.0000	8.7700
ARGENTINA	2015Q2	-0.5075	0.4167	0.7602	30.4866	3.9070	22.0000	9.0400
ARGENTINA	2015Q3	-0.3602	0.4167	0.7602	31.3812	3.8065	22.0000	9.3750
ARGENTINA	2015Q4	-0.2147	0.4167	0.7602	32.6000	2.6468	22.0000	13.1000
ARGENTINA	2016Q1	-0.4061	0.4167	0.7602	34.5118	0.5752	29.0000	14.6000
ARGENTINA	2016Q2	-0.6011	0.4167	0.7602	36.8146	-3.6631	29.0000	15.0000
ARGENTINA	2016Q3	-0.8002	0.4167	0.7602	39.5578	-3.6663	29.0000	15.3000
ARGENTINA	2016Q4	-1.0036	0.4167	0.7602	42.8000	-1.8960	29.0000	15.9000
ARGENTINA	2017Q1	-0.4517	0.4167	0.7602	38.2060	0.3319	30.0000	15.4000
ARGENTINA	2017Q2	0.0972	0.4167	0.7602	34.0821	0.7998	30.0000	16.6000
ARGENTINA	2017Q3	0.6441	0.4167	0.7602	30.3401	1.8300	30.0000	17.3000
ARGENTINA	2017Q4	1.1903	0.4167	0.7602	26.9000	2.8639	30.0000	18.6000
ARGENTINA	2018Q1	-0.1425	0.4153	0.7602	29.5078	-2.4460	14.8130	19.4458
ARGENTINA	2018Q2	-0.1466	0.4125	0.7602	33.0093	-2.3267	14.8130	22.3288
ARGENTINA	2018Q3	-0.1507	0.4093	0.7602	36.5107	-2.2073	14.8130	25.2119
ARGENTINA	2018Q4	-0.1548	0.4065	0.7602	40.0122	-2.0880	14.8130	28.0950
ARGENTINA	2019Q1	-0.1589	0.4043	0.7602	42.6648	-4.0423	13.7500	33.1082
ARGENTINA	2019Q2	-0.1631	0.4026	0.7602	45.3174	-5.9966	13.7500	38.1214
ARGENTINA	2019Q3	-0.1672	0.4006	0.7602	47.9700	-7.9509	13.7500	43.1347
ARGENTINA	2019Q4	-0.1713	0.3978	0.7602	50.6226	-9.9052	13.7500	48.1479
ARGENTINA	2020Q1	-0.1754	0.3941	0.7602	47.9271	-5.6289	9.5000	53.7457
ARGENTINA	2020Q2	-0.1795	0.3898	0.7602	45.2317	-1.3526	9.5000	59.3435
ARGENTINA	2020Q3	-0.1836	0.3856	0.7602	42.5363	2.9237	9.5000	64.9413
ARGENTINA	2020Q4	-0.1878	0.3819	0.7602	39.8409	7.2000	9.5000	70.5392
ARGENTINA	2021Q1	-0.1919	0.4342	0.7602	40.6471	9.2466	38.0000	90.7450
ARGENTINA	2021Q2	-0.1960	0.4350	0.7602	48.4656	10.3969	38.0000	94.3510
ARGENTINA	2021Q3	-0.2001	0.4359	0.7602	51.8956	10.9732	38.0000	98.5580
ARGENTINA	2021Q4	-0.2042	0.4367	0.7602	51.3895	10.9753	38.0000	102.1600
CHINA	2000Q1	2.3875	0.0833	0.6021	-0.4316	7.8688	0.8100	8.2783
CHINA	2000Q2	1.5699	0.0833	0.6021	0.3999	8.0759	0.8100	8.2784
CHINA	2000Q3	0.8041	0.0833	0.6021	1.2313	8.2830	0.8100	8.2784
CHINA	2000Q4	0.0909	0.0833	0.6021	2.0628	8.4901	0.8100	8.2785
CHINA	2001Q1	-1.0238	0.0833	0.6021	2.0589	8.4515	0.8100	8.2781
CHINA	2001Q2	-2.0852	0.0834	0.6021	2.0549	8.4129	0.8100	8.2778

CHINA	2001Q3	-3.0925	0.0835	0.6021	2.0510	8.3743	0.8100	8.2774
CHINA	2001Q4	-4.0435	0.0837	0.6021	2.0470	8.3357	0.8100	8.2771
CHINA	2002Q1	-2.8009	0.0843	0.6021	1.6858	8.5352	0.6750	8.2770
CHINA	2002Q2	-1.4910	0.0861	0.6021	1.3246	8.7347	0.6750	8.2770
CHINA	2002Q3	-0.1056	0.0912	0.6021	0.9633	8.9342	0.6750	8.2770
CHINA	2002Q4	1.3648	0.1056	0.6021	0.6021	9.1336	0.6750	8.2770
CHINA	2003Q1	3.1092	0.1469	0.6021	1.1024	9.3597	0.6750	8.2770
CHINA	2003Q2	4.9566	0.1644	0.6021	1.6026	9.5858	0.6750	8.2770
CHINA	2003Q3	6.9136	0.1792	0.6021	2.1029	9.8119	0.6750	8.2770
CHINA	2003Q4	8.9837	0.2090	0.6021	2.6032	10.0380	0.6750	8.2770
CHINA	2004Q1	7.3641	0.2897	0.6021	3.6904	10.0569	0.8325	8.2770
CHINA	2004Q2	5.8504	0.3180	0.6021	4.7776	10.0758	0.8325	8.2769
CHINA	2004Q3	4.4316	0.3280	0.6021	5.8648	10.0947	0.8325	8.2769
CHINA	2004Q4	3.0931	0.3314	0.6021	6.9520	10.1136	0.8325	8.2768
CHINA	2005Q1	1.8779	0.3327	0.6016	6.1899	10.4339	0.8325	8.2562
CHINA	2005Q2	0.7062	0.3331	0.6010	5.4279	10.7541	0.8325	8.2356
CHINA	2005Q3	-0.4426	0.3333	0.6004	4.6658	11.0743	0.8325	8.2149
CHINA	2005Q4	-1.5895	0.3333	0.5999	3.9037	11.3946	0.8325	8.1943
CHINA	2006Q1	-1.7281	0.3333	0.5999	3.9094	11.7262	0.8325	8.1391
CHINA	2006Q2	-1.9051	0.3333	0.5999	3.9151	12.0578	0.8325	8.0839
CHINA	2006Q3	-2.1393	0.3333	0.5999	3.9208	12.3894	0.8325	8.0287
CHINA	2006Q4	-2.4481	0.3333	0.5999	3.9265	12.7210	0.8325	7.9734
CHINA	2007Q1	-3.0191	0.3333	0.5999	4.8823	13.0984	0.8325	7.8820
CHINA	2007Q2	-3.6954	0.3333	0.5999	5.8381	13.4759	0.8325	7.7905
CHINA	2007Q3	-4.4896	0.3333	0.5999	6.7939	13.8534	0.8325	7.6990
CHINA	2007Q4	-5.4121	0.3333	0.5999	7.7497	14.2309	0.8325	7.6075
CHINA	2008Q1	-6.5738	0.3333	0.5999	7.7611	13.0858	0.6975	7.4428
CHINA	2008Q2	-7.8756	0.3333	0.5999	7.7725	11.9408	0.6975	7.2781
CHINA	2008Q3	-9.3176	0.3333	0.5999	7.7839	10.7957	0.6975	7.1134
CHINA	2008Q4	-10.8948	0.3333	0.5999	7.7953	9.6507	0.6975	6.9487
CHINA	2009Q1	-6.0497	0.3333	0.5999	5.7941	9.5877	0.6975	6.9193
CHINA	2009Q2	-1.3117	0.3333	0.5999	3.7929	9.5247	0.6975	6.8900
CHINA	2009Q3	3.3404	0.3333	0.5999	1.7917	9.4617	0.6975	6.8607
CHINA	2009Q4	7.9285	0.3333	0.5999	-0.2095	9.3987	0.6975	6.8314
CHINA	2010Q1	7.4146	0.3333	0.5999	1.5632	9.7080	0.8125	6.8161
CHINA	2010Q2	6.8719	0.3334	0.5999	3.3359	10.0173	0.8125	6.8008
CHINA	2010Q3	6.3107	0.3335	0.5999	5.1087	10.3266	0.8125	6.7856
CHINA	2010Q4	5.7371	0.3337	0.5999	6.8814	10.6359	0.8125	6.7703
CHINA	2011Q1	3.7397	0.3343	0.5999	7.1800	10.3646	0.8125	6.6931
CHINA	2011Q2	1.7306	0.3361	0.5999	7.4785	10.0934	0.8125	6.6159
CHINA	2011Q3	-0.2939	0.3412	0.5999	7.7771	9.8221	0.8125	6.5387
CHINA	2011Q4	-2.3385	0.3556	0.5999	8.0757	9.5508	0.8125	6.4615
CHINA	2012Q1	-2.0805	0.3969	0.5999	6.6396	9.1291	0.8125	6.4242
CHINA	2012Q2	-1.8506	0.4144	0.5999	5.2035	8.7073	0.8125	6.3869
CHINA	2012Q3	-1.6506	0.4293	0.5999	3.7673	8.2855	0.8125	6.3496
CHINA	2012Q4	-1.4814	0.4594	0.5999	2.3312	7.8637	0.8125	6.3123
CHINA	2013Q1	-1.4501	0.5407	0.5999	2.2893	7.8393	0.8125	6.2832
CHINA	2013Q2	-1.4480	0.5708	0.5999	2.2473	7.8149	0.8125	6.2540
CHINA	2013Q3	-1.4731	0.5858	0.5999	2.2053	7.7905	0.8125	6.2249
CHINA	2013Q4	-1.5222	0.6038	0.5999	2.1634	7.7662	0.8125	6.1958
CHINA	2014Q1	-1.4613	0.6462	0.5999	1.8803	7.6811	0.8125	6.1827
CHINA	2014Q2	-1.4155	0.6642	0.5999	1.5972	7.5960	0.8125	6.1696
CHINA	2014Q3	-1.3792	0.6791	0.5999	1.3141	7.5109	0.8125	6.1565
CHINA	2014Q4	-1.3456	0.7090	0.5999	1.0311	7.4258	0.8125	6.1434
CHINA	2015Q1	0.3028	0.7897	0.5999	0.7726	7.3297	0.7250	6.1644
CHINA	2015Q2	1.9644	0.8180	0.5999	0.5141	7.2335	0.7250	6.1855
CHINA	2015Q3	3.6475	0.8280	0.5999	0.2556	7.1374	0.7250	6.2065
CHINA	2015Q4	5.3589	0.8314	0.5999	-0.0029	7.0413	0.7250	6.2275
CHINA	2016Q1	4.9230	0.8327	0.5999	0.3496	6.9932	0.5625	6.3317
CHINA	2016Q2	4.5215	0.8331	0.5999	0.7022	6.9450	0.5625	6.4360
CHINA	2016Q3	4.1527	0.8333	0.5999	1.0548	6.8969	0.5625	6.5402

CHINA	2016Q4	3.8121	0.8333	0.5999	1.4073	6.8488	0.5625	6.6445
CHINA	2017Q1	2.2500	0.8333	0.5999	2.1137	6.8734	0.6250	6.6730
CHINA	2017Q2	0.6994	0.8333	0.5999	2.8200	6.8980	0.6250	6.7016
CHINA	2017Q3	-0.8506	0.8333	0.5999	3.5263	6.9226	0.6250	6.7302
CHINA	2017Q4	-2.4115	0.8333	0.5999	4.2327	6.9472	0.6250	6.7588
CHINA	2018Q1	-2.9309	0.8333	0.5999	4.0494	6.8978	0.6375	6.7231
CHINA	2018Q2	-3.4814	0.8333	0.5999	3.8662	6.8485	0.6375	6.6874
CHINA	2018Q3	-4.0703	0.8333	0.5999	3.6830	6.7991	0.6375	6.6517
CHINA	2018Q4	-4.7030	0.8333	0.5999	3.4997	6.7498	0.6375	6.6160
CHINA	2019Q1	-4.2170	0.8404	0.5999	2.9467	6.5498	0.6250	6.6891
CHINA	2019Q2	-3.7774	0.8512	0.5999	2.3936	6.3497	0.6250	6.7622
CHINA	2019Q3	-3.3814	0.8620	0.5999	1.8405	6.1497	0.6250	6.8353
CHINA	2019Q4	-3.0239	0.8728	0.5999	1.2875	5.9497	0.6250	6.9084
CHINA	2020Q1	-0.3317	0.8836	0.5999	1.1336	5.0373	0.5500	6.9065
CHINA	2020Q2	2.3386	0.8944	0.5999	0.9797	4.1249	0.5500	6.9046
CHINA	2020Q3	4.9961	0.9053	0.5999	0.8258	3.2124	0.5500	6.9027
CHINA	2020Q4	7.6489	0.9161	0.5999	0.6719	2.3000	0.5500	6.9008
CHINA	2021Q1	-0.1947	0.9300	0.5999	0.9810	9.5183	-2.2529	6.4490
CHINA	2021Q2	-0.2008	0.9408	0.5999	0.9810	8.7262	-0.1561	6.4490
CHINA	2021Q3	-0.2069	0.9517	0.5999	0.9810	7.7139	1.0274	6.4490
CHINA	2021Q4	-0.2129	0.9625	0.5999	0.9810	6.4816	1.2976	6.4490
BELARUS	2000Q1	4.7691	0.0833	0.3729	118.5881	4.0030	80.0000	0.0435
BELARUS	2000Q2	4.8684	0.0833	0.3729	108.4588	4.5962	80.0000	0.0675
BELARUS	2000Q3	4.9454	0.0833	0.3729	101.3670	5.1913	80.0000	0.1033
BELARUS	2000Q4	4.9937	0.0833	0.3729	80.4308	5.8000	80.0000	0.1180
BELARUS	2001Q1	3.0135	0.0833	0.3729	60.3610	5.5070	66.0000	0.1293
BELARUS	2001Q2	0.9780	0.0833	0.3729	53.2485	5.2337	66.0000	0.1380
BELARUS	2001Q3	-1.1319	0.0833	0.3729	43.7062	4.9749	66.0000	0.1477
BELARUS	2001Q4	-3.3361	0.0833	0.3729	37.9443	4.7253	66.0000	0.1580
BELARUS	2002Q1	-3.3096	0.0833	0.3729	38.5224	4.7951	38.0000	0.1707
BELARUS	2002Q2	-3.4083	0.0833	0.3729	36.7074	4.8707	38.0000	0.1800
BELARUS	2002Q3	-3.6421	0.0833	0.3729	35.6082	4.9535	38.0000	0.1865
BELARUS	2002Q4	-4.0187	0.0833	0.3729	31.6802	5.0453	38.0000	0.1920
BELARUS	2003Q1	-4.3834	0.0833	0.3729	26.7413	5.5056	28.0000	0.1996
BELARUS	2003Q2	-4.8992	0.0833	0.3729	24.8041	5.9856	28.0000	0.2060
BELARUS	2003Q3	-5.5666	0.0833	0.3729	24.8612	6.4948	28.0000	0.2108
BELARUS	2003Q4	-6.3825	0.0833	0.3729	23.8141	7.0432	28.0000	0.2156
BELARUS	2004Q1	-3.7361	0.0833	0.3729	20.0908	8.0202	17.0000	0.2150
BELARUS	2004Q2	-1.2137	0.0833	0.3729	17.8052	9.0663	17.0000	0.2155
BELARUS	2004Q3	1.2052	0.0833	0.3729	15.5512	10.2021	17.0000	0.2167
BELARUS	2004Q4	3.5421	0.0833	0.3729	13.5302	11.4497	17.0000	0.2170
BELARUS	2005Q1	2.8061	0.0833	0.3729	11.6665	10.7634	11.0000	0.2153
BELARUS	2005Q2	2.0200	0.0833	0.3729	10.1707	10.2000	11.0000	0.2150
BELARUS	2005Q3	1.1934	0.0833	0.3729	9.4162	9.7485	11.0000	0.2150
BELARUS	2005Q4	0.3346	0.0833	0.3729	8.2499	9.4000	11.0000	0.2152
BELARUS	2006Q1	0.0665	0.0833	0.3729	7.4299	9.4032	10.0000	0.2149
BELARUS	2006Q2	-0.2173	0.0833	0.3729	6.8198	9.5026	10.0000	0.2142
BELARUS	2006Q3	-0.5082	0.0833	0.3729	6.2547	9.7003	10.0000	0.2141
BELARUS	2006Q4	-0.7973	0.0833	0.3729	6.5678	10.0000	10.0000	0.2140
BELARUS	2007Q1	-1.0502	0.0833	0.3729	7.4069	9.5097	10.0000	0.2143
BELARUS	2007Q2	-1.2824	0.0833	0.3729	6.8337	9.1178	10.0000	0.2145
BELARUS	2007Q3	-1.4834	0.0833	0.3729	7.8019	8.8166	10.0000	0.2149
BELARUS	2007Q4	-0.4972	0.0833	0.3729	10.2235	8.6000	10.0000	0.2150
BELARUS	2008Q1	-0.7187	0.0833	0.3729	12.1900	8.8642	12.0000	0.2145
BELARUS	2008Q2	-0.8897	0.0833	0.3729	14.4271	9.2140	12.0000	0.2125
BELARUS	2008Q3	-0.9996	0.0833	0.3729	15.0885	9.6564	12.0000	0.2111
BELARUS	2008Q4	-1.0373	0.0833	0.3729	13.5965	10.2000	12.0000	0.2200
BELARUS	2009Q1	-0.1233	0.0833	0.3729	14.3313	7.6558	13.5000	0.2837
BELARUS	2009Q2	0.8869	0.0834	0.3729	12.9708	5.1734	13.5000	0.2837
BELARUS	2009Q3	2.0057	0.0834	0.3729	11.6666	2.7044	13.5000	0.2764
BELARUS	2009Q4	3.2452	0.0837	0.3729	9.8646	0.2000	13.5000	0.2863

BELARUS	2010Q1	4.5093	0.0843	0.7487	6.0734	2.1312	10.5000	0.2978
BELARUS	2010Q2	5.9133	0.0860	0.7487	6.6242	4.0160	10.5000	0.3010
BELARUS	2010Q3	7.4629	0.0909	0.7487	7.5038	5.8912	10.5000	0.3010
BELARUS	2010Q4	9.1601	0.1050	0.7487	9.5055	7.7937	10.5000	0.3000
BELARUS	2011Q1	8.0702	0.1450	0.7487	11.7724	7.1639	45.0000	0.3045
BELARUS	2011Q2	7.1171	0.1591	0.7487	27.4287	6.5864	45.0000	0.4964
BELARUS	2011Q3	6.2875	0.1640	0.7487	48.8696	6.0499	45.0000	0.5599
BELARUS	2011Q4	5.5634	0.1657	0.7487	70.4942	5.5437	45.0000	0.8350
BELARUS	2012Q1	2.0912	0.1663	0.7487	73.1613	4.6060	30.0000	0.8020
BELARUS	2012Q2	-1.3223	0.1666	0.7487	60.1617	3.6703	30.0000	0.8320
BELARUS	2012Q3	-4.7036	0.1666	0.7487	42.1305	2.7183	30.0000	0.8440
BELARUS	2012Q4	-8.0784	0.1667	0.7487	22.2509	1.7314	30.0000	0.8570
BELARUS	2013Q1	-7.1371	0.1667	0.7487	20.4350	1.6419	23.5000	0.8670
BELARUS	2013Q2	-6.2297	0.1667	0.7487	17.7600	1.4962	23.5000	0.8790
BELARUS	2013Q3	-5.3694	0.1667	0.7487	14.8131	1.2916	23.5000	0.9080
BELARUS	2013Q4	-4.5657	0.1667	0.7487	14.6424	1.0240	23.5000	0.9510
BELARUS	2014Q1	-3.9350	0.1667	0.7487	14.4404	1.2896	20.0000	0.9870
BELARUS	2014Q2	-3.3699	0.1667	0.7487	17.1746	1.4922	20.0000	1.0200
BELARUS	2014Q3	-2.8711	0.1668	0.7487	18.3761	1.6358	20.0000	1.0580
BELARUS	2014Q4	-2.4372	0.1670	0.7487	16.5065	1.7231	20.0000	1.1850
BELARUS	2015Q1	-1.1790	0.1676	0.7487	15.4155	0.4798	25.0000	1.4740
BELARUS	2015Q2	0.0223	0.1693	0.7487	13.1893	-0.8424	25.0000	1.5346
BELARUS	2015Q3	1.1723	0.1743	0.7487	11.4118	-2.2696	25.0000	1.7703
BELARUS	2015Q4	2.2763	0.1883	0.7487	10.9793	-3.8296	25.0000	1.8569
BELARUS	2016Q1	1.7351	0.2283	0.7487	11.6517	-3.2817	18.0000	2.0133
BELARUS	2016Q2	1.1561	0.2424	0.7487	11.6523	-2.8866	18.0000	2.0053
BELARUS	2016Q3	0.5414	0.2473	0.7487	11.0527	-2.6365	18.0000	1.9264
BELARUS	2016Q4	-0.1073	0.2491	0.7487	10.4306	-2.5265	18.0000	1.9585
BELARUS	2017Q1	-0.1178	0.2497	0.7487	7.3310	-1.1475	11.0000	1.8720
BELARUS	2017Q2	-0.1599	0.2499	0.7487	6.1179	0.1205	11.0000	1.9336
BELARUS	2017Q3	-0.2325	0.2500	0.7487	5.2309	1.3026	11.0000	1.9689
BELARUS	2017Q4	-0.3340	0.2500	0.7487	4.8033	2.4219	11.0000	1.9727
BELARUS	2018Q1	-0.2481	0.2515	0.7487	6.2029	0.1017	10.4173	1.5743
BELARUS	2018Q2	-0.1880	0.2539	0.7487	5.7345	-0.0294	10.0048	1.6006
BELARUS	2018Q3	-0.1515	0.2569	0.7487	5.2661	-0.1605	9.5923	1.6268
BELARUS	2018Q4	-0.1367	0.2603	0.7487	4.7977	-0.2916	9.1798	1.6530
BELARUS	2019Q1	-0.1972	0.2639	0.7487	4.3293	-0.4227	8.7673	1.6793
BELARUS	2019Q2	-0.2751	0.2677	0.7487	3.8610	-0.5538	8.3548	1.7055
BELARUS	2019Q3	-0.3679	0.2716	0.7487	3.3926	-0.6849	7.9423	1.7317
BELARUS	2019Q4	-0.4731	0.2756	0.7487	2.9242	-0.8159	7.5297	1.7580
BELARUS	2020Q1	0.2082	0.2797	0.7487	2.4558	-0.9470	7.1172	1.7842
BELARUS	2020Q2	0.8828	0.2838	0.7487	1.9874	-1.0781	6.7047	1.8104
BELARUS	2020Q3	1.5536	0.2881	0.7487	1.5190	-1.2092	6.2922	1.8367
BELARUS	2020Q4	2.2231	0.2924	0.7487	1.0506	-1.3403	5.8797	1.8629
BELARUS	2021Q1	0.0000	0.2627	0.7487	8.4800	3.2916	-3.8238	2.5181
BELARUS	2021Q2	0.0000	0.2655	0.7487	9.9100	2.3139	-3.5892	2.5615
BELARUS	2021Q3	0.0000	0.2682	0.7487	10.0200	1.8112	-2.4767	2.5010
BELARUS	2021Q4	0.0000	0.2710	0.7487	9.9700	1.7833	-0.4863	2.5399
BHUTAN	2000Q1	-0.6136	0.0833	0.3389	5.9145	7.6169	8.2500	43.6200
BHUTAN	2000Q2	-0.5716	0.0833	0.3389	5.2344	7.6169	8.2500	44.6800
BHUTAN	2000Q3	-0.5498	0.0833	0.3389	4.6315	7.6168	8.2500	46.0700
BHUTAN	2000Q4	-0.5509	0.0833	0.3389	4.0100	6.9330	8.2500	46.7500
BHUTAN	2001Q1	-0.4665	0.0833	0.3389	4.0448	7.6168	8.2500	46.6400
BHUTAN	2001Q2	-0.4093	0.0833	0.3389	3.9679	7.6169	8.2500	47.0400
BHUTAN	2001Q3	-0.3810	0.0833	0.3389	3.7669	7.6170	8.2500	47.8600
BHUTAN	2001Q4	-0.3830	0.0833	0.3389	3.4100	8.2038	8.2500	48.1800
BHUTAN	2002Q1	-0.3115	0.0833	0.3389	3.5006	7.6170	7.5000	48.8000
BHUTAN	2002Q2	-0.2724	0.0833	0.3389	3.3929	7.6169	7.5000	48.8700
BHUTAN	2002Q3	-0.2665	0.0833	0.3389	3.0698	7.6172	7.5000	48.3800
BHUTAN	2002Q4	-0.2943	0.0833	0.3389	2.4800	10.7278	7.5000	48.0300
BHUTAN	2003Q1	-0.2165	0.0833	0.3389	2.7495	7.6172	7.0000	47.5500

BHUTAN	2003Q2	-0.1731	0.0833	0.3389	2.7014	7.6169	7.0000	46.4700
BHUTAN	2003Q3	-0.1642	0.0833	0.3389	2.3280	7.6169	7.0000	45.8500
BHUTAN	2003Q4	-0.1898	0.0833	0.3389	1.5700	7.6643	7.0000	45.6050
BHUTAN	2004Q1	-0.0008	0.0833	0.3389	-1.2562	7.6169	5.0000	43.4450
BHUTAN	2004Q2	0.1541	0.0833	0.3389	-5.0366	7.6169	5.0000	45.9750
BHUTAN	2004Q3	0.2751	0.0833	0.3389	-10.3719	7.6167	5.0000	46.1550
BHUTAN	2004Q4	0.3624	0.0833	0.3389	-18.1100	5.8964	5.0000	43.5850
BHUTAN	2005Q1	0.2592	0.0833	0.3389	-9.7209	7.6167	4.5000	43.7550
BHUTAN	2005Q2	0.1220	0.0833	0.3389	-3.6311	7.6169	4.5000	43.5150
BHUTAN	2005Q3	-0.0496	0.0833	0.3389	1.1272	7.6168	4.5000	43.9900
BHUTAN	2005Q4	-0.2562	0.0833	0.3389	5.3100	7.1226	4.5000	45.0650
BHUTAN	2006Q1	-0.2196	0.0833	0.3389	5.1501	7.6168	4.5000	44.6050
BHUTAN	2006Q2	-0.2188	0.0833	0.3389	5.0540	7.6169	4.5000	45.0850
BHUTAN	2006Q3	-0.2539	0.0833	0.3389	5.0065	7.6168	4.5000	45.9550
BHUTAN	2006Q4	-0.3248	0.0833	0.3389	5.0000	6.8494	4.5000	44.2450
BHUTAN	2007Q1	-0.7784	0.0833	0.3389	4.9804	7.6168	4.5000	43.5950
BHUTAN	2007Q2	-1.2675	0.0833	0.3389	4.9976	7.6169	4.5000	40.7550
BHUTAN	2007Q3	-1.7911	0.0833	0.3389	5.0545	7.6179	4.5000	39.7350
BHUTAN	2007Q4	-2.3478	0.0833	0.3389	5.1600	17.9258	4.5000	39.4150
BHUTAN	2008Q1	-1.7556	0.0833	0.3389	5.6342	7.6179	2.0000	39.9850
BHUTAN	2008Q2	-1.1895	0.0833	0.3389	6.2491	7.6169	2.0000	42.9500
BHUTAN	2008Q3	-0.6442	0.0833	0.3389	7.1025	7.6166	2.0000	46.9350
BHUTAN	2008Q4	-0.1137	0.0833	0.3389	8.3300	4.7684	2.0000	48.4550
BHUTAN	2009Q1	-0.6425	0.0833	0.3389	6.9632	7.6166	2.0000	50.9450
BHUTAN	2009Q2	-1.1732	0.0833	0.3389	5.9484	7.6169	2.0000	47.8800
BHUTAN	2009Q3	-1.6988	0.0833	0.3389	5.1243	7.6168	2.0000	48.0400
BHUTAN	2009Q4	-2.2117	0.0833	0.3389	4.3600	6.6572	2.0000	46.6800
BHUTAN	2010Q1	-1.0276	0.0833	0.5426	4.8998	7.6168	2.0000	45.1350
BHUTAN	2010Q2	0.1880	0.0833	0.5426	5.4636	7.6169	2.0000	46.6000
BHUTAN	2010Q3	1.4458	0.0833	0.5426	6.1411	7.6173	2.0000	44.9200
BHUTAN	2010Q4	2.7566	0.0833	0.5426	7.0400	11.7309	2.0000	44.8100
BHUTAN	2011Q1	3.1963	0.0833	0.5426	6.9227	7.6173	4.5000	44.6500
BHUTAN	2011Q2	3.7065	0.0833	0.5426	7.1510	7.6169	4.5000	44.7200
BHUTAN	2011Q3	4.2934	0.0833	0.5426	7.7611	7.6169	4.5000	48.9253
BHUTAN	2011Q4	4.9605	0.0834	0.5426	8.8500	7.8909	4.5000	53.2600
BHUTAN	2012Q1	4.0509	0.0834	0.5426	8.4365	7.6169	4.5000	51.1565
BHUTAN	2012Q2	3.2204	0.0835	0.5426	8.6092	7.6169	4.5000	56.3090
BHUTAN	2012Q3	2.4643	0.0838	0.5426	9.3954	7.6167	4.5000	52.6970
BHUTAN	2012Q4	1.7762	0.0846	0.5426	10.9200	5.0717	4.5000	54.7773
BHUTAN	2013Q1	1.4142	0.0871	0.5426	9.0751	7.6167	5.5000	54.3893
BHUTAN	2013Q2	1.1026	0.0940	0.5426	7.9177	7.6169	5.5000	59.6995
BHUTAN	2013Q3	0.8312	0.1137	0.5426	7.2640	7.6164	5.5000	62.7770
BHUTAN	2013Q4	0.5890	0.1699	0.5426	7.0100	2.1425	5.5000	61.8970
BHUTAN	2014Q1	-0.0483	0.3301	0.5426	6.8027	7.6164	6.0000	60.0998
BHUTAN	2014Q2	-0.6795	0.3863	0.5426	6.9218	7.6169	6.0000	60.0933
BHUTAN	2014Q3	-1.3164	0.4060	0.5426	7.3863	7.6167	6.0000	61.6135
BHUTAN	2014Q4	-1.9701	0.4129	0.5426	8.2700	5.7455	6.0000	63.3315
BHUTAN	2015Q1	-1.8614	0.4154	0.5426	6.9581	7.6167	6.0000	62.5908
BHUTAN	2015Q2	-1.7893	0.4162	0.5426	5.9973	7.6169	6.0000	63.7549
BHUTAN	2015Q3	-1.7620	0.4165	0.5426	5.2350	7.6168	6.0000	65.7418
BHUTAN	2015Q4	-1.7865	0.4166	0.5426	4.5500	6.5974	6.0000	66.3260
BHUTAN	2016Q1	-1.7898	0.4166	0.5426	4.5456	7.6168	6.0000	66.3329
BHUTAN	2016Q2	-1.8555	0.4167	0.5426	4.5091	7.6169	6.0000	67.6166
BHUTAN	2016Q3	-1.9872	0.4167	0.5426	4.4345	7.6169	6.0000	66.6596
BHUTAN	2016Q4	-2.1874	0.4167	0.5426	4.3100	7.9907	6.0000	67.9547
BHUTAN	2017Q1	-2.1169	0.4167	0.5426	4.3143	7.6169	6.0000	64.8386
BHUTAN	2017Q2	-2.1160	0.4167	0.5426	4.2497	7.6169	6.0000	64.7379
BHUTAN	2017Q3	-2.1832	0.4167	0.5426	4.1059	7.6168	6.0000	65.3552
BHUTAN	2017Q4	-2.3158	0.4167	0.5426	3.8600	6.8188	6.0000	63.9273
BHUTAN	2018Q1	-1.5885	0.4185	0.5426	7.4122	7.2835	4.0000	62.7296
BHUTAN	2018Q2	-0.9169	0.4214	0.5426	7.4956	7.2745	4.0000	63.0484

BHUTAN	2018Q3	-0.2943	0.4249	0.5426	7.5790	7.2655	4.0000	63.3671
BHUTAN	2018Q4	0.2863	0.4288	0.5426	7.6624	7.2565	4.0000	63.6859
BHUTAN	2019Q1	0.7083	0.4330	0.5426	7.7458	7.2475	4.0000	64.0047
BHUTAN	2019Q2	1.1029	0.4373	0.5426	7.8292	7.2385	4.0000	64.3234
BHUTAN	2019Q3	1.4767	0.4417	0.5426	7.9126	7.2295	4.0000	64.6422
BHUTAN	2019Q4	1.8359	0.4461	0.5426	7.9960	7.2205	4.0000	64.9609
BHUTAN	2020Q1	1.7389	0.4506	0.5426	8.0794	7.2115	4.0000	65.2797
BHUTAN	2020Q2	1.6362	0.4551	0.5426	8.1628	7.2025	4.0000	65.5984
BHUTAN	2020Q3	1.5309	0.4595	0.5426	8.2462	7.1935	4.0000	65.9172
BHUTAN	2020Q4	1.4246	0.4640	0.5426	8.3296	7.1845	4.0000	66.2360
BHUTAN	2021Q1	0.1532	0.4317	0.5426	9.1100	3.5838	7.0000	73.4230
BHUTAN	2021Q2	0.1583	0.4372	0.5426	7.4200	3.9643	7.0000	74.3460
BHUTAN	2021Q3	0.1634	0.4427	0.5426	4.9700	4.2783	7.0000	74.5190
BHUTAN	2021Q4	0.1685	0.4482	0.5426	6.8700	4.5258	7.0000	74.6920
DJIBOUTI	2000Q1	-4.4208	0.0000	0.6260	1.1463	1.2210	11.5000	177.7200
DJIBOUTI	2000Q2	-2.6890	0.0000	0.6260	2.0101	1.2582	11.5000	177.7200
DJIBOUTI	2000Q3	-0.9602	0.0000	0.6260	2.5642	1.2965	11.5000	177.7200
DJIBOUTI	2000Q4	0.7597	0.0000	0.6260	2.6481	0.7000	11.5000	177.7200
DJIBOUTI	2001Q1	0.2569	0.0000	0.6260	1.8928	0.8021	11.5000	177.7200
DJIBOUTI	2001Q2	-0.2652	0.0000	0.6260	1.5142	0.9052	11.5000	177.7200
DJIBOUTI	2001Q3	-0.8125	0.0000	0.6260	2.0769	1.0095	11.5000	177.7200
DJIBOUTI	2001Q4	-1.3904	0.0000	0.6260	1.4450	1.0784	11.5000	177.7200
DJIBOUTI	2002Q1	-1.1087	0.0000	0.6260	0.8190	1.1519	11.2500	177.7200
DJIBOUTI	2002Q2	-0.8669	0.0000	0.6260	0.3587	1.2305	11.2500	177.7200
DJIBOUTI	2002Q3	-0.6683	0.0000	0.6260	0.2281	1.3145	11.2500	177.7200
DJIBOUTI	2002Q4	-0.5159	0.0000	0.6260	1.1347	1.4042	11.2500	177.7200
DJIBOUTI	2003Q1	-0.3542	0.0000	0.6260	2.2901	1.5000	11.2500	177.7200
DJIBOUTI	2003Q2	-0.2433	0.0000	0.6260	2.0300	1.6023	11.2500	177.7200
DJIBOUTI	2003Q3	-0.1853	0.0000	0.6260	1.7425	1.7116	11.2500	177.7200
DJIBOUTI	2003Q4	-0.1820	0.0000	0.6260	1.7892	1.8284	11.2500	177.7200
DJIBOUTI	2004Q1	-0.1502	0.0000	0.6260	2.7054	1.9531	11.2500	177.7200
DJIBOUTI	2004Q2	-0.1763	0.0000	0.6260	2.9234	2.0864	11.2500	177.7200
DJIBOUTI	2004Q3	-0.2618	0.0000	0.6260	3.4584	2.2287	11.2500	177.7200
DJIBOUTI	2004Q4	-0.4082	0.0000	0.6260	3.2095	2.3807	11.2500	177.7200
DJIBOUTI	2005Q1	-0.5076	0.0000	0.6984	2.3011	2.5432	11.2500	177.7200
DJIBOUTI	2005Q2	-0.6699	0.0000	0.6984	2.8704	2.7167	11.2500	177.7200
DJIBOUTI	2005Q3	-0.8959	0.0000	0.6984	3.5816	2.9020	11.2500	177.7200
DJIBOUTI	2005Q4	-1.1857	0.0000	0.6984	3.4658	3.1000	11.2500	177.7200
DJIBOUTI	2006Q1	-1.2528	0.0000	0.6984	3.5457	3.5176	11.2500	177.7200
DJIBOUTI	2006Q2	-1.3823	0.0000	0.6984	3.6937	3.9398	11.2500	177.7200
DJIBOUTI	2006Q3	-1.5724	0.0000	0.6984	3.1404	4.3671	11.2500	177.7200
DJIBOUTI	2006Q4	-1.8203	0.0000	0.6984	3.3210	4.8000	11.2500	177.7200
DJIBOUTI	2007Q1	-1.5645	0.0000	0.6984	3.1126	4.8406	11.2500	177.7200
DJIBOUTI	2007Q2	-1.3580	0.0000	0.6984	3.6461	4.8874	11.2500	177.7200
DJIBOUTI	2007Q3	-1.1949	0.0000	0.6984	5.3717	4.9405	11.2500	177.7200
DJIBOUTI	2007Q4	-1.0685	0.0000	0.6984	7.1587	5.0000	11.2500	177.7200
DJIBOUTI	2008Q1	-1.1028	0.0000	0.6984	9.2106	5.1897	11.5000	177.7200
DJIBOUTI	2008Q2	-1.1582	0.0000	0.6984	11.4078	5.3861	11.5000	177.7200
DJIBOUTI	2008Q3	-1.2262	0.0000	0.6984	13.5275	5.5894	11.5000	177.7200
DJIBOUTI	2008Q4	-1.2971	0.0000	0.6984	10.9300	5.8000	11.5000	177.7200
DJIBOUTI	2009Q1	-0.5613	0.0000	0.6984	5.8552	4.7422	11.0000	177.7200
DJIBOUTI	2009Q2	0.1929	0.0000	0.6984	1.3140	3.6905	11.0000	177.7200
DJIBOUTI	2009Q3	0.9769	0.0000	0.6984	-1.1547	2.6435	11.0000	177.7200
DJIBOUTI	2009Q4	1.8020	0.0000	0.6984	0.8097	1.6000	11.0000	177.7200
DJIBOUTI	2010Q1	2.3669	0.0000	0.6984	2.9343	2.2199	10.2500	177.7200
DJIBOUTI	2010Q2	2.9931	0.0000	0.6984	4.4559	2.8427	10.2500	177.7200
DJIBOUTI	2010Q3	3.6888	0.0000	0.6984	5.1862	3.4691	10.2500	177.7200
DJIBOUTI	2010Q4	4.4602	0.0000	0.6984	2.9218	4.1000	10.2500	177.7200
DJIBOUTI	2011Q1	3.8202	0.0000	0.6984	3.7679	4.8895	10.2500	177.7200
DJIBOUTI	2011Q2	3.2609	0.0000	0.6984	4.6887	5.6853	10.2500	177.7200
DJIBOUTI	2011Q3	2.7811	0.0000	0.6984	4.4026	6.4885	10.2500	177.7200

DJIBOUTI	2011Q4	2.3775	0.0000	0.6984	6.8544	7.3000	10.2500	177.7200
DJIBOUTI	2012Q1	2.1010	0.0000	0.6984	4.9125	6.6629	11.5000	177.7200
DJIBOUTI	2012Q2	1.8890	0.0000	0.6984	4.1156	6.0344	11.5000	177.7200
DJIBOUTI	2012Q3	1.7339	0.0000	0.6984	4.0978	5.4137	11.5000	177.7200
DJIBOUTI	2012Q4	1.6266	0.0000	0.6984	1.5976	4.8000	11.5000	177.7200
DJIBOUTI	2013Q1	0.3598	0.0000	0.6984	3.5650	4.8406	12.0000	177.7200
DJIBOUTI	2013Q2	-0.8805	0.0000	0.6984	3.6595	4.8874	12.0000	177.8050
DJIBOUTI	2013Q3	-2.1055	0.0000	0.6984	1.5714	4.9405	12.0000	177.3800
DJIBOUTI	2013Q4	-3.3261	0.0000	0.6984	1.9119	5.0000	12.0000	178.4950
DJIBOUTI	2014Q1	-2.8516	0.0000	0.6984	1.1778	5.9673	12.7500	177.1015
DJIBOUTI	2014Q2	-2.3895	0.0000	0.6984	1.2635	6.9424	12.7500	177.6385
DJIBOUTI	2014Q3	-1.9455	0.0001	0.6984	0.1648	7.9264	12.7500	177.9950
DJIBOUTI	2014Q4	-1.5236	0.0003	0.6984	2.7075	8.9206	12.7500	177.4876
DJIBOUTI	2015Q1	-1.2718	0.0009	0.6984	-1.0132	9.0936	12.5000	176.9520
DJIBOUTI	2015Q2	-1.0469	0.0027	0.6984	-0.6920	9.2782	12.5000	176.9520
DJIBOUTI	2015Q3	-0.8503	0.0076	0.6984	0.6610	9.4749	12.5000	177.5550
DJIBOUTI	2015Q4	-0.6823	0.0217	0.6984	-2.3456	9.6838	12.5000	177.6350
DJIBOUTI	2016Q1	-0.7066	0.0617	0.6984	1.8183	9.4255	11.5000	177.6350
DJIBOUTI	2016Q2	-0.7592	0.0757	0.6984	2.5994	9.1793	11.5000	177.6750
DJIBOUTI	2016Q3	-0.8390	0.0807	0.6984	3.0004	8.9450	11.5000	177.7700
DJIBOUTI	2016Q4	-0.9447	0.0824	0.6984	3.3702	8.7223	11.5000	179.0784
DJIBOUTI	2017Q1	-0.5713	0.0830	0.6984	3.1540	7.5511	11.2500	180.4257
DJIBOUTI	2017Q2	-0.2194	0.0832	0.6984	0.7811	6.3898	11.2500	178.7750
DJIBOUTI	2017Q3	0.1138	0.0833	0.6984	-0.6895	5.2366	11.2500	178.6048
DJIBOUTI	2017Q4	0.4313	0.0833	0.6984	-0.9603	4.0903	11.2500	178.7350
DJIBOUTI	2018Q1	0.4489	0.0838	0.6984	2.5332	8.3345	11.6565	178.0032
DJIBOUTI	2018Q2	0.4563	0.0845	0.6984	2.5224	8.4421	11.6638	178.0090
DJIBOUTI	2018Q3	0.4560	0.0854	0.6984	2.5116	8.5497	11.6712	178.0148
DJIBOUTI	2018Q4	0.4500	0.0864	0.6984	2.5008	8.6573	11.6785	178.0207
DJIBOUTI	2019Q1	0.4863	0.0874	0.6984	2.4900	8.7650	11.6858	178.0265
DJIBOUTI	2019Q2	0.5202	0.0885	0.6984	2.4793	8.8726	11.6932	178.0324
DJIBOUTI	2019Q3	0.5530	0.0896	0.6984	2.4685	8.9802	11.7005	178.0382
DJIBOUTI	2019Q4	0.5854	0.0907	0.6984	2.4577	9.0878	11.7078	178.0441
DJIBOUTI	2020Q1	0.3779	0.0918	0.6984	2.4469	9.1954	11.7151	178.0499
DJIBOUTI	2020Q2	0.1707	0.0929	0.6984	2.4361	9.3031	11.7225	178.0558
DJIBOUTI	2020Q3	-0.0362	0.0941	0.6984	2.4253	9.4107	11.7298	178.0616
DJIBOUTI	2020Q4	-0.2429	0.0952	0.6984	2.4145	9.5183	11.7371	178.0675
DJIBOUTI	2021Q1	0.3793	0.0688	0.6984	0.5000	3.9000	11.0000	177.5000
DJIBOUTI	2021Q2	0.3927	0.0700	0.6984	0.5000	3,9	11.0000	177.5000
DJIBOUTI	2021Q3	0.4062	0.0711	0.6984	2.5500	3.9000	11.0000	177.5000
DJIBOUTI	2021Q4	0.4196	0.0722	0.6984	2.5500	3.9000	11.0000	177.5000
Dominican Republic	2000Q1	-3.6470	0.0000	0.6250	5.8398	5.6013	18.2500	16.0500
Dominican Republic	2000Q2	-2.9684	0.0000	0.6250	5.9670	5.2985	18.2500	16.0500
Dominican Republic	2000Q3	-2.2678	0.0001	0.6250	9.4479	4.9969	18.2500	16.4300
Dominican Republic	2000Q4	-1.5371	0.0003	0.6250	8.4141	4.6618	18.2500	16.5600
Dominican Republic	2001Q1	-0.8237	0.0009	0.6250	10.7267	4.3314	13.5000	16.6600
Dominican Republic	2001Q2	-0.0600	0.0027	0.6250	10.6665	3.8910	13.5000	16.6600
Dominican Republic	2001Q3	0.7652	0.0076	0.6250	7.7655	3.2901	13.5000	16.6600
Dominican Republic	2001Q4	1.6634	0.0217	0.6250	5.0525	2.4595	13.5000	16.9700
Dominican Republic	2002Q1	2.0248	0.0617	0.6482	2.8431	3.2582	14.5000	17.1500
Dominican Republic	2002Q2	2.4770	0.0758	0.6482	3.7208	3.8236	14.5000	17.7600
Dominican Republic	2002Q3	3.0256	0.0808	0.6482	4.8699	4.2207	14.5000	17.7600
Dominican Republic	2002Q4	3.6750	0.0827	0.6482	8.7696	4.4951	14.5000	17.7600
Dominican Republic	2003Q1	3.6843	0.0839	0.6482	15.4747	3.4931	24.2500	23.9600
Dominican Republic	2003Q2	3.7936	0.0859	0.6482	19.4606	2.2848	24.2500	31.2000
Dominican Republic	2003Q3	3.9970	0.0909	0.6482	27.8625	0.7313	24.2500	32.8200
Dominican Republic	2003Q4	4.2868	0.1050	0.6482	32.5805	-1.3457	24.2500	35.0600
Dominican Republic	2004Q1	1.8407	0.1450	0.6482	45.9453	0.3633	7.0000	44.3500
Dominican Republic	2004Q2	-0.5586	0.1591	0.6482	48.7540	1.5063	7.0000	47.2100
Dominican Republic	2004Q3	-2.9419	0.1640	0.6482	41.6938	2.2148	7.0000	34.7700
Dominican Republic	2004Q4	-0.9890	0.1657	0.6482	31.1230	2.5701	7.0000	31.0500

Dominican Republic	2005Q1	-1.4209	0.1663	0.6482	9.1796	4.2750	10.0000	28.4800
Dominican Republic	2005Q2	-1.8760	0.1666	0.6482	1.2370	5.8633	10.0000	29.0400
Dominican Republic	2005Q3	-2.3631	0.1666	0.6482	0.9859	7.5177	10.0000	31.3520
Dominican Republic	2005Q4	-2.8897	0.1667	0.6482	5.2559	9.4282	10.0000	34.4988
Dominican Republic	2006Q1	-2.7033	0.1667	0.6482	7.8751	8.7805	8.0000	32.5349
Dominican Republic	2006Q2	-2.5669	0.1667	0.6482	9.0262	8.5341	8.0000	32.9450
Dominican Republic	2006Q3	-2.4830	0.1667	0.6482	7.8612	8.6605	8.0000	33.6410
Dominican Republic	2006Q4	-2.4528	0.1667	0.6482	4.5335	9.1744	8.0000	33.7751
Dominican Republic	2007Q1	-1.7325	0.1667	0.6482	4.7874	8.2757	7.0000	32.5390
Dominican Republic	2007Q2	-1.0631	0.1667	0.6482	5.8137	7.7203	7.0000	33.4019
Dominican Republic	2007Q3	-0.4416	0.1668	0.6482	5.3227	7.4443	7.0000	33.5530
Dominican Republic	2007Q4	0.1359	0.1670	0.6482	7.8642	7.4160	7.0000	34.1666
Dominican Republic	2008Q1	0.1742	0.1676	0.6482	8.7408	7.0268	9.5000	34.0506
Dominican Republic	2008Q2	0.1766	0.1693	0.6482	10.4597	7.7120	9.5000	34.3477
Dominican Republic	2008Q3	0.1470	0.1743	0.6482	13.3581	-0.0369	9.5000	35.0112
Dominican Republic	2008Q4	0.0892	0.1883	0.6482	7.8351	-1.6085	9.5000	35.3894
Dominican Republic	2009Q1	0.2081	0.2283	0.6482	3.3227	-2.9277	4.0000	35.9139
Dominican Republic	2009Q2	0.3061	0.2424	0.6482	0.4720	-2.4021	4.0000	36.0175
Dominican Republic	2009Q3	0.3867	0.2473	0.6482	-1.0945	2.3694	4.0000	36.1359
Dominican Republic	2009Q4	0.4534	0.2491	0.6482	3.1140	7.0832	4.0000	36.1201
Dominican Republic	2010Q1	0.5934	0.2497	0.6482	6.3413	8.8751	5.0000	36.4251
Dominican Republic	2010Q2	0.7252	0.2499	0.6482	6.9327	8.5249	5.0000	36.8644
Dominican Republic	2010Q3	0.8514	0.2500	0.6482	5.0931	8.3786	5.0000	37.2053
Dominican Republic	2010Q4	0.9738	0.2500	0.6482	5.7999	7.6224	5.0000	37.5386
Dominican Republic	2011Q1	0.8525	0.2500	0.6482	7.0856	4.4208	6.8000	37.9015
Dominican Republic	2011Q2	0.7298	0.2500	0.6482	8.2419	3.2583	6.8000	38.0719
Dominican Republic	2011Q3	0.6060	0.2500	0.6482	9.7798	2.8005	6.8000	38.2812
Dominican Republic	2011Q4	0.4808	0.2500	0.6482	8.3650	2.1062	6.8000	38.8226
Dominican Republic	2012Q1	0.2018	0.2500	0.6482	6.0050	3.1391	5.0000	39.0917
Dominican Republic	2012Q2	-0.0798	0.2500	0.6482	3.1155	2.4514	5.0000	39.1419
Dominican Republic	2012Q3	-0.3651	0.2500	0.6482	2.1506	2.7637	5.0000	39.3358
Dominican Republic	2012Q4	-0.6549	0.2500	0.6482	3.0397	2.5241	5.0000	40.3967
Dominican Republic	2013Q1	-0.6228	0.2500	0.6482	4.4985	1.6496	6.2500	41.1154
Dominican Republic	2013Q2	-0.5961	0.2500	0.6482	4.7913	4.2104	6.2500	41.9305
Dominican Republic	2013Q3	-0.5749	0.2500	0.6482	5.0381	6.2340	6.2500	42.5303
Dominican Republic	2013Q4	-0.5586	0.2500	0.6482	4.1061	7.3750	6.2500	42.7902
Dominican Republic	2014Q1	-0.3170	0.2500	0.6482	3.1749	7.6479	6.2500	43.1474
Dominican Republic	2014Q2	-0.0784	0.2500	0.6482	3.4486	7.8034	6.2500	43.5113
Dominican Republic	2014Q3	0.1588	0.2500	0.6482	3.1297	7.8154	6.2500	43.8223
Dominican Republic	2014Q4	0.3959	0.2500	0.6482	2.2728	7.2959	6.2500	44.3563
Dominican Republic	2015Q1	0.3715	0.2500	0.6482	0.5666	6.9204	5.0000	44.7502
Dominican Republic	2015Q2	0.3495	0.2500	0.6482	0.2821	7.3759	5.0000	44.9670
Dominican Republic	2015Q3	0.3307	0.2500	0.6482	0.5587	7.8442	5.0000	45.3107
Dominican Republic	2015Q4	0.3158	0.2500	0.6482	1.8728	6.0387	5.0000	45.5544
Dominican Republic	2016Q1	0.3360	0.2500	0.6482	2.0578	6.2215	5.5000	45.8319
Dominican Republic	2016Q2	0.3607	0.2500	0.6482	1.8559	8.5919	5.5000	45.9726
Dominican Republic	2016Q3	0.3901	0.2500	0.6482	1.3804	6.3524	5.5000	46.4484
Dominican Republic	2016Q4	0.4238	0.2500	0.6482	1.0476	5.3153	5.5000	46.7062
Dominican Republic	2017Q1	0.3428	0.2500	0.6482	2.8965	5.5245	5.7500	47.3587
Dominican Republic	2017Q2	0.2650	0.2500	0.6482	3.0097	3.1173	5.7500	47.5271
Dominican Republic	2017Q3	0.1895	0.2500	0.6482	3.1239	3.1143	5.7500	47.7401
Dominican Republic	2017Q4	0.1154	0.2500	0.6482	3.8664	6.4706	5.7500	48.2993
Dominican Republic	2018Q1	-0.0741	0.2515	0.6482	4.1664	5.2457	1.4375	48.0283
Dominican Republic	2018Q2	-0.2648	0.2539	0.6482	4.1514	5.8246	1.4375	48.5222
Dominican Republic	2018Q3	-0.4579	0.2567	0.6482	4.1365	6.4036	1.4375	49.0161
Dominican Republic	2018Q4	-0.6545	0.2597	0.6482	4.1215	6.9825	1.4375	49.5100
Dominican Republic	2019Q1	-0.6048	0.2630	0.6482	3.7225	6.4999	1.4375	49.9562
Dominican Republic	2019Q2	-0.5598	0.2663	0.6482	3.3236	6.0173	1.4375	50.4024
Dominican Republic	2019Q3	-0.5194	0.2696	0.6482	2.9246	5.5348	1.4375	50.8486
Dominican Republic	2019Q4	-0.4832	0.2730	0.6482	2.5256	5.0522	1.4375	51.2949
Dominican Republic	2020Q1	-0.1297	0.2764	0.6482	3.0751	2.1091	1.4375	52.6023

Dominican Republic	2020Q2	0.2213	0.2798	0.6482	3.6246	-0.8340	1.4375	53.9097
Dominican Republic	2020Q3	0.5707	0.2832	0.6482	4.1740	-3.7771	1.4375	55.2171
Dominican Republic	2020Q4	0.9195	0.2866	0.6482	4.7235	-6.7202	1.4375	56.5245
Dominican Republic	2021Q1	0.0000	0.3229	0.6482	8.3000	3.1000	3.0000	56.6250
Dominican Republic	2021Q2	0.0000	0.3259	0.6482	9.3200	25.4000	3.0000	54.4620
Dominican Republic	2021Q3	0.0000	0.3290	0.6482	7.7400	11.4000	3.0000	56.1060
Dominican Republic	2021Q4	0.0000	0.3320	0.6482	8.5000	11.1000	3.0000	57.1440
EGYPT	2000Q1	1.8591	0.0000	0.4926	2.9290	5.8495	12.0000	3.4130
EGYPT	2000Q2	1.1062	0.0000	0.4926	2.7123	5.6478	12.0000	3.4460
EGYPT	2000Q3	0.4188	0.0000	0.4926	2.6105	5.4900	12.0000	3.5010
EGYPT	2000Q4	-0.2028	0.0000	0.4926	2.3478	5.3680	12.0000	3.6900
EGYPT	2001Q1	-0.0017	0.0000	0.4926	2.3317	4.9222	11.0000	3.8400
EGYPT	2001Q2	0.2657	0.0000	0.4926	2.2099	4.4832	11.0000	3.8500
EGYPT	2001Q3	0.5997	0.0000	0.4926	2.1425	4.0286	11.0000	4.1400
EGYPT	2001Q4	1.0004	0.0000	0.4926	2.2940	3.5353	11.0000	4.4900
EGYPT	2002Q1	0.6541	0.0000	0.4926	2.5697	3.3665	10.0000	4.5000
EGYPT	2002Q2	0.3734	0.0000	0.4926	2.6100	3.1250	10.0000	4.5000
EGYPT	2002Q3	0.1570	0.0000	0.4926	2.7000	2.7986	10.0000	4.5000
EGYPT	2002Q4	0.0034	0.0000	0.4926	2.9186	2.3705	10.0000	4.5000
EGYPT	2003Q1	-0.2097	0.0000	0.4926	3.1476	3.3066	10.0000	5.7284
EGYPT	2003Q2	-0.3633	0.0000	0.4926	3.8927	3.3191	10.0000	6.0329
EGYPT	2003Q3	-0.4588	0.0000	0.4926	4.6737	2.8970	10.0000	6.1257
EGYPT	2003Q4	-0.4976	0.0000	0.4926	5.8724	3.2432	10.0000	6.1532
EGYPT	2004Q1	-0.2549	0.0000	0.4875	9.1229	4.2677	10.0000	6.1680
EGYPT	2004Q2	0.0428	0.0000	0.4875	11.2729	4.2487	10.0000	6.1899
EGYPT	2004Q3	0.3952	0.0000	0.4875	11.0459	3.8582	10.0000	6.2290
EGYPT	2004Q4	0.8016	0.0000	0.4875	11.2168	3.9791	10.0000	6.1314
EGYPT	2005Q1	0.5055	0.0000	0.4875	7.0812	3.9070	10.0000	5.7881
EGYPT	2005Q2	0.2614	0.0000	0.4875	4.7261	4.4732	10.0000	5.7789
EGYPT	2005Q3	0.0676	0.0000	0.4875	4.1568	4.6185	10.0000	5.7517
EGYPT	2005Q4	-0.0775	0.0000	0.4875	3.1530	4.9345	10.0000	5.7322
EGYPT	2006Q1	0.0748	0.0000	0.4875	3.6261	6.0877	9.0000	5.7366
EGYPT	2006Q2	0.2722	0.0000	0.4875	5.5223	6.7555	9.0000	5.7523
EGYPT	2006Q3	0.5129	0.0000	0.4875	8.5743	6.8138	9.0000	5.7318
EGYPT	2006Q4	0.7947	0.0000	0.4875	11.4437	7.7735	9.0000	5.7036
EGYPT	2007Q1	0.6402	0.0000	0.4875	11.8137	6.9198	9.0000	5.6925
EGYPT	2007Q2	0.5219	0.0000	0.4875	9.4462	11.2225	9.0000	5.6892
EGYPT	2007Q3	0.4365	0.0000	0.4875	7.9415	12.3056	9.0000	5.5776
EGYPT	2007Q4	0.3805	0.0000	0.4875	6.6650	16.7676	9.0000	5.5038
EGYPT	2008Q1	0.6147	0.0000	0.4875	11.6159	5.9172	11.5000	5.4424
EGYPT	2008Q2	0.8705	0.0000	0.4875	17.2093	12.4522	11.5000	5.3236
EGYPT	2008Q3	1.1436	0.0000	0.4875	20.1932	14.2312	11.5000	5.4530
EGYPT	2008Q4	1.4288	0.0000	0.4875	17.9017	6.6008	11.5000	5.5041
EGYPT	2009Q1	0.7239	0.0000	0.4875	12.5146	4.3169	8.5000	5.6289
EGYPT	2009Q2	0.0190	0.0000	0.4875	10.1128	4.6139	8.5000	5.5855
EGYPT	2009Q3	-0.6930	0.0000	0.4875	9.4590	4.6301	8.5000	5.4897
EGYPT	2009Q4	-1.4189	0.0000	0.4875	12.4332	4.9732	8.5000	5.4754
EGYPT	2010Q1	-1.2445	0.0000	0.4875	12.1258	5.5988	8.5000	5.4913
EGYPT	2010Q2	-1.0963	0.0000	0.4875	9.9198	5.3853	8.5000	5.6807
EGYPT	2010Q3	-0.9795	0.0000	0.4875	10.7801	5.4745	8.5000	5.6832
EGYPT	2010Q4	-0.8982	0.0000	0.4875	9.9877	5.6201	8.5000	5.7926
EGYPT	2011Q1	-0.5803	0.0000	0.4875	10.3998	-4.3338	9.7500	5.9539
EGYPT	2011Q2	-0.3047	0.0000	0.4875	11.2429	0.3524	9.7500	5.9558
EGYPT	2011Q3	-0.0740	0.0000	0.4875	8.6314	0.2595	9.7500	5.9525
EGYPT	2011Q4	0.1093	0.0000	0.4875	8.1944	-0.2199	9.7500	6.0169
EGYPT	2012Q1	-0.2197	0.0000	0.4875	8.5714	5.4044	9.7500	6.0264
EGYPT	2012Q2	-0.6012	0.0000	0.4875	7.8086	1.3257	9.7500	6.0455
EGYPT	2012Q3	-1.0376	0.0000	0.4875	6.1443	2.5616	9.7500	6.0801
EGYPT	2012Q4	-1.5309	0.0000	0.4875	5.0946	2.1850	9.7500	6.3057
EGYPT	2013Q1	-1.4325	0.0000	0.4875	7.1119	2.1877	8.7500	6.7947
EGYPT	2013Q2	-1.3927	0.0000	0.4875	8.3350	1.4735	8.7500	7.0119

EGYPT	2013Q3	-1.4112	0.0000	0.4875	9.3827	1.0427	8.7500	6.8838
EGYPT	2013Q4	-1.4867	0.0000	0.4875	11.0612	1.4421	8.7500	6.9430
EGYPT	2014Q1	-1.4792	0.0000	0.4875	9.8091	2.0040	9.7500	6.9615
EGYPT	2014Q2	-1.5234	0.0001	0.4875	8.1000	2.4237	9.7500	7.1459
EGYPT	2014Q3	-1.6149	0.0002	0.4875	10.8492	2.7226	9.7500	7.1470
EGYPT	2014Q4	-1.7490	0.0007	0.4875	9.8408	2.9159	9.7500	7.1431
EGYPT	2015Q1	-1.5880	0.0019	0.4875	10.0734	3.3698	9.2500	7.6032
EGYPT	2015Q2	-1.4564	0.0053	0.4875	11.1782	3.7513	9.2500	7.6061
EGYPT	2015Q3	-1.3459	0.0152	0.4875	8.1481	4.0798	9.2500	7.8057
EGYPT	2015Q4	-1.2475	0.0433	0.4875	10.0870	4.3720	9.2500	7.8080
EGYPT	2016Q1	0.6259	0.1234	0.4875	8.9804	4.3958	15.2500	8.8572
EGYPT	2016Q2	2.5077	0.1515	0.4875	11.4909	4.3996	15.2500	8.8571
EGYPT	2016Q3	4.4081	0.1613	0.4875	13.5466	4.3834	15.2500	8.8574
EGYPT	2016Q4	6.3359	0.1648	0.4875	17.1932	4.3466	15.2500	18.1250
EGYPT	2017Q1	4.9387	0.1660	0.4875	26.0623	4.3413	19.2500	18.0835
EGYPT	2017Q2	3.5769	0.1664	0.4875	26.4667	4.3131	19.2500	18.0400
EGYPT	2017Q3	2.2492	0.1666	0.4875	27.8813	4.2606	19.2500	17.6059
EGYPT	2017Q4	0.9524	0.1666	0.4875	23.1852	4.1812	19.2500	17.6808
EGYPT	2018Q1	0.3423	0.1676	0.4875	22.5560	4.4644	4.3125	17.7787
EGYPT	2018Q2	-0.2469	0.1690	0.4875	22.1794	4.7477	4.3125	17.7749
EGYPT	2018Q3	-0.8210	0.1708	0.4875	21.8028	5.0309	4.3125	17.7711
EGYPT	2018Q4	-1.3853	0.1728	0.4875	21.4262	5.3141	4.3125	17.7673
EGYPT	2019Q1	-1.5824	0.1749	0.4875	19.4753	5.3750	3.1875	17.5181
EGYPT	2019Q2	-1.7787	0.1770	0.4875	17.5244	5.4359	3.1875	17.2689
EGYPT	2019Q3	-1.9770	0.1792	0.4875	15.5735	5.4968	3.1875	17.0198
EGYPT	2019Q4	-2.1795	0.1814	0.4875	13.6226	5.5577	3.1875	16.7706
EGYPT	2020Q1	-1.1962	0.1837	0.4875	11.6135	5.0607	2.1875	16.5177
EGYPT	2020Q2	-0.2169	0.1859	0.4875	9.6044	4.5637	2.1875	16.2649
EGYPT	2020Q3	0.7597	0.1881	0.4875	7.5953	4.0667	2.1875	16.0120
EGYPT	2020Q4	1.7352	0.1904	0.4875	5.5862	3.5697	2.1875	15.7592
EGYPT	2021Q1	-0.1751	0.1377	0.4875	4.5000	1.6000	8.2500	15.5290
EGYPT	2021Q2	-0.1802	0.1399	0.4875	4.9000	7.3000	8.2500	15.6010
EGYPT	2021Q3	-0.1853	0.1422	0.4875	6.6000	2.5000	8.2500	15.6730
EGYPT	2021Q4	-0.1905	0.1445	0.4875	5.9000	2.0000	8.2500	15.5860
INDONESIA	2000Q1	1.7237	0.0000	0.9040	-0.5957	4.1051	14.5000	7590.0000
INDONESIA	2000Q2	1.3354	0.0000	0.9040	1.0871	4.5911	14.5000	8735.0000
INDONESIA	2000Q3	0.9118	0.0000	0.9040	5.5250	5.0226	14.5000	8780.0000
INDONESIA	2000Q4	0.4505	0.0000	0.9040	8.4003	6.1886	14.5000	9595.0000
INDONESIA	2001Q1	0.0248	0.0000	0.9040	8.9241	3.8683	17.5000	10400.0000
INDONESIA	2001Q2	-0.4454	0.0000	0.9040	10.5761	5.7700	17.5000	11440.0000
INDONESIA	2001Q3	-0.9634	0.0000	0.9040	12.0089	3.4417	17.5000	9675.0000
INDONESIA	2001Q4	-1.5327	0.0000	0.9040	11.9046	1.5616	17.5000	10400.0000
INDONESIA	2002Q1	-1.3857	0.0000	0.9040	13.5828	3.5201	13.0000	9655.0000
INDONESIA	2002Q2	-1.2928	0.0000	0.9040	11.9479	4.2126	13.0000	8730.0000
INDONESIA	2002Q3	-1.2534	0.0000	0.9040	9.8740	5.5514	13.0000	9015.0000
INDONESIA	2002Q4	-1.2660	0.0000	0.9040	9.7300	4.6837	13.0000	8940.0000
INDONESIA	2003Q1	-1.0216	0.0000	0.9040	7.5126	4.9080	8.2500	8908.0000
INDONESIA	2003Q2	-0.8236	0.0000	0.9040	6.9666	5.0302	8.2500	8285.0000
INDONESIA	2003Q3	-0.6679	0.0001	0.9040	6.1829	4.5597	8.2500	8389.0000
INDONESIA	2003Q4	-0.5501	0.0003	0.9040	5.5392	4.6320	8.2500	8465.0000
INDONESIA	2004Q1	0.0788	0.0009	0.8460	4.7257	4.0992	7.5000	8587.0000
INDONESIA	2004Q2	0.6811	0.0027	0.8460	6.1883	4.3878	7.5000	9415.0000
INDONESIA	2004Q3	1.2632	0.0076	0.8460	6.4944	4.4981	7.5000	9170.0000
INDONESIA	2004Q4	1.8309	0.0217	0.8460	6.1151	7.1585	7.5000	9290.0000
INDONESIA	2005Q1	1.8059	0.0617	0.8460	7.4620	5.9650	12.7500	9480.0000
INDONESIA	2005Q2	1.7738	0.0757	0.8460	7.3819	5.8713	12.7500	9713.0000
INDONESIA	2005Q3	1.7359	0.0807	0.8460	8.0632	5.8382	12.7500	10310.0000
INDONESIA	2005Q4	1.6921	0.0824	0.8460	16.3669	5.1066	12.7500	9830.0000
INDONESIA	2006Q1	1.1068	0.0830	0.8460	15.6183	5.1272	9.7500	9075.0000
INDONESIA	2006Q2	0.5110	0.0832	0.8460	14.4219	4.9334	9.7500	9300.0000
INDONESIA	2006Q3	-0.0993	0.0833	0.8460	13.8647	5.8640	9.7500	9235.0000

INDONESIA	2006Q4	-0.7290	0.0833	0.8460	5.8763	6.0564	9.7500	9020.0000
INDONESIA	2007Q1	-0.5454	0.0833	0.8460	6.1656	6.0551	8.0000	9118.0000
INDONESIA	2007Q2	-0.3879	0.0833	0.8460	5.8498	6.7268	8.0000	9054.0000
INDONESIA	2007Q3	-0.2583	0.0833	0.8460	6.3039	6.7444	8.0000	9137.0000
INDONESIA	2007Q4	-0.1584	0.0833	0.8460	6.5076	5.8422	8.0000	9419.0000
INDONESIA	2008Q1	-0.0428	0.0833	0.8460	7.3680	6.2184	9.2500	9217.0000
INDONESIA	2008Q2	0.0401	0.0833	0.8460	9.7242	6.3027	9.2500	9225.0000
INDONESIA	2008Q3	0.0887	0.0833	0.8460	11.2375	6.2549	9.2500	9378.0000
INDONESIA	2008Q4	0.1014	0.0833	0.8460	10.5126	5.2825	9.2500	10950.0000
INDONESIA	2009Q1	-0.4766	0.0833	0.8460	7.3750	4.5204	6.5000	11575.0000
INDONESIA	2009Q2	-1.0952	0.0833	0.8460	4.6505	4.1358	6.5000	10225.0000
INDONESIA	2009Q3	-1.7573	0.0833	0.8460	2.7280	4.2693	6.5000	9681.0000
INDONESIA	2009Q4	0.2990	0.0833	0.8460	2.5555	5.6003	6.5000	9400.0000
INDONESIA	2010Q1	-0.1524	0.0833	0.8460	3.5919	5.9893	6.5000	9115.0000
INDONESIA	2010Q2	-0.6284	0.0834	0.8460	4.2786	7.7239	6.5000	9083.0000
INDONESIA	2010Q3	-1.1294	0.0834	0.8460	5.9687	7.7072	6.5000	8924.0000
INDONESIA	2010Q4	-1.6555	0.0837	0.8460	6.1249	7.9564	6.5000	8991.0000
INDONESIA	2011Q1	-1.3970	0.0843	0.8460	6.6074	6.4770	6.0000	8709.0000
INDONESIA	2011Q2	-1.1610	0.0860	0.8460	5.7247	6.2685	6.0000	8597.0000
INDONESIA	2011Q3	-0.9448	0.0909	0.8460	4.5622	6.0131	6.0000	8823.0000
INDONESIA	2011Q4	-0.7449	0.1050	0.8460	4.0357	5.9424	6.0000	9068.0000
INDONESIA	2012Q1	-0.4268	0.1450	0.8460	3.6572	6.1101	5.7500	9180.0000
INDONESIA	2012Q2	-0.1166	0.1591	0.8460	4.3970	6.2078	5.7500	9480.0000
INDONESIA	2012Q3	0.1903	0.1641	0.8460	4.3880	5.9400	5.7500	9588.0000
INDONESIA	2012Q4	0.4987	0.1661	0.8460	4.3122	5.8706	5.7500	9670.0000
INDONESIA	2013Q1	0.6314	0.1673	0.8460	4.6178	5.5409	7.5000	9719.0000
INDONESIA	2013Q2	0.7746	0.1692	0.8460	5.0969	5.5882	7.5000	9929.0000
INDONESIA	2013Q3	0.9323	0.1742	0.8460	7.5773	5.5159	7.5000	11613.0000
INDONESIA	2013Q4	1.1079	0.1883	0.8460	7.4684	5.5846	7.5000	12189.0000
INDONESIA	2014Q1	0.7399	0.2284	0.8460	7.4749	5.1159	7.7500	11404.0000
INDONESIA	2014Q2	0.3949	0.2424	0.8460	6.8494	4.9376	7.7500	11969.0000
INDONESIA	2014Q3	0.0747	0.2474	0.8460	4.2595	4.9318	7.7500	12212.0000
INDONESIA	2014Q4	-0.2192	0.2494	0.8460	6.2747	5.0477	7.7500	12440.0000
INDONESIA	2015Q1	0.0971	0.2506	0.8460	6.3378	4.8312	7.5000	13084.0000
INDONESIA	2015Q2	0.4427	0.2526	0.8460	6.8266	4.7403	7.5000	13332.0000
INDONESIA	2015Q3	0.8192	0.2576	0.8460	6.8492	4.7795	7.5000	14657.0000
INDONESIA	2015Q4	1.2278	0.2716	0.8460	4.6996	5.1526	7.5000	13795.0000
INDONESIA	2016Q1	1.0663	0.3117	0.8460	4.2438	4.9415	4.7500	13276.0000
INDONESIA	2016Q2	0.9376	0.3257	0.8460	3.4026	5.2136	4.7500	13180.0000
INDONESIA	2016Q3	0.8409	0.3307	0.8460	2.9799	5.0346	4.7500	12998.0000
INDONESIA	2016Q4	0.7751	0.3324	0.8460	3.2492	4.9414	4.7500	13436.0000
INDONESIA	2017Q1	0.4998	0.3330	0.8460	3.5805	5.0116	4.2500	13321.0000
INDONESIA	2017Q2	0.2512	0.3332	0.8460	4.2020	5.0098	4.2500	13319.0000
INDONESIA	2017Q3	0.0267	0.3333	0.8460	3.7357	5.0603	4.2500	13492.0000
INDONESIA	2017Q4	-0.1765	0.3333	0.8460	3.4384	5.1855	4.2500	13548.0000
INDONESIA	2018Q1	-0.1754	0.3393	0.8460	4.1741	5.0959	4.2500	13594.8601
INDONESIA	2018Q2	-0.1585	0.3440	0.8460	4.0555	5.1220	4.2500	13808.8863
INDONESIA	2018Q3	-0.1282	0.3444	0.8460	3.9369	5.1482	5.2500	14022.9125
INDONESIA	2018Q4	-0.0872	0.3437	0.8460	3.8183	5.1743	5.2500	14236.9388
INDONESIA	2019Q1	-0.3186	0.3457	0.8460	3.2634	5.1353	6.0000	14214.6219
INDONESIA	2019Q2	-0.5439	0.3521	0.8460	2.7086	5.0962	6.0000	14192.3051
INDONESIA	2019Q3	-0.7652	0.3613	0.8460	2.1537	5.0572	5.5000	14169.9882
INDONESIA	2019Q4	-0.9844	0.3701	0.8460	1.5988	5.0182	5.0000	14147.6714
INDONESIA	2020Q1	-0.7024	0.3755	0.8460	1.0851	3.2462	5.0000	14256.3044
INDONESIA	2020Q2	-0.4203	0.3774	0.8460	0.5713	1.4743	4.5000	14364.9374
INDONESIA	2020Q3	-0.1382	0.3779	0.8460	0.0576	-0.2976	4.0000	14473.5704
INDONESIA	2020Q4	0.1437	0.3799	0.8460	-0.4561	-2.0695	4.0000	14582.2035
INDONESIA	2021Q1	0.0000	0.3704	0.8460	1.3700	-0.7100	3.5000	14490.4000
INDONESIA	2021Q2	0.0000	0.3756	0.8460	1.3300	7.0700	3.5000	14471.1000
INDONESIA	2021Q3	0.0000	0.3808	0.8460	1.6000	3.5100	3.5000	14240.4000
INDONESIA	2021Q4	0.0000	0.3860	0.8460	1.8700	5.0200	3.5000	14250.0000

KAZAKHSTAN	2000Q1	1.5139	0.0833	0.3709	18.3646	4.4851	14.0000	141.8000
KAZAKHSTAN	2000Q2	1.2678	0.0833	0.3709	12.6074	6.2455	14.0000	142.6000
KAZAKHSTAN	2000Q3	1.0162	0.0833	0.3709	9.3595	8.0082	14.0000	142.7500
KAZAKHSTAN	2000Q4	0.7576	0.0833	0.3709	9.6798	9.8000	14.0000	144.5000
KAZAKHSTAN	2001Q1	0.8204	0.0833	0.3709	8.4818	10.6078	9.0000	145.4500
KAZAKHSTAN	2001Q2	0.8718	0.0833	0.3709	9.1322	11.4846	9.0000	146.5000
KAZAKHSTAN	2001Q3	0.9088	0.0833	0.3709	7.9119	12.4438	9.0000	147.7000
KAZAKHSTAN	2001Q4	0.9279	0.0833	0.3709	6.6194	13.5000	9.0000	150.2000
KAZAKHSTAN	2002Q1	0.4071	0.0833	0.3709	5.4113	12.4438	7.5000	152.2000
KAZAKHSTAN	2002Q2	-0.1405	0.0833	0.3709	5.1754	11.4846	7.5000	153.1000
KAZAKHSTAN	2002Q3	-0.7198	0.0833	0.3709	6.0715	10.6078	7.5000	154.5500
KAZAKHSTAN	2002Q4	-1.3360	0.0833	0.3709	6.0191	9.8000	7.5000	154.6000
KAZAKHSTAN	2003Q1	-1.7977	0.0833	0.5699	6.8082	9.5968	7.0000	151.5000
KAZAKHSTAN	2003Q2	-2.3043	0.0833	0.5699	6.1025	9.4472	7.0000	148.0000
KAZAKHSTAN	2003Q3	-2.8584	0.0833	0.5699	5.3746	9.3487	7.0000	148.9300
KAZAKHSTAN	2003Q4	-3.4611	0.0833	0.5699	6.6717	9.3000	7.0000	144.2200
KAZAKHSTAN	2004Q1	-3.7961	0.0833	0.5699	6.2441	9.3006	7.0000	138.8800
KAZAKHSTAN	2004Q2	-4.1759	0.0833	0.5699	6.4405	9.3502	7.0000	136.4500
KAZAKHSTAN	2004Q3	-4.5952	0.0833	0.5699	7.1181	9.4494	7.0000	134.5600
KAZAKHSTAN	2004Q4	-5.0459	0.0833	0.5699	6.8077	9.6000	7.0000	130.0000
KAZAKHSTAN	2005Q1	-4.4186	0.0833	0.5699	6.7316	9.5457	8.0000	132.5900
KAZAKHSTAN	2005Q2	-3.7976	0.0833	0.5699	7.4819	9.5442	8.0000	135.2600
KAZAKHSTAN	2005Q3	-3.1656	0.0833	0.5699	7.8628	9.5954	8.0000	133.8900
KAZAKHSTAN	2005Q4	-2.5031	0.0833	0.5699	7.5119	9.7000	8.0000	133.9800
KAZAKHSTAN	2006Q1	-1.0579	0.0833	0.5699	8.3359	9.8601	9.0000	128.4500
KAZAKHSTAN	2006Q2	0.4625	0.0833	0.5699	8.7618	10.0778	9.0000	118.6900
KAZAKHSTAN	2006Q3	2.0823	0.0833	0.5699	8.3626	10.3563	9.0000	127.1200
KAZAKHSTAN	2006Q4	3.8252	0.0833	0.5699	7.9998	10.7000	9.0000	127.0000
KAZAKHSTAN	2007Q1	5.4450	0.0833	0.5699	7.7664	10.1638	11.0000	123.7500
KAZAKHSTAN	2007Q2	7.2302	0.0833	0.5699	7.5726	9.6897	11.0000	121.6600
KAZAKHSTAN	2007Q3	9.1973	0.0833	0.5699	9.4644	9.2706	11.0000	121.2000
KAZAKHSTAN	2007Q4	11.3585	0.0833	0.5699	15.9386	8.9000	11.0000	120.3000
KAZAKHSTAN	2008Q1	8.5573	0.0833	0.5699	17.1702	7.4894	10.5000	120.6900
KAZAKHSTAN	2008Q2	5.9538	0.0833	0.5699	17.8338	6.1000	10.5000	120.7500
KAZAKHSTAN	2008Q3	3.5399	0.0833	0.5699	17.7292	4.7106	10.5000	119.8100
KAZAKHSTAN	2008Q4	1.3039	0.0833	0.5699	10.9206	3.3000	10.5000	120.7900
KAZAKHSTAN	2009Q1	1.6691	0.0833	0.5699	8.3333	2.8572	7.0000	151.4000
KAZAKHSTAN	2009Q2	2.1844	0.0833	0.5699	7.9003	2.3648	7.0000	150.4300
KAZAKHSTAN	2009Q3	2.8349	0.0833	0.5699	6.2129	1.8152	7.0000	150.9500
KAZAKHSTAN	2009Q4	3.6042	0.0833	0.5699	5.8834	1.2000	7.0000	148.4600
KAZAKHSTAN	2010Q1	1.5384	0.0833	0.5699	7.3053	2.7780	7.0000	146.9800
KAZAKHSTAN	2010Q2	-0.4478	0.0833	0.5699	7.0397	4.3051	7.0000	147.5500
KAZAKHSTAN	2010Q3	-2.3769	0.0833	0.5699	6.7076	5.8048	7.0000	147.5700
KAZAKHSTAN	2010Q4	-4.2708	0.0833	0.5699	7.5030	7.3000	7.0000	147.5000
KAZAKHSTAN	2011Q1	-4.4719	0.0833	0.5699	8.2936	7.2972	7.5000	145.7000
KAZAKHSTAN	2011Q2	-4.6757	0.0834	0.5699	8.1254	7.3127	7.5000	145.8300
KAZAKHSTAN	2011Q3	-4.8965	0.0835	0.5699	8.5577	7.3468	7.5000	147.9900
KAZAKHSTAN	2011Q4	-5.1458	0.0837	0.5699	7.4962	7.4000	7.5000	148.4000
KAZAKHSTAN	2012Q1	-4.2278	0.0843	0.5699	4.9836	6.7451	5.5000	147.7700
KAZAKHSTAN	2012Q2	-3.3516	0.0860	0.5699	4.8225	6.1000	5.5000	149.4200
KAZAKHSTAN	2012Q3	-2.5194	0.0910	0.5699	4.7891	5.4549	5.5000	149.8600
KAZAKHSTAN	2012Q4	-1.7314	0.1053	0.5699	5.6521	4.8000	5.5000	150.7400
KAZAKHSTAN	2013Q1	-1.4430	0.1460	0.5699	6.7124	5.1179	5.5000	150.8400
KAZAKHSTAN	2013Q2	-1.1947	0.1617	0.5699	6.0474	5.4209	5.5000	151.6500
KAZAKHSTAN	2013Q3	-0.9834	0.1716	0.5699	5.5825	5.7134	5.5000	153.8100
KAZAKHSTAN	2013Q4	-0.8049	0.1874	0.5699	4.7866	6.0000	5.5000	154.0600
KAZAKHSTAN	2014Q1	-0.7568	0.2280	0.5699	5.3519	5.5690	5.5000	182.0600
KAZAKHSTAN	2014Q2	-0.7317	0.2423	0.5699	6.7379	5.1298	5.5000	183.5200
KAZAKHSTAN	2014Q3	-0.7239	0.2473	0.5699	7.0035	4.6758	5.5000	181.9000
KAZAKHSTAN	2014Q4	-0.7275	0.2490	0.5699	7.3682	4.2000	5.5000	182.3500
KAZAKHSTAN	2015Q1	-0.2920	0.2497	0.5699	6.1071	3.5204	16.0000	185.6500

KAZAKHSTAN	2015Q2	0.1459	0.2498	0.5699	4.2562	2.8013	16.0000	186.2000
KAZAKHSTAN	2015Q3	0.5935	0.2498	0.5699	3.9907	2.0318	16.0000	270.8900
KAZAKHSTAN	2015Q4	4.7818	0.2497	0.5699	11.2323	1.2000	16.0000	340.0100
KAZAKHSTAN	2016Q1	3.9621	0.2491	0.5699	13.9782	1.2856	12.0000	343.6200
KAZAKHSTAN	2016Q2	3.1658	0.2473	0.5699	15.2718	1.2975	12.0000	338.6600
KAZAKHSTAN	2016Q3	2.3944	0.2424	0.5699	15.7363	1.2360	12.0000	335.4600
KAZAKHSTAN	2016Q4	1.6473	0.2284	0.5699	8.9600	1.1000	12.0000	333.2900
KAZAKHSTAN	2017Q1	1.1363	0.1883	0.5699	7.4049	1.9100	10.2500	313.7300
KAZAKHSTAN	2017Q2	0.6443	0.1743	0.5699	7.2727	2.6558	10.2500	322.2700
KAZAKHSTAN	2017Q3	0.1674	0.1694	0.5699	6.8406	3.3490	10.2500	341.1900
KAZAKHSTAN	2017Q4	-0.2985	0.1679	0.5699	7.2029	4.0000	10.2500	332.3300
KAZAKHSTAN	2018Q1	-0.6111	0.1436	0.5699	7.8316	2.1482	9.0000	246.3831
KAZAKHSTAN	2018Q2	-0.9213	0.1406	0.5699	7.8212	2.0228	9.0000	248.4627
KAZAKHSTAN	2018Q3	-1.2329	0.1390	0.5699	7.8108	1.8973	9.0000	250.5422
KAZAKHSTAN	2018Q4	-1.5491	0.1398	0.5699	7.8003	1.7718	9.0000	252.6218
KAZAKHSTAN	2019Q1	-1.4752	0.1406	0.5699	7.7899	1.6464	9.0000	254.7014
KAZAKHSTAN	2019Q2	-1.4098	0.1414	0.5699	7.7795	1.5209	9.0000	256.7810
KAZAKHSTAN	2019Q3	-1.3535	0.1422	0.5699	7.7690	1.3954	9.0000	258.8605
KAZAKHSTAN	2019Q4	-1.3060	0.1429	0.5699	7.7586	1.2700	9.0000	260.9401
KAZAKHSTAN	2020Q1	-0.4604	0.1436	0.5699	7.7482	1.1445	9.0000	263.0197
KAZAKHSTAN	2020Q2	0.3797	0.1444	0.5699	7.7377	1.0190	9.0000	265.0993
KAZAKHSTAN	2020Q3	1.2164	0.1451	0.5699	7.7273	0.8936	9.0000	267.1788
KAZAKHSTAN	2020Q4	2.0518	0.1458	0.5699	7.7169	0.7681	9.0000	269.2584
KAZAKHSTAN	2021Q1	0.0000	0.1914	0.5699	7.0000	-1.4000	9.0000	425.7000
KAZAKHSTAN	2021Q2	0.0000	0.1930	0.5699	7.9000	2.4000	9.0000	427.5000
KAZAKHSTAN	2021Q3	0.0000	0.1946	0.5699	8.9000	3.6000	9.5000	425.9100
KAZAKHSTAN	2021Q4	0.0000	0.1962	0.5699	8.4000	4.3000	10.7500	434.8000
KENYA	2000Q1	0.9851	0.0000	0.5374	7.4436	2.3008	20.0000	74.8739
KENYA	2000Q2	0.6889	0.0000	0.5374	8.6419	2.0441	20.0000	77.9528
KENYA	2000Q3	0.4066	0.0000	0.5374	10.8483	1.5012	20.0000	78.9889
KENYA	2000Q4	0.1376	0.0000	0.5374	10.9519	0.5997	20.0000	78.0361
KENYA	2001Q1	-0.0503	0.0000	0.5374	9.9880	1.7723	15.0000	77.8167
KENYA	2001Q2	-0.2271	0.0000	0.5374	6.5912	2.6225	15.0000	78.9850
KENYA	2001Q3	-0.3937	0.0000	0.5374	3.6817	3.2633	15.0000	79.0211
KENYA	2001Q4	-0.5511	0.0000	0.5374	2.3097	3.7799	15.0000	78.6000
KENYA	2002Q1	-0.4081	0.0000	0.5374	1.2034	3.2535	12.0000	78.0578
KENYA	2002Q2	-0.2570	0.0000	0.5374	1.7982	2.6017	12.0000	78.7861
KENYA	2002Q3	-0.0977	0.0000	0.5374	1.8930	1.7378	12.0000	79.0256
KENYA	2002Q4	0.0698	0.0000	0.5374	2.8554	0.5469	12.0000	77.0722
KENYA	2003Q1	-0.0870	0.0000	0.5374	7.6791	1.5820	7.0000	76.6461
KENYA	2003Q2	-0.2354	0.0000	0.5374	12.6113	2.2694	7.0000	74.1667
KENYA	2003Q3	-0.3751	0.0000	0.5374	8.6418	2.7004	7.0000	78.4167
KENYA	2003Q4	-0.5059	0.0000	0.5374	8.4315	2.9325	7.0000	76.1389
KENYA	2004Q1	0.0805	0.0000	0.5374	8.7078	3.5384	2.0000	77.7617
KENYA	2004Q2	0.6772	0.0000	0.5374	5.8623	4.0568	2.0000	79.5128
KENYA	2004Q3	1.2850	0.0000	0.5374	13.4302	4.5566	2.0000	81.1144
KENYA	2004Q4	1.9044	0.0000	0.5374	15.7528	5.1043	2.0000	77.3444
KENYA	2005Q1	1.7493	0.0000	0.5374	13.3791	5.1035	8.0000	75.0167
KENYA	2005Q2	1.6037	0.0000	0.5374	13.2919	5.2233	8.0000	76.2056
KENYA	2005Q3	1.4649	0.0001	0.5374	7.2867	5.4797	8.0000	74.0778
KENYA	2005Q4	1.3292	0.0003	0.5374	5.6100	5.9067	8.0000	72.3667
KENYA	2006Q1	0.5975	0.0009	0.5374	16.3941	5.9779	10.0000	71.8722
KENYA	2006Q2	-0.1412	0.0027	0.5374	12.1836	6.2097	10.0000	73.8800
KENYA	2006Q3	-0.8926	0.0076	0.5374	11.1779	8.1883	10.0000	72.6789
KENYA	2006Q4	-1.6626	0.0217	0.5374	14.2421	4.8990	10.0000	69.3967
KENYA	2007Q1	-1.5700	0.0617	0.5374	7.1539	7.0890	8.7500	68.7811
KENYA	2007Q2	-1.5052	0.0757	0.5374	7.3783	8.3308	8.7500	66.5644
KENYA	2007Q3	-1.4715	0.0807	0.5374	11.8158	6.3452	8.7500	66.9711
KENYA	2007Q4	-1.4709	0.0824	0.5374	10.8656	6.3505	8.7500	62.6750
KENYA	2008Q1	-0.9601	0.0830	0.5374	18.3110	1.1259	8.5000	62.8478
KENYA	2008Q2	-0.4842	0.0832	0.5374	23.8054	2.1921	8.5000	64.6944

KENYA	2008Q3	-0.0430	0.0833	0.5374	25.5861	2.5720	8.5000	73.2189
KENYA	2008Q4	0.3641	0.0833	0.5374	25.2012	0.2426	8.5000	77.7111
KENYA	2009Q1	0.0639	0.0833	0.5374	13.2522	6.1744	7.0000	80.4306
KENYA	2009Q2	-0.2695	0.0833	0.5374	9.6990	1.8659	7.0000	77.1578
KENYA	2009Q3	-0.6359	0.0833	0.5374	7.2323	1.8966	7.0000	74.9994
KENYA	2009Q4	-1.0348	0.0833	0.5374	5.4892	1.2307	7.0000	75.8200
KENYA	2010Q1	-0.8241	0.0833	0.5374	4.9043	1.4340	6.0000	77.3314
KENYA	2010Q2	-0.6435	0.0833	0.5374	3.6083	6.0616	6.0000	81.9167
KENYA	2010Q3	-0.4910	0.0833	0.5374	3.2752	7.2427	6.0000	80.7781
KENYA	2010Q4	-0.3644	0.0833	0.5374	3.7713	8.3061	6.0000	80.7519
KENYA	2011Q1	0.0238	0.0833	0.5096	6.8137	4.8448	18.0000	82.9889
KENYA	2011Q2	0.3919	0.0833	0.5096	12.3658	3.5487	18.0000	89.8639
KENYA	2011Q3	0.7428	0.0833	0.5096	15.2828	4.0003	18.0000	99.8319
KENYA	2011Q4	1.0793	0.0833	0.5096	17.5524	5.1079	18.0000	85.0681
KENYA	2012Q1	0.3082	0.0833	0.5096	15.5764	4.0940	11.0000	83.0556
KENYA	2012Q2	-0.4735	0.0833	0.5096	11.1233	4.4676	11.0000	84.2333
KENYA	2012Q3	-1.2645	0.0833	0.5096	6.1779	4.5302	11.0000	85.2833
KENYA	2012Q4	-2.0632	0.0833	0.5096	3.4650	5.0831	11.0000	86.0008
KENYA	2013Q1	-2.0637	0.0833	0.5096	3.9976	5.0884	8.5000	85.6386
KENYA	2013Q2	-2.0658	0.0833	0.5096	4.2742	4.3224	8.5000	86.0075
KENYA	2013Q3	-2.0643	0.0833	0.5096	6.7618	4.3628	8.5000	86.6458
KENYA	2013Q4	-2.0530	0.0833	0.5096	7.1599	5.8787	8.5000	86.3097
KENYA	2014Q1	-0.9058	0.0833	0.5096	6.5560	5.5079	8.5000	86.4414
KENYA	2014Q2	0.2677	0.0833	0.5096	6.7971	5.3115	8.5000	87.6269
KENYA	2014Q3	1.4770	0.0833	0.5096	7.2663	5.2634	8.5000	89.2794
KENYA	2014Q4	2.7314	0.0833	0.5096	5.9932	5.3571	8.5000	90.5017
KENYA	2015Q1	2.8367	0.0833	0.5096	5.6538	5.2339	11.5000	92.3350
KENYA	2015Q2	3.0022	0.0833	0.5096	6.7606	5.2487	11.5000	98.6394
KENYA	2015Q3	3.2326	0.0833	0.5096	5.9608	5.4033	11.5000	105.2928
KENYA	2015Q4	3.5310	0.0833	0.5096	7.0936	5.7185	11.5000	102.3114
KENYA	2016Q1	3.0032	0.0833	0.5096	6.7837	5.4975	10.0000	101.3339
KENYA	2016Q2	2.5431	0.0833	0.5096	5.2202	5.4495	10.0000	101.1022
KENYA	2016Q3	2.1477	0.0833	0.5096	6.1378	5.5681	10.0000	101.2617
KENYA	2016Q4	1.8123	0.0833	0.5096	6.2970	5.8692	10.0000	102.4858
KENYA	2017Q1	0.9599	0.0833	0.5096	8.4863	5.4150	10.0000	103.0000
KENYA	2017Q2	0.1544	0.0833	0.5096	10.2440	5.1228	10.0000	103.7117
KENYA	2017Q3	-0.6122	0.0833	0.5096	7.2535	4.9538	10.0000	103.2472
KENYA	2017Q4	-1.3476	0.0833	0.5096	4.8601	4.8854	10.0000	103.2317
KENYA	2018Q1	-1.4704	0.0838	0.5096	8.8121	5.1839	2.2500	102.8832
KENYA	2018Q2	-1.5762	0.0845	0.5096	6.6822	5.5621	2.2500	102.3560
KENYA	2018Q3	-1.6706	0.0854	0.5096	4.5523	5.9403	2.2500	101.8288
KENYA	2018Q4	-1.7584	0.0864	0.5096	2.4225	6.3185	2.2500	101.3016
KENYA	2019Q1	-1.5174	0.0874	0.5096	2.8071	6.0803	2.1250	101.4740
KENYA	2019Q2	-1.2760	0.0885	0.5096	3.1918	5.8421	2.1250	101.6464
KENYA	2019Q3	-1.0359	0.0896	0.5096	3.5764	5.6039	2.1250	101.8189
KENYA	2019Q4	-0.7979	0.0907	0.5096	3.9611	5.3657	2.1250	101.9913
KENYA	2020Q1	-0.5626	0.0918	0.5096	5.0600	3.9474	1.7500	103.1062
KENYA	2020Q2	-0.1889	0.0929	0.5096	6.1590	2.5291	1.7500	104.2210
KENYA	2020Q3	0.3117	0.0941	0.5096	7.2580	1.1108	1.7500	105.3359
KENYA	2020Q4	0.9300	0.0952	0.5096	8.3570	-0.3075	1.7500	106.4508
KENYA	2021Q1	-0.0310	0.1149	0.5096	5.9000	2.7000	7.0000	108.5200
KENYA	2021Q2	-0.0319	0.1162	0.5096	6.3200	11.0000	7.0000	107.6600
KENYA	2021Q3	-0.0327	0.1176	0.5096	6.9100	9.3000	7.0000	110.4700
KENYA	2021Q4	-0.0336	0.1190	0.5096	6.7300	7.4000	7.0000	113.0600
MALAYSIA	2000Q1	1.0663	0.0000	0.4734	2.2479	6.8179	0.6650	3.8000
MALAYSIA	2000Q2	-1.0919	0.0000	0.4734	4.4503	7.4982	0.6650	3.8000
MALAYSIA	2000Q3	-3.0976	0.0000	0.4734	6.6528	8.1786	0.6650	3.8000
MALAYSIA	2000Q4	-4.9812	0.0000	0.4734	8.8552	8.8589	0.6650	3.8000
MALAYSIA	2001Q1	-4.7075	0.0000	0.4734	6.2459	6.7736	0.6975	3.8000
MALAYSIA	2001Q2	-4.3657	0.0000	0.4734	3.6367	4.6883	0.6975	3.8000
MALAYSIA	2001Q3	-3.9781	0.0000	0.4734	1.0274	2.6030	0.6975	3.8000

MALAYSIA	2001Q4	-3.5646	0.0000	0.4734	-1.5819	0.5177	0.6975	3.8000
MALAYSIA	2002Q1	-3.4862	0.0000	0.4734	-0.4042	1.7360	0.6825	3.8000
MALAYSIA	2002Q2	-3.4141	0.0000	0.4734	0.7735	2.9543	0.6825	3.8000
MALAYSIA	2002Q3	-3.3611	0.0000	0.4734	1.9512	4.1727	0.6825	3.8000
MALAYSIA	2002Q4	-3.3380	0.0000	0.4734	3.1289	5.3910	0.6825	3.8000
MALAYSIA	2003Q1	-2.2482	0.0000	0.4734	3.1714	5.4904	0.6850	3.8000
MALAYSIA	2003Q2	-1.2033	0.0000	0.4734	3.2139	5.5897	0.6850	3.8000
MALAYSIA	2003Q3	-0.2085	0.0000	0.4734	3.2564	5.6891	0.6850	3.8000
MALAYSIA	2003Q4	0.7318	0.0000	0.4734	3.2989	5.7885	0.6850	3.8000
MALAYSIA	2004Q1	0.5676	0.0000	0.4734	3.9765	6.0372	0.6750	3.8000
MALAYSIA	2004Q2	0.3400	0.0000	0.4734	4.6541	6.2860	0.6750	3.8000
MALAYSIA	2004Q3	0.0440	0.0000	0.4734	5.3317	6.5347	0.6750	3.8000
MALAYSIA	2004Q4	-0.3257	0.0000	0.4734	6.0093	6.7834	0.6750	3.8000
MALAYSIA	2005Q1	-0.3686	0.0000	0.4734	6.7226	6.4206	0.6800	3.7968
MALAYSIA	2005Q2	-0.4956	0.0000	0.4734	7.4359	6.0578	0.6800	3.7935
MALAYSIA	2005Q3	-0.7115	0.0000	0.4734	8.1493	5.6950	0.6800	3.7903
MALAYSIA	2005Q4	-1.0208	0.0000	0.4734	8.8626	5.3321	0.6800	3.7871
MALAYSIA	2006Q1	-0.7896	0.0000	0.4734	7.6422	5.3953	0.8450	3.7574
MALAYSIA	2006Q2	-0.6594	0.0000	0.4734	6.4218	5.4585	0.8450	3.7276
MALAYSIA	2006Q3	-0.6332	0.0000	0.4734	5.2013	5.5217	0.8450	3.6979
MALAYSIA	2006Q4	-0.7135	0.0000	0.4734	3.9809	5.5848	0.8450	3.6682
MALAYSIA	2007Q1	-0.7084	0.0000	0.4734	4.2060	5.7633	0.8750	3.6105
MALAYSIA	2007Q2	-0.8137	0.0000	0.4734	4.4311	5.9418	0.8750	3.5529
MALAYSIA	2007Q3	-1.0307	0.0000	0.4734	4.6562	6.1203	0.8750	3.4952
MALAYSIA	2007Q4	-1.3601	0.0000	0.4734	4.8813	6.2988	0.8750	3.4376
MALAYSIA	2008Q1	-2.4891	0.0000	0.4734	6.2582	5.9320	0.8675	3.4121
MALAYSIA	2008Q2	-3.7300	0.0000	0.4734	7.6351	5.5653	0.8675	3.3867
MALAYSIA	2008Q3	-5.0805	0.0001	0.4734	9.0120	5.1985	0.8675	3.3613
MALAYSIA	2008Q4	-6.5359	0.0005	0.4734	10.3889	4.8318	0.8675	3.3358
MALAYSIA	2009Q1	-3.1658	0.0020	0.4992	6.2936	3.2454	0.5300	3.3830
MALAYSIA	2009Q2	0.1190	0.0076	0.5249	2.1983	1.6591	0.5300	3.4302
MALAYSIA	2009Q3	3.3324	0.0283	0.5507	-1.8969	0.0728	0.5300	3.4773
MALAYSIA	2009Q4	6.4881	0.1057	0.5765	-5.9922	-1.5135	0.5300	3.5245
MALAYSIA	2010Q1	4.7623	0.3943	0.5765	-2.6774	0.7211	0.6125	3.4486
MALAYSIA	2010Q2	2.9981	0.4717	0.5765	0.6373	2.9557	0.6125	3.3728
MALAYSIA	2010Q3	1.2003	0.4924	0.5765	3.9521	5.1903	0.6125	3.2969
MALAYSIA	2010Q4	-0.6283	0.4980	0.5765	7.2668	7.4248	0.6125	3.2211
MALAYSIA	2011Q1	-1.0388	0.4995	0.5765	6.8032	6.8921	0.7200	3.1808
MALAYSIA	2011Q2	-1.4757	0.4999	0.5765	6.3396	6.3594	0.7200	3.1405
MALAYSIA	2011Q3	-1.9358	0.5000	0.5765	5.8760	5.8266	0.7200	3.1003
MALAYSIA	2011Q4	-2.4152	0.5000	0.5765	5.4124	5.2939	0.7200	3.0600
MALAYSIA	2012Q1	-1.8097	0.5000	0.5765	4.3093	5.3388	0.7250	3.0672
MALAYSIA	2012Q2	-1.2116	0.5000	0.5765	3.2062	5.3837	0.7250	3.0744
MALAYSIA	2012Q3	-0.6129	0.5000	0.5765	2.1031	5.4286	0.7250	3.0816
MALAYSIA	2012Q4	-0.0050	0.5000	0.5765	0.9999	5.4735	0.7250	3.0888
MALAYSIA	2013Q1	0.6402	0.5000	0.5765	0.7936	5.2785	0.7475	3.1043
MALAYSIA	2013Q2	1.3126	0.5000	0.5765	0.5872	5.0836	0.7475	3.1199
MALAYSIA	2013Q3	2.0208	0.5000	0.5765	0.3808	4.8887	0.7475	3.1354
MALAYSIA	2013Q4	2.7726	0.5000	0.5765	0.1745	4.6937	0.7475	3.1509
MALAYSIA	2014Q1	2.3004	0.5000	0.5765	0.7477	5.0220	0.7750	3.1814
MALAYSIA	2014Q2	1.8830	0.5000	0.5765	1.3210	5.3502	0.7750	3.2119
MALAYSIA	2014Q3	1.5239	0.5000	0.5765	1.8942	5.6785	0.7750	3.2424
MALAYSIA	2014Q4	1.2252	0.5000	0.5765	2.4675	6.0067	0.7750	3.2729
MALAYSIA	2015Q1	1.4496	0.5000	0.5765	2.1551	5.7779	0.8025	3.4310
MALAYSIA	2015Q2	1.7360	0.5000	0.5765	1.8428	5.5491	0.8025	3.5892
MALAYSIA	2015Q3	2.0841	0.5000	0.5765	1.5304	5.3203	0.8025	3.7473
MALAYSIA	2015Q4	2.4922	0.5000	0.5765	1.2181	5.0915	0.8025	3.9055
MALAYSIA	2016Q1	2.0454	0.5000	0.5765	1.3281	4.9311	0.7650	3.9662
MALAYSIA	2016Q2	1.6514	0.5000	0.5765	1.4382	4.7707	0.7650	4.0269
MALAYSIA	2016Q3	1.3046	0.5000	0.5765	1.5482	4.6102	0.7650	4.0876
MALAYSIA	2016Q4	0.9983	0.5000	0.5765	1.6583	4.4498	0.7650	4.1483

MALAYSIA	2017Q1	-0.1973	0.5000	0.5765	2.1884	4.7905	0.7500	4.1863
MALAYSIA	2017Q2	-1.3680	0.5000	0.5765	2.7186	5.1313	0.7500	4.2244
MALAYSIA	2017Q3	-2.5220	0.5000	0.5765	3.2488	5.4720	0.7500	4.2624
MALAYSIA	2017Q4	-3.6665	0.5000	0.5765	3.7790	5.8127	0.7500	4.3004
MALAYSIA	2018Q1	-2.8183	0.5036	0.5765	3.0026	5.5520	0.8125	4.2341
MALAYSIA	2018Q2	-1.9694	0.5089	0.5765	2.2262	5.2913	0.8125	4.1678
MALAYSIA	2018Q3	-1.1213	0.5151	0.5765	1.4499	5.0306	0.8125	4.1015
MALAYSIA	2018Q4	-0.2744	0.5216	0.5765	0.6735	4.7699	0.8125	4.0351
MALAYSIA	2019Q1	-0.0719	0.5283	0.5765	0.5210	4.6531	0.7500	4.0620
MALAYSIA	2019Q2	0.1304	0.5351	0.5765	0.3685	4.5364	0.7500	4.0888
MALAYSIA	2019Q3	0.3331	0.5419	0.5765	0.2161	4.4196	0.7500	4.1156
MALAYSIA	2019Q4	0.5368	0.5488	0.5765	0.0636	4.3028	0.7500	4.1425
MALAYSIA	2020Q1	0.4958	0.5556	0.5765	-0.1471	1.8302	0.7500	4.1577
MALAYSIA	2020Q2	0.2684	0.5625	0.5765	-0.3579	-0.6425	0.7500	4.1730
MALAYSIA	2020Q3	-0.1252	0.5694	0.5765	-0.5686	-3.1151	0.7500	4.1882
MALAYSIA	2020Q4	-0.6661	0.5762	0.5765	-0.7794	-5.5877	0.7500	4.2035
MALAYSIA	2021Q1	0.9079	0.6612	0.5765	1.7000	-0.5000	0.7500	4.1640
MALAYSIA	2021Q2	0.9429	0.6705	0.5765	3.4000	15.9000	0.7500	4.1690
MALAYSIA	2021Q3	0.9780	0.6797	0.5765	2.2000	-4.5000	0.7500	4.1847
MALAYSIA	2021Q4	1.0130	0.6890	0.5765	3.2000	3.8000	0.7500	4.1738
MALDIVES	2000Q1	3.8482	0.0000	0.5765	-1.2757	5.5938	6.0000	11.7700
MALDIVES	2000Q2	3.6442	0.0000	0.5765	-0.2464	5.5937	6.0000	11.7700
MALDIVES	2000Q3	3.4311	0.0000	0.5765	0.3557	5.5934	6.0000	11.7700
MALDIVES	2000Q4	3.2043	0.0000	0.5765	-1.1780	3.8458	6.0000	11.7700
MALDIVES	2001Q1	2.3587	0.0000	0.4157	0.1925	5.5934	6.0000	11.7700
MALDIVES	2001Q2	1.4835	0.0000	0.4157	-3.2319	5.5937	6.0000	11.7700
MALDIVES	2001Q3	0.5677	0.0000	0.4157	0.3886	5.5920	6.0000	12.8000
MALDIVES	2001Q4	-0.4003	0.0000	0.4157	5.4682	-3.9436	6.0000	12.8000
MALDIVES	2002Q1	-0.5779	0.0000	0.4157	8.7702	5.5920	6.0000	12.8000
MALDIVES	2002Q2	-0.8308	0.0000	0.4157	2.3444	5.5937	6.0000	12.8000
MALDIVES	2002Q3	-1.1698	0.0000	0.4157	3.9546	5.5940	6.0000	12.8000
MALDIVES	2002Q4	-1.6049	0.0000	0.4157	1.4054	7.2684	6.0000	12.8000
MALDIVES	2003Q1	-2.8054	0.0000	0.4157	-2.3904	5.5940	5.5000	12.8000
MALDIVES	2003Q2	-4.1206	0.0000	0.4157	-0.8268	5.5937	5.5000	12.8000
MALDIVES	2003Q3	-5.5578	0.0000	0.4157	-0.3324	5.5951	5.5000	12.8000
MALDIVES	2003Q4	-7.1219	0.0000	0.4157	-1.5236	13.7500	5.5000	12.8000
MALDIVES	2004Q1	-6.9049	0.0000	0.4157	-2.0881	5.5951	5.2500	12.8000
MALDIVES	2004Q2	-6.8113	0.0000	0.4157	-1.4375	5.5937	5.2500	12.8000
MALDIVES	2004Q3	-6.8319	0.0000	0.4157	-2.8835	5.5938	5.2500	12.8000
MALDIVES	2004Q4	-6.3233	0.0000	0.4157	-0.3916	6.0338	5.2500	12.8000
MALDIVES	2005Q1	-4.0508	0.0000	0.4157	0.5107	5.5938	3.0000	12.8000
MALDIVES	2005Q2	-1.8375	0.0000	0.4157	0.1526	5.5937	3.0000	12.8000
MALDIVES	2005Q3	0.3394	0.0000	0.4157	1.8827	5.5904	3.0000	12.8000
MALDIVES	2005Q4	2.5041	0.0000	0.4157	2.6005	-13.1291	3.0000	12.8000
MALDIVES	2006Q1	1.6885	0.0000	0.4157	2.5511	5.5904	3.0000	12.8000
MALDIVES	2006Q2	0.9066	0.0000	0.4157	2.8551	5.5937	3.0000	12.8000
MALDIVES	2006Q3	0.1797	0.0000	0.4157	3.0065	5.5973	3.0000	12.8000
MALDIVES	2006Q4	-0.4718	0.0000	0.4157	2.3971	26.1115	3.0000	12.8000
MALDIVES	2007Q1	1.0226	0.0001	0.4282	4.9326	5.5973	3.0000	12.8000
MALDIVES	2007Q2	2.6337	0.0002	0.4282	5.0330	5.5937	3.0000	12.8000
MALDIVES	2007Q3	4.3818	0.0005	0.4282	7.4153	5.5941	3.0000	12.8000
MALDIVES	2007Q4	6.2854	0.0013	0.4282	8.7934	7.7139	3.0000	12.8000
MALDIVES	2008Q1	6.1378	0.0037	0.4282	10.0515	5.5941	3.0000	12.8000
MALDIVES	2008Q2	6.1732	0.0107	0.4282	13.2065	5.5937	3.0000	12.8000
MALDIVES	2008Q3	6.3998	0.0304	0.4282	13.3239	5.5944	3.0000	12.8000
MALDIVES	2008Q4	6.8215	0.0866	0.4282	8.8893	9.4853	3.0000	12.8000
MALDIVES	2009Q1	5.6741	0.2467	0.4282	7.2838	5.5944	5.2500	12.8000
MALDIVES	2009Q2	4.7181	0.3029	0.4282	3.8598	5.5937	5.2500	12.8000
MALDIVES	2009Q3	3.9455	0.3227	0.4282	1.7119	5.5914	5.2500	12.8000
MALDIVES	2009Q4	3.3459	0.3296	0.4282	4.9877	-7.2288	5.2500	12.8000
MALDIVES	2010Q1	2.3704	0.3320	0.4282	4.4486	5.5914	4.5000	12.8000

MALDIVES	2010Q2	1.5396	0.3329	0.4282	5.7317	5.5937	4.5000	12.8000
MALDIVES	2010Q3	0.8368	0.3332	0.4282	7.6227	5.5940	4.5000	12.8000
MALDIVES	2010Q4	0.2444	0.3333	0.4282	6.0120	7.2651	4.5000	12.8000
MALDIVES	2011Q1	-0.0457	0.3333	0.4282	6.2722	5.5940	7.0000	12.8000
MALDIVES	2011Q2	-0.2617	0.3333	0.4282	11.0298	5.5937	7.0000	15.3750
MALDIVES	2011Q3	-0.4221	0.3333	0.4282	10.4909	5.5942	7.0000	15.3900
MALDIVES	2011Q4	-0.5448	0.3333	0.4282	14.5614	8.5667	7.0000	15.4100
MALDIVES	2012Q1	-1.1610	0.3333	0.4282	16.0627	5.5942	7.0000	15.4050
MALDIVES	2012Q2	-1.7748	0.3333	0.4282	9.4412	5.5937	7.0000	15.4050
MALDIVES	2012Q3	-2.4032	0.3333	0.4282	9.7792	5.5932	7.0000	15.4050
MALDIVES	2012Q4	-3.0617	0.3333	0.4282	6.4568	2.5174	7.0000	15.3650
MALDIVES	2013Q1	-2.9127	0.3333	0.4282	4.2185	5.5932	7.0000	15.4100
MALDIVES	2013Q2	-2.8203	0.3333	0.4282	4.2267	5.5937	7.0000	15.3900
MALDIVES	2013Q3	-2.7949	0.3333	0.4282	2.9301	5.5940	7.0000	15.3600
MALDIVES	2013Q4	-2.8450	0.3333	0.4282	3.5873	7.2811	7.0000	15.4100
MALDIVES	2014Q1	-2.5674	0.3333	0.4282	2.9043	5.5940	7.0000	15.4000
MALDIVES	2014Q2	-2.3774	0.3333	0.4282	2.8939	5.5937	7.0000	15.4100
MALDIVES	2014Q3	-2.2786	0.3333	0.4282	2.0390	5.5940	7.0000	15.3900
MALDIVES	2014Q4	-2.2731	0.3333	0.4282	0.5817	7.3296	7.0000	15.4000
MALDIVES	2015Q1	-1.7239	0.3333	0.4282	0.4833	5.5940	7.0000	15.3900
MALDIVES	2015Q2	-1.2678	0.3333	0.4282	1.1018	5.5937	7.0000	15.4100
MALDIVES	2015Q3	-0.9029	0.3333	0.4282	1.0690	5.5931	7.0000	15.3900
MALDIVES	2015Q4	-0.6265	0.3333	0.4282	1.1362	2.2459	7.0000	15.4100
MALDIVES	2016Q1	-0.1781	0.3333	0.4282	0.9443	5.5931	7.0000	15.3500
MALDIVES	2016Q2	0.1886	0.3333	0.4282	-0.3706	5.5937	7.0000	15.3800
MALDIVES	2016Q3	0.4773	0.3333	0.4282	-0.4087	5.5938	7.0000	15.3700
MALDIVES	2016Q4	0.6918	0.3333	0.4282	1.8257	6.1632	7.0000	15.3500
MALDIVES	2017Q1	0.7671	0.3333	0.4282	3.3575	5.5938	7.0000	15.3900
MALDIVES	2017Q2	0.7743	0.3333	0.4282	4.0736	5.5937	7.0000	15.3800
MALDIVES	2017Q3	0.7158	0.3333	0.4282	2.8471	5.5943	7.0000	15.4100
MALDIVES	2017Q4	0.5935	0.3333	0.4282	0.4104	8.8285	7.0000	15.4100
MALDIVES	2018Q1	0.1588	0.3351	0.4282	5.0639	5.8357	7.0000	15.7762
MALDIVES	2018Q2	-0.3372	0.3380	0.4282	5.0996	5.8422	7.0000	15.8335
MALDIVES	2018Q3	-0.8933	0.3416	0.4282	5.1353	5.8487	7.0000	15.8908
MALDIVES	2018Q4	-1.5084	0.3455	0.4282	5.1710	5.8553	7.0000	15.9481
MALDIVES	2019Q1	-2.1025	0.3497	0.4282	5.2066	5.8618	7.0000	16.0055
MALDIVES	2019Q2	-2.7510	0.3540	0.4282	5.2423	5.8683	7.0000	16.0628
MALDIVES	2019Q3	-3.4499	0.3584	0.4282	5.2780	5.8748	7.0000	16.1201
MALDIVES	2019Q4	-4.1936	0.3628	0.4282	5.3137	5.8813	7.0000	16.1774
MALDIVES	2020Q1	-0.8106	0.3672	0.4282	5.3494	5.8879	7.0000	16.2347
MALDIVES	2020Q2	2.5461	0.3717	0.4282	5.3851	5.8944	7.0000	16.2921
MALDIVES	2020Q3	5.8876	0.3762	0.4282	5.4208	5.9009	7.0000	16.3494
MALDIVES	2020Q4	9.2234	0.3807	0.4282	5.4565	5.9074	7.0000	16.4067
MALDIVES	2021Q1	0.0000	0.4507	0.4282	-0.0800	-6.7000	7.0000	15.4190
MALDIVES	2021Q2	0.0000	0.4567	0.4282	0.9300	77.6000	7.0000	15.4220
MALDIVES	2021Q3	0.0000	0.4627	0.4282	0.0800	83.3000	7.0000	15.4200
MALDIVES	2021Q4	0.0000	0.4688	0.4282	0.0200	55.9000	7.0000	15.4210
NEPAL	2000Q1	1.6245	0.1667	0.1791	4.0415	4.1803	7.5000	68.9750
NEPAL	2000Q2	2.0314	0.1667	0.1791	2.0411	4.1803	7.5000	70.7500
NEPAL	2000Q3	2.4827	0.1667	0.1791	0.8552	4.1806	7.5000	73.2500
NEPAL	2000Q4	2.9751	0.1667	0.1791	2.9078	6.2000	7.5000	74.3000
NEPAL	2001Q1	2.7413	0.1667	0.1791	1.9365	4.1806	6.5000	74.0000
NEPAL	2001Q2	2.5368	0.1667	0.1791	2.9978	4.1803	6.5000	74.7500
NEPAL	2001Q3	2.3533	0.1667	0.1791	3.3025	4.1804	6.5000	75.7250
NEPAL	2001Q4	2.1804	0.1667	0.1791	2.3641	4.7998	6.5000	76.4750
NEPAL	2002Q1	0.6838	0.1667	0.6443	3.0988	4.1804	5.5000	76.6800
NEPAL	2002Q2	-0.8266	0.1667	0.6443	2.6280	4.1803	5.5000	78.3000
NEPAL	2002Q3	-2.3641	0.1667	0.6443	3.6065	4.1797	5.5000	78.2950
NEPAL	2002Q4	-3.9414	0.1667	0.6443	2.6029	0.1203	5.5000	78.3000
NEPAL	2003Q1	-3.1149	0.1667	0.6443	4.2699	4.1797	5.5000	78.0450
NEPAL	2003Q2	-2.3485	0.1667	0.6443	7.2085	4.1803	5.5000	76.3900

NEPAL	2003Q3	-1.6491	0.1667	0.6443	5.3924	4.1803	5.5000	74.3200
NEPAL	2003Q4	-1.0224	0.1667	0.6443	5.3106	3.9450	5.5000	74.0400
NEPAL	2004Q1	-1.0524	0.1667	0.6443	4.5762	4.1803	5.5000	73.5500
NEPAL	2004Q2	-1.1632	0.1667	0.6443	1.6105	4.1803	5.5000	73.4500
NEPAL	2004Q3	-1.3580	0.1667	0.6443	2.3180	4.1804	5.5000	74.7850
NEPAL	2004Q4	-1.6392	0.1667	0.6443	2.7613	4.6826	5.5000	71.8000
NEPAL	2005Q1	-1.8399	0.1667	0.6443	6.7788	4.1804	6.0000	70.9500
NEPAL	2005Q2	-2.1291	0.1667	0.6443	3.9419	4.1803	6.0000	70.6500
NEPAL	2005Q3	-2.5062	0.1667	0.6443	7.0593	4.1802	6.0000	71.3000
NEPAL	2005Q4	-2.9694	0.1667	0.6443	8.5314	3.4792	6.0000	74.0500
NEPAL	2006Q1	-2.8159	0.1667	0.6443	4.9073	4.1802	6.2500	71.9450
NEPAL	2006Q2	-2.7396	0.1667	0.6443	7.9919	4.1803	6.2500	74.4000
NEPAL	2006Q3	-2.7333	0.1667	0.6443	2.5763	4.1802	6.2500	73.6000
NEPAL	2006Q4	-2.7882	0.1667	0.6443	0.0000	3.3646	6.2500	71.1000
NEPAL	2007Q1	-2.9676	0.1667	0.6443	0.0000	4.1802	6.2500	69.8500
NEPAL	2007Q2	-3.1853	0.1667	0.6443	0.0000	4.1803	6.2500	65.4000
NEPAL	2007Q3	-3.4274	0.1668	0.6443	3.8298	4.1802	6.2500	64.0000
NEPAL	2007Q4	-3.6776	0.1670	0.6443	4.9996	3.4116	6.2500	63.5500
NEPAL	2008Q1	-1.3574	0.1676	0.6443	5.2177	4.1802	6.5000	64.0000
NEPAL	2008Q2	0.9934	0.1693	0.6443	8.3990	4.1803	6.5000	68.6000
NEPAL	2008Q3	3.3963	0.1743	0.6443	11.0665	4.1806	6.5000	75.3500
NEPAL	2008Q4	5.8721	0.1883	0.6443	12.6441	6.1046	6.5000	77.6500
NEPAL	2009Q1	6.7281	0.2283	0.6443	12.4527	4.1806	6.5000	82.5500
NEPAL	2009Q2	7.6907	0.2424	0.6443	11.3332	4.1803	6.5000	75.6000
NEPAL	2009Q3	8.7705	0.2473	0.6443	9.6288	4.1804	6.5000	77.5000
NEPAL	2009Q4	9.9737	0.2491	0.6443	8.9395	4.5331	6.5000	74.4400
NEPAL	2010Q1	8.2721	0.2497	0.6443	10.0222	4.1804	7.0000	72.4000
NEPAL	2010Q2	6.6887	0.2499	0.6443	8.5766	4.1803	7.0000	74.6000
NEPAL	2010Q3	5.2123	0.2500	0.6443	8.6481	4.1804	7.0000	73.9000
NEPAL	2010Q4	3.8281	0.2500	0.6443	8.4952	4.8164	7.0000	71.6500
NEPAL	2011Q1	1.5978	0.2500	0.6443	10.1176	4.1804	7.0000	72.4000
NEPAL	2011Q2	-0.5795	0.2500	0.6443	9.1124	4.1803	7.0000	71.7900
NEPAL	2011Q3	-2.7255	0.2500	0.6443	8.2148	4.1802	7.0000	75.6000
NEPAL	2011Q4	-4.8616	0.2500	0.6443	7.9652	3.4218	7.0000	85.5100
NEPAL	2012Q1	-4.3323	0.2500	0.6443	6.7271	4.1802	8.0000	79.5500
NEPAL	2012Q2	-3.8293	0.2500	0.6443	8.3721	4.1803	8.0000	88.6000
NEPAL	2012Q3	-3.3666	0.2500	0.6443	10.9064	4.1804	8.0000	87.5300
NEPAL	2012Q4	-2.9557	0.2500	0.6443	9.9387	4.7812	8.0000	86.9100
NEPAL	2013Q1	-2.7871	0.2500	0.6443	9.5703	4.1804	8.0000	87.1600
NEPAL	2013Q2	-2.6873	0.2500	0.6443	8.4219	4.1803	8.0000	93.0200
NEPAL	2013Q3	-2.6622	0.2500	0.6443	7.5596	4.1803	8.0000	101.5900
NEPAL	2013Q4	-2.7158	0.2500	0.6443	9.1192	4.1289	8.0000	99.4100
NEPAL	2014Q1	-2.2765	0.2500	0.6443	8.7152	4.1803	8.0000	97.8800
NEPAL	2014Q2	-1.9192	0.2500	0.6443	9.1246	4.1803	8.0000	95.6200
NEPAL	2014Q3	-1.6434	0.2501	0.6443	7.4252	4.1806	8.0000	97.8200
NEPAL	2014Q4	-1.4471	0.2503	0.6443	6.9869	5.9890	8.0000	99.6700
NEPAL	2015Q1	-1.7185	0.2509	0.6443	6.7277	4.1806	7.0000	100.7500
NEPAL	2015Q2	-2.0628	0.2527	0.6443	6.8645	4.1803	7.0000	102.5000
NEPAL	2015Q3	-2.4753	0.2576	0.6443	6.9694	4.1802	7.0000	106.2200
NEPAL	2015Q4	-2.9497	0.2717	0.6443	9.5913	3.3229	7.0000	107.3000
NEPAL	2016Q1	-0.8557	0.3117	0.6443	10.6089	4.1802	7.0000	107.5300
NEPAL	2016Q2	1.1935	0.3257	0.6443	9.7996	4.1803	7.0000	107.3800
NEPAL	2016Q3	3.2080	0.3307	0.6443	8.5828	4.1797	7.0000	107.1500
NEPAL	2016Q4	5.1970	0.3324	0.6443	4.9670	0.4129	7.0000	108.0000
NEPAL	2017Q1	3.6138	0.3330	0.6443	3.0698	4.1797	7.0000	106.5700
NEPAL	2017Q2	2.0165	0.3332	0.6443	3.2444	4.1803	7.0000	103.0700
NEPAL	2017Q3	0.4069	0.3333	0.6443	2.7547	4.1808	7.0000	102.5200
NEPAL	2017Q4	-1.2145	0.3333	0.6443	3.6344	7.4994	7.0000	102.9500
NEPAL	2018Q1	-0.5846	0.3346	0.6443	7.2850	8.6386	1.6250	105.6164
NEPAL	2018Q2	0.0349	0.3367	0.6443	6.3086	8.2998	1.6250	106.7210
NEPAL	2018Q3	0.6453	0.3393	0.6443	5.3322	7.9611	1.6250	107.8256

NEPAL	2018Q4	1.2481	0.3421	0.6443	4.3559	7.6224	1.6250	108.9301
NEPAL	2019Q1	0.5576	0.3451	0.6443	4.4397	7.3810	1.5000	109.8500
NEPAL	2019Q2	-0.1394	0.3482	0.6443	4.5235	7.1397	1.5000	110.7698
NEPAL	2019Q3	-0.8430	0.3514	0.6443	4.6073	6.8984	1.5000	111.6896
NEPAL	2019Q4	-1.5533	0.3547	0.6443	4.6912	6.6571	1.5000	112.6095
NEPAL	2020Q1	-0.6985	0.3580	0.6443	4.4206	4.4707	1.2500	114.0434
NEPAL	2020Q2	0.1516	0.3614	0.6443	4.1501	2.2843	1.2500	115.4773
NEPAL	2020Q3	0.9987	0.3648	0.6443	3.8795	0.0980	1.2500	116.9113
NEPAL	2020Q4	1.8446	0.3682	0.6443	3.6090	-2.0884	1.2500	118.3452
NEPAL	2021Q1	-0.0224	0.3510	0.6443	3.0300	5.8000	5.0000	116.7300
NEPAL	2021Q2	-0.0225	0.3537	0.6443	4.1900	0.8000	5.0000	118.9400
NEPAL	2021Q3	-0.0225	0.3564	0.6443	3.4900	5.8000	5.0000	118.5600
NEPAL	2021Q4	-0.0225	0.3591	0.6443	7.1100	5.8000	5.0000	118.9400
NICARAGUA	2000Q1	3.2826	0.0000	0.7218	6.5097	5.9810	8.7500	12.4980
NICARAGUA	2000Q2	3.6916	0.0000	0.7218	7.6518	5.2150	8.7500	12.6804
NICARAGUA	2000Q3	4.0975	0.0000	0.7218	6.9010	4.6192	8.7500	12.8675
NICARAGUA	2000Q4	4.4960	0.0000	0.7218	6.2746	4.1016	8.7500	13.0573
NICARAGUA	2001Q1	2.3317	0.0000	0.7218	6.0543	3.9038	8.5000	13.2463
NICARAGUA	2001Q2	0.1425	0.0000	0.7218	6.2518	3.6733	8.5000	13.4401
NICARAGUA	2001Q3	-2.0838	0.0000	0.7218	5.9015	3.3744	8.5000	13.6390
NICARAGUA	2001Q4	-4.3120	0.0000	0.7218	5.0632	2.9608	8.5000	13.8408
NICARAGUA	2002Q1	-3.8240	0.0000	0.7218	3.9150	2.7867	6.2500	14.0411
NICARAGUA	2002Q2	-3.3988	0.0000	0.7218	3.4941	2.4069	6.2500	14.2466
NICARAGUA	2002Q3	-3.0412	0.0000	0.7218	3.7077	1.7626	6.2500	14.4573
NICARAGUA	2002Q4	-2.7541	0.0000	0.7218	3.6138	0.7539	6.2500	14.6712
NICARAGUA	2003Q1	-2.2540	0.0000	0.7218	4.8984	1.6853	4.2500	14.8835
NICARAGUA	2003Q2	-1.8244	0.0000	0.7218	4.7974	2.2404	4.2500	15.1013
NICARAGUA	2003Q3	-1.4629	0.0000	0.7218	5.0796	2.5052	4.2500	15.3248
NICARAGUA	2003Q4	-1.1662	0.0000	0.7218	5.8759	2.5207	4.2500	15.5515
NICARAGUA	2004Q1	-1.0774	0.0000	0.7218	6.8592	3.3054	3.5000	15.7413
NICARAGUA	2004Q2	-1.0440	0.0000	0.7218	7.8257	3.9648	3.5000	15.9334
NICARAGUA	2004Q3	-1.0603	0.0000	0.7218	8.3606	4.6009	3.5000	16.1300
NICARAGUA	2004Q4	-1.1199	0.0000	0.7218	9.4122	5.3122	3.5000	16.3291
NICARAGUA	2005Q1	-0.9606	0.0000	0.7218	8.0234	4.9101	3.5000	16.5267
NICARAGUA	2005Q2	-0.8299	0.0000	0.7218	9.2915	4.6312	3.5000	16.7290
NICARAGUA	2005Q3	-0.7193	0.0000	0.7218	10.0720	4.4323	3.5000	16.9360
NICARAGUA	2005Q4	-0.6200	0.0000	0.7218	9.2249	4.2824	3.5000	17.1455
NICARAGUA	2006Q1	-0.3072	0.0000	0.7218	9.8024	4.1676	3.2500	17.3531
NICARAGUA	2006Q2	0.0133	0.0000	0.7218	9.3034	4.0610	3.2500	17.5654
NICARAGUA	2006Q3	0.3514	0.0000	0.7218	8.0945	3.9460	3.2500	17.7828
NICARAGUA	2006Q4	0.7169	0.0000	0.7218	7.8571	3.8049	3.2500	18.0028
NICARAGUA	2007Q1	1.7842	0.0000	0.7218	8.6783	4.0854	3.0000	18.2207
NICARAGUA	2007Q2	2.8977	0.0000	0.7218	8.6400	4.3614	3.0000	18.4437
NICARAGUA	2007Q3	4.0657	0.0000	0.7218	9.8861	4.6755	3.0000	18.6719
NICARAGUA	2007Q4	5.2944	0.0000	0.7218	14.7227	5.0764	3.0000	18.9030
NICARAGUA	2008Q1	4.5691	0.0000	0.7218	16.7107	4.6107	3.0000	19.1337
NICARAGUA	2008Q2	3.9088	0.0000	0.7218	19.3974	4.2217	3.0000	19.3672
NICARAGUA	2008Q3	3.3109	0.0000	0.7218	21.0592	3.8493	3.0000	19.6062
NICARAGUA	2008Q4	2.7705	0.0000	0.7218	15.2161	3.4357	3.0000	19.8481
NICARAGUA	2009Q1	1.9425	0.0000	0.7218	10.0215	2.3800	3.0000	20.0883
NICARAGUA	2009Q2	1.1564	0.0000	0.7218	4.1815	1.0557	3.0000	20.3342
NICARAGUA	2009Q3	0.4024	0.0000	0.7218	-0.0083	-0.7424	3.0000	20.5858
NICARAGUA	2009Q4	-0.3301	0.0000	0.7218	0.6027	-3.2927	3.0000	20.8405
NICARAGUA	2010Q1	-0.7389	0.0000	0.6910	2.9624	-0.5713	2.7500	21.0927
NICARAGUA	2010Q2	-1.1476	0.0000	0.6910	4.6117	1.4243	2.7500	21.3509
NICARAGUA	2010Q3	-1.5665	0.0000	0.6910	5.4979	3.0033	2.7500	21.6151
NICARAGUA	2010Q4	-2.0051	0.0000	0.6910	8.0916	4.4101	2.7500	21.8825
NICARAGUA	2011Q1	-2.2004	0.0000	0.6910	7.0674	4.6903	2.0000	22.1474
NICARAGUA	2011Q2	-2.4311	0.0000	0.6910	7.8250	5.0596	2.0000	22.4184
NICARAGUA	2011Q3	-2.7030	0.0000	0.6910	8.8134	5.5751	2.0000	22.6958
NICARAGUA	2011Q4	-3.0206	0.0000	0.6910	7.3745	6.3167	2.0000	22.9767

NICARAGUA	2012Q1	-2.6084	0.0000	0.6910	8.1887	5.9413	1.2500	23.2571
NICARAGUA	2012Q2	-2.2452	0.0000	0.6910	7.3712	5.8487	1.2500	23.5409
NICARAGUA	2012Q3	-1.9301	0.0000	0.6910	6.0605	6.0246	1.2500	23.8314
NICARAGUA	2012Q4	-1.6609	0.0000	0.6910	6.2199	6.4961	1.2500	24.1255
NICARAGUA	2013Q1	-1.2784	0.0000	0.6910	6.9575	5.7806	1.2500	24.4175
NICARAGUA	2013Q2	-0.9337	0.0000	0.6910	7.1843	5.3230	1.2500	24.7163
NICARAGUA	2013Q3	-0.6216	0.0000	0.6910	7.3698	5.0525	1.2500	25.0222
NICARAGUA	2013Q4	-0.3362	0.0000	0.6910	6.0731	4.9271	1.2500	25.3318
NICARAGUA	2014Q1	-0.3604	0.0000	0.6910	5.0529	4.7522	1.2500	25.6384
NICARAGUA	2014Q2	-0.3985	0.0000	0.6910	5.1343	4.6759	1.2500	25.9521
NICARAGUA	2014Q3	-0.4438	0.0001	0.6910	6.5041	4.6864	1.2500	26.2733
NICARAGUA	2014Q4	-0.4892	0.0003	0.6910	6.7120	4.7855	1.2500	26.5984
NICARAGUA	2015Q1	-0.2774	0.0009	0.6910	5.4751	4.6586	1.2500	26.9203
NICARAGUA	2015Q2	-0.0509	0.0027	0.6910	4.7820	4.6159	1.2500	27.2497
NICARAGUA	2015Q3	0.1978	0.0076	0.6910	2.8245	4.6508	1.2500	27.5869
NICARAGUA	2015Q4	0.4767	0.0217	0.6910	2.6523	4.7686	1.2500	27.9283
NICARAGUA	2016Q1	0.4779	0.0617	0.6910	3.3610	4.6285	1.2500	28.2691
NICARAGUA	2016Q2	0.5242	0.0757	0.6910	3.5400	4.5680	1.2500	28.6142
NICARAGUA	2016Q3	0.6227	0.0807	0.6910	3.7254	4.5776	1.2500	28.9672
NICARAGUA	2016Q4	0.7799	0.0824	0.6910	3.2242	4.6588	1.2500	29.3247
NICARAGUA	2017Q1	1.6036	0.0830	0.6910	3.5151	4.5938	1.2500	29.6796
NICARAGUA	2017Q2	2.4982	0.0832	0.6910	3.0600	4.6030	1.2500	30.0428
NICARAGUA	2017Q3	3.4686	0.0833	0.6910	3.5106	4.6877	1.2500	30.4146
NICARAGUA	2017Q4	4.5181	0.0833	0.6910	5.0075	4.8611	1.2500	30.7909
NICARAGUA	2018Q1	3.9000	0.0845	0.6910	3.7573	2.6326	1.0000	30.4265
NICARAGUA	2018Q2	3.3603	0.0854	0.6910	3.3910	0.6338	1.0000	30.8021
NICARAGUA	2018Q3	2.8949	0.0864	0.6910	3.0248	-1.3649	1.0000	31.1776
NICARAGUA	2018Q4	2.4975	0.0875	0.6910	2.6585	-3.3637	1.0000	31.5532
NICARAGUA	2019Q1	1.1649	0.0886	0.6910	3.3717	-3.4413	1.0000	31.9453
NICARAGUA	2019Q2	-0.1174	0.0897	0.6910	4.0849	-3.5190	1.0000	32.3375
NICARAGUA	2019Q3	-1.3597	0.0908	0.6910	4.7981	-3.5966	1.0000	32.7296
NICARAGUA	2019Q4	-2.5723	0.0919	0.6910	5.5114	-3.6743	1.0000	33.1217
NICARAGUA	2020Q1	-2.8054	0.0931	0.6910	5.5991	-3.2500	1.0000	33.4268
NICARAGUA	2020Q2	-3.0260	0.0942	0.6910	5.6868	-2.8258	1.0000	33.7319
NICARAGUA	2020Q3	-3.2403	0.0954	0.6910	5.7745	-2.4016	1.0000	34.0370
NICARAGUA	2020Q4	-3.4524	0.0965	0.6910	5.8622	-1.9773	1.0000	34.3421
NICARAGUA	2021Q1	0.0000	0.0694	0.6910	4.1100	4.2000	1.0000	34.8080
NICARAGUA	2021Q2	0.0000	0.0706	0.6910	3.9400	17.7000	1.0000	34.8460
NICARAGUA	2021Q3	0.0000	0.0717	0.6910	5.8600	10.2000	1.0000	35.0770
NICARAGUA	2021Q4	0.0000	0.0729	0.6910	7.2100	10.1000	1.0000	35.2690
MOROCCO	2000Q1	3.2868	0.0000	0.1439	0.2416	1.2892	1.2500	10.0097
MOROCCO	2000Q2	3.8017	0.0000	0.1439	-0.0864	1.4971	1.2500	10.2150
MOROCCO	2000Q3	4.4093	0.0000	0.1439	-0.4143	1.7050	1.2500	10.4203
MOROCCO	2000Q4	5.1047	0.0000	0.1439	-0.7423	1.9129	1.2500	10.6256
MOROCCO	2001Q1	3.5427	0.0000	0.1439	-0.3936	3.2646	1.1775	10.7950
MOROCCO	2001Q2	2.0495	0.0000	0.1439	-0.0450	4.6164	1.1775	10.9643
MOROCCO	2001Q3	0.6120	0.0000	0.1439	0.3037	5.9682	1.1775	11.1336
MOROCCO	2001Q4	-0.7843	0.0000	0.1439	0.6523	7.3200	1.1775	11.3030
MOROCCO	2002Q1	-0.9297	0.0000	0.1439	0.7914	6.2703	0.9475	11.2324
MOROCCO	2002Q2	-1.0631	0.0000	0.1439	0.9304	5.2207	0.9475	11.1618
MOROCCO	2002Q3	-1.1985	0.0000	0.1439	1.0695	4.1711	0.9475	11.0912
MOROCCO	2002Q4	-1.3490	0.0000	0.1439	1.2085	3.1214	0.9475	11.0206
MOROCCO	2003Q1	-1.4619	0.0000	0.1439	1.1721	3.8314	0.8125	10.6590
MOROCCO	2003Q2	-1.6138	0.0000	0.1439	1.1356	4.5413	0.8125	10.2975
MOROCCO	2003Q3	-1.8153	0.0000	0.1439	1.0991	5.2512	0.8125	9.9359
MOROCCO	2003Q4	-2.0762	0.0000	0.1439	1.0626	5.9612	0.8125	9.5744
MOROCCO	2004Q1	-2.1729	0.0000	0.1439	1.1018	5.6701	0.8125	9.3978
MOROCCO	2004Q2	-2.3446	0.0000	0.1439	1.1409	5.3791	0.8125	9.2212
MOROCCO	2004Q3	-2.5972	0.0000	0.1439	1.1801	5.0881	0.8125	9.0446
MOROCCO	2004Q4	-2.9350	0.0000	0.1439	1.2192	4.7970	0.8125	8.8680
MOROCCO	2005Q1	-2.5466	0.0000	0.1439	1.2121	4.4207	0.8125	8.8673

MOROCCO	2005Q2	-2.2472	0.0000	0.1439	1.2049	4.0443	0.8125	8.8665
MOROCCO	2005Q3	-2.0359	0.0000	0.1439	1.1977	3.6680	0.8125	8.8658
MOROCCO	2005Q4	-1.9108	0.0000	0.1439	1.1905	3.2916	0.8125	8.8650
MOROCCO	2006Q1	-2.0953	0.0000	0.2709	1.2799	4.3624	0.8125	8.8477
MOROCCO	2006Q2	-2.3581	0.0000	0.3979	1.3693	5.4331	0.8125	8.8303
MOROCCO	2006Q3	-2.6933	0.0001	0.5249	1.4587	6.5039	0.8125	8.8129
MOROCCO	2006Q4	-3.0935	0.0003	0.6519	1.5480	7.5746	0.8125	8.7956
MOROCCO	2007Q1	-1.8993	0.0009	0.6519	2.0598	6.5639	0.8125	8.6448
MOROCCO	2007Q2	-0.7501	0.0027	0.6519	2.5715	5.5531	0.8125	8.4940
MOROCCO	2007Q3	0.3661	0.0076	0.6519	3.0833	4.5424	0.8125	8.3431
MOROCCO	2007Q4	1.4619	0.0217	0.6519	3.5951	3.5316	0.8125	8.1923
MOROCCO	2008Q1	1.5704	0.0617	0.6519	3.8289	4.1295	0.8300	8.0818
MOROCCO	2008Q2	1.6824	0.0757	0.6519	4.0628	4.7274	0.8300	7.9713
MOROCCO	2008Q3	1.8082	0.0807	0.6519	4.2966	5.3254	0.8300	7.8608
MOROCCO	2008Q4	1.9575	0.0824	0.6519	4.5305	5.9233	0.8300	7.7503
MOROCCO	2009Q1	1.5025	0.0830	0.6519	3.4346	5.5034	0.8275	7.8270
MOROCCO	2009Q2	1.0862	0.0833	0.6519	2.3387	5.0835	0.8275	7.9037
MOROCCO	2009Q3	0.7147	0.0834	0.6519	1.2428	4.6636	0.8275	7.9804
MOROCCO	2009Q4	0.3936	0.0836	0.6519	0.1469	4.2438	0.8275	8.0571
MOROCCO	2010Q1	0.3620	0.0843	0.6519	0.3541	4.1367	0.8125	8.1471
MOROCCO	2010Q2	0.3905	0.0860	0.6519	0.5613	4.0297	0.8125	8.2371
MOROCCO	2010Q3	0.4836	0.0909	0.6519	0.7684	3.9227	0.8125	8.3271
MOROCCO	2010Q4	0.6456	0.1050	0.6519	0.9756	3.8157	0.8125	8.4172
MOROCCO	2011Q1	0.8917	0.1450	0.6519	0.5590	4.1732	0.8125	8.3353
MOROCCO	2011Q2	1.2143	0.1591	0.6519	0.1423	4.5307	0.8125	8.2535
MOROCCO	2011Q3	1.6163	0.1640	0.6519	-0.2743	4.8882	0.8125	8.1717
MOROCCO	2011Q4	2.1001	0.1657	0.6519	-0.6910	5.2457	0.8125	8.0899
MOROCCO	2012Q1	2.0759	0.1663	0.6519	-0.4259	4.6868	0.7975	8.2245
MOROCCO	2012Q2	2.1346	0.1666	0.6519	-0.1608	4.1278	0.7975	8.3592
MOROCCO	2012Q3	2.2749	0.1666	0.6519	0.1043	3.5689	0.7975	8.4938
MOROCCO	2012Q4	2.4942	0.1667	0.6519	0.3693	3.0100	0.7975	8.6284
MOROCCO	2013Q1	1.6240	0.1667	0.6519	0.6038	3.3913	0.7650	8.5727
MOROCCO	2013Q2	0.8230	0.1667	0.6519	0.8383	3.7727	0.7650	8.5170
MOROCCO	2013Q3	0.0845	0.1667	0.6519	1.0728	4.1541	0.7650	8.4612
MOROCCO	2013Q4	-0.5988	0.1667	0.6519	1.3073	4.5354	0.7650	8.4055
MOROCCO	2014Q1	-0.3462	0.1667	0.6519	1.0749	4.0689	0.7350	8.4057
MOROCCO	2014Q2	-0.0525	0.1667	0.6519	0.8426	3.6025	0.7350	8.4059
MOROCCO	2014Q3	0.2757	0.1668	0.6519	0.6102	3.1360	0.7350	8.4061
MOROCCO	2014Q4	0.6318	0.1670	0.6519	0.3778	2.6695	0.7350	8.4063
MOROCCO	2015Q1	-0.1437	0.1676	0.6519	0.8156	3.1362	0.6275	8.7458
MOROCCO	2015Q2	-0.9054	0.1694	0.6519	1.2534	3.6029	0.6275	9.0853
MOROCCO	2015Q3	-1.6603	0.1744	0.6519	1.6912	4.0697	0.6275	9.4248
MOROCCO	2015Q4	-2.4149	0.1886	0.6519	2.1290	4.5364	0.6275	9.7643
MOROCCO	2016Q1	-2.0018	0.2293	0.6519	1.9676	3.6672	0.5675	9.7751
MOROCCO	2016Q2	-1.5980	0.2451	0.6519	1.8061	2.7981	0.5675	9.7859
MOROCCO	2016Q3	-1.2062	0.2549	0.6519	1.6446	1.9290	0.5675	9.7967
MOROCCO	2016Q4	-0.8281	0.2707	0.6519	1.4832	1.0599	0.5675	9.8075
MOROCCO	2017Q1	-0.7628	0.3113	0.6519	1.2725	1.8571	0.5675	9.7786
MOROCCO	2017Q2	-0.7127	0.3256	0.6519	1.0618	2.6544	0.5675	9.7497
MOROCCO	2017Q3	-0.6778	0.3305	0.6519	0.8511	3.4517	0.5675	9.7209
MOROCCO	2017Q4	-0.6577	0.3320	0.6519	0.6404	4.2489	0.5675	9.6920
MOROCCO	2018Q1	-0.5620	0.3397	0.6519	0.7527	3.9738	0.5675	9.6155
MOROCCO	2018Q2	-0.4790	0.3449	0.6519	0.8650	3.6987	0.5675	9.5391
MOROCCO	2018Q3	-0.4069	0.3482	0.6519	0.9773	3.4236	0.5675	9.4626
MOROCCO	2018Q4	-0.3438	0.3504	0.6519	1.0896	3.1485	0.5675	9.3861
MOROCCO	2019Q1	0.1371	0.3531	0.6519	1.1517	2.9819	0.5675	9.4438
MOROCCO	2019Q2	0.6135	0.3571	0.6519	1.2137	2.8152	0.5675	9.5016
MOROCCO	2019Q3	1.0879	0.3621	0.6519	1.2757	2.6486	0.5675	9.5593
MOROCCO	2019Q4	1.5621	0.3677	0.6519	1.3377	2.4819	0.5675	9.6171
MOROCCO	2020Q1	0.9812	0.3730	0.6519	1.0657	0.0821	0.3750	9.5870
MOROCCO	2020Q2	0.4017	0.3778	0.6519	0.7937	-2.3177	0.3750	9.5570

MOROCCO	2020Q3	-0.1768	0.3822	0.6519	0.5218	-4.7174	0.3750	9.5269
MOROCCO	2020Q4	-0.7548	0.3864	0.6519	0.2498	-7.1172	0.3750	9.4968
MOROCCO	2021Q1	-0.1075	0.3496	0.6519	0.1000	2.0000	1.5000	9.0625
MOROCCO	2021Q2	-0.1097	0.3548	0.6519	1.5000	14.1000	1.5000	8.9183
MOROCCO	2021Q3	-0.1120	0.3599	0.6519	1.2000	8.7000	1.5000	9.0577
MOROCCO	2021Q4	-0.1142	0.3651	0.6519	3.2000	7.6000	1.5000	10.2550
THAILAND	2000Q1	-4.4573	0.0000	0.1345	-1.6000	4.5430	0.4875	38.3882
THAILAND	2000Q2	-7.0670	0.0000	0.1345	-0.6230	4.5138	0.4875	38.9627
THAILAND	2000Q3	-9.6831	0.0000	0.1345	0.3540	4.4845	0.4875	39.5373
THAILAND	2000Q4	-12.3551	0.0000	0.1345	1.3310	4.4552	0.4875	40.1118
THAILAND	2001Q1	-12.4886	0.0000	0.1345	1.4777	4.2025	0.5000	41.1918
THAILAND	2001Q2	-12.7574	0.0000	0.1345	1.6243	3.9497	0.5000	42.2719
THAILAND	2001Q3	-13.1896	0.0000	0.1345	1.7709	3.6970	0.5000	43.3519
THAILAND	2001Q4	-13.8053	0.0000	0.1345	1.9175	3.4442	0.5000	44.4319
THAILAND	2002Q1	-10.6578	0.0000	0.1345	1.8608	4.1204	0.4400	44.0639
THAILAND	2002Q2	-7.7088	0.0000	0.1345	1.8040	4.7966	0.4400	43.6960
THAILAND	2002Q3	-4.9548	0.0000	0.1345	1.7472	5.4728	0.4400	43.3280
THAILAND	2002Q4	-2.3875	0.0000	0.1345	1.6905	6.1490	0.4400	42.9601
THAILAND	2003Q1	-1.6270	0.0000	0.1345	1.8052	6.4091	0.3275	42.5912
THAILAND	2003Q2	-1.0290	0.0000	0.1345	1.9200	6.6691	0.3275	42.2224
THAILAND	2003Q3	-0.5796	0.0000	0.1345	2.0348	6.9292	0.3275	41.8535
THAILAND	2003Q4	-0.2642	0.0000	0.1345	2.1495	7.1892	0.3275	41.4846
THAILAND	2004Q1	0.8676	0.0000	0.1345	2.5045	6.9643	0.3075	41.1691
THAILAND	2004Q2	1.8953	0.0000	0.1345	2.8594	6.7393	0.3075	40.8535
THAILAND	2004Q3	2.8334	0.0000	0.1345	3.2143	6.5143	0.3075	40.5380
THAILAND	2004Q4	3.6953	0.0000	0.1345	3.5693	6.2893	0.3075	40.2224
THAILAND	2005Q1	3.9121	0.0000	0.1345	3.9498	5.7639	0.6550	40.2218
THAILAND	2005Q2	4.0735	0.0000	0.1345	4.3304	5.2385	0.6550	40.2213
THAILAND	2005Q3	4.1862	0.0000	0.1345	4.7110	4.7131	0.6550	40.2207
THAILAND	2005Q4	4.2546	0.0000	0.1345	5.0916	4.1876	0.6550	40.2201
THAILAND	2006Q1	3.3783	0.0000	0.1345	5.0947	4.3827	1.1600	39.6356
THAILAND	2006Q2	2.4582	0.0000	0.1345	5.0979	4.5777	1.1600	39.0511
THAILAND	2006Q3	1.4912	0.0000	0.1345	5.1010	4.7728	1.1600	38.4665
THAILAND	2006Q4	0.4728	0.0000	0.1345	5.1042	4.9678	1.1600	37.8820
THAILAND	2007Q1	-0.0421	0.0000	0.3815	4.4465	5.0846	0.9375	37.0410
THAILAND	2007Q2	-0.6199	0.0000	0.3815	3.7888	5.2015	0.9375	36.2001
THAILAND	2007Q3	-1.2666	0.0000	0.3815	3.1311	5.3183	0.9375	35.3591
THAILAND	2007Q4	-1.9877	0.0000	0.3815	2.4733	5.4352	0.9375	34.5182
THAILAND	2008Q1	-1.7473	0.0000	0.3815	3.1384	4.5078	0.8200	34.2170
THAILAND	2008Q2	-1.5894	0.0000	0.3815	3.8036	3.5804	0.8200	33.9157
THAILAND	2008Q3	-1.5164	0.0000	0.3815	4.4687	2.6531	0.8200	33.6145
THAILAND	2008Q4	-1.5294	0.0000	0.3815	5.1338	1.7257	0.8200	33.3133
THAILAND	2009Q1	-1.3413	0.0000	0.3815	3.8990	1.1216	0.3025	33.5564
THAILAND	2009Q2	-1.2392	0.0000	0.3815	2.6643	0.5175	0.3025	33.7995
THAILAND	2009Q3	-1.2217	0.0000	0.3815	1.4295	-0.0865	0.3025	34.0427
THAILAND	2009Q4	-1.2867	0.0000	0.3815	0.1948	-0.6906	0.3025	34.2858
THAILAND	2010Q1	-2.0040	0.0000	0.3815	1.1663	1.3604	0.3125	33.6358
THAILAND	2010Q2	-2.7971	0.0001	0.3815	2.1379	3.4114	0.3125	32.9857
THAILAND	2010Q3	-3.6611	0.0002	0.3815	3.1094	5.4624	0.3125	32.3357
THAILAND	2010Q4	-4.5891	0.0007	0.3815	4.0810	7.5134	0.3125	31.6857
THAILAND	2011Q1	-2.9710	0.0019	0.3815	3.9965	5.8451	0.7000	31.3872
THAILAND	2011Q2	-1.3961	0.0053	0.3815	3.9120	4.1768	0.7000	31.0887
THAILAND	2011Q3	0.1494	0.0152	0.3815	3.8276	2.5084	0.7000	30.7902
THAILAND	2011Q4	1.6800	0.0433	0.3815	3.7431	0.8401	0.7000	30.4917
THAILAND	2012Q1	1.7596	0.1234	0.3815	3.2846	2.4408	0.7225	30.6396
THAILAND	2012Q2	1.8524	0.1515	0.3815	2.8261	4.0415	0.7225	30.7874
THAILAND	2012Q3	1.9707	0.1613	0.3815	2.3676	5.6421	0.7225	30.9353
THAILAND	2012Q4	2.1258	0.1648	0.3815	1.9091	7.2428	0.7225	31.0831
THAILAND	2013Q1	2.3786	0.1660	0.3815	1.8765	6.1040	0.6350	30.9938
THAILAND	2013Q2	2.6869	0.1664	0.3815	1.8439	4.9651	0.6350	30.9045
THAILAND	2013Q3	3.0577	0.1666	0.3815	1.8113	3.8263	0.6350	30.8152

THAILAND	2013Q4	3.4966	0.1666	0.3815	1.7787	2.6875	0.6350	30.7260
THAILAND	2014Q1	3.3387	0.1667	0.3815	1.6944	2.2617	0.5000	31.1644
THAILAND	2014Q2	3.2539	0.1667	0.3815	1.6101	1.8360	0.5000	31.6029
THAILAND	2014Q3	3.2414	0.1667	0.3815	1.5258	1.4102	0.5000	32.0414
THAILAND	2014Q4	3.2985	0.1667	0.3815	1.4415	0.9845	0.5000	32.4798
THAILAND	2015Q1	3.2674	0.1667	0.3815	1.2616	1.5219	0.3975	32.9218
THAILAND	2015Q2	3.2944	0.1667	0.3815	1.0818	2.0593	0.3975	33.3638
THAILAND	2015Q3	3.3705	0.1667	0.3815	0.9020	2.5967	0.3975	33.8057
THAILAND	2015Q4	3.4849	0.1667	0.3815	0.7221	3.1340	0.3975	34.2477
THAILAND	2016Q1	2.6258	0.1667	0.3815	1.2006	3.2093	0.3625	34.5099
THAILAND	2016Q2	1.7767	0.1667	0.3815	1.6791	3.2846	0.3625	34.7721
THAILAND	2016Q3	0.9208	0.1667	0.3815	2.1577	3.3599	0.3625	35.0342
THAILAND	2016Q4	0.0402	0.1667	0.3815	2.6362	3.4352	0.3625	35.2964
THAILAND	2017Q1	-0.7565	0.1667	0.3815	2.4521	3.6208	0.3750	34.9572
THAILAND	2017Q2	-1.6150	0.1667	0.3815	2.2681	3.8064	0.3750	34.6181
THAILAND	2017Q3	-2.5535	0.1667	0.3815	2.0840	3.9921	0.3750	34.2790
THAILAND	2017Q4	-3.5890	0.1667	0.3815	1.8999	4.1777	0.3750	33.9398
THAILAND	2018Q1	-4.3079	0.1676	0.3815	1.7830	4.1807	0.4375	33.5324
THAILAND	2018Q2	-5.1526	0.1690	0.3815	1.6661	4.1836	0.4375	33.1250
THAILAND	2018Q3	-6.1335	0.1708	0.3815	1.5492	4.1866	0.4375	32.7176
THAILAND	2018Q4	-7.2580	0.1728	0.3815	1.4322	4.1896	0.4375	32.3102
THAILAND	2019Q1	-8.7565	0.1748	0.3815	1.3107	3.7088	0.3125	31.9946
THAILAND	2019Q2	-10.4010	0.1770	0.3815	1.1892	3.2280	0.3125	31.6789
THAILAND	2019Q3	-12.1850	0.1792	0.3815	1.0677	2.7472	0.3125	31.3633
THAILAND	2019Q4	-14.0954	0.1814	0.3815	0.9462	2.2664	0.3125	31.0476
THAILAND	2020Q1	-3.6845	0.1836	0.3815	0.4475	0.1780	0.1250	31.1091
THAILAND	2020Q2	6.6501	0.1859	0.3815	-0.0513	-1.9104	0.1250	31.1706
THAILAND	2020Q3	16.9401	0.1881	0.3815	-0.5501	-3.9988	0.1250	31.2322
THAILAND	2020Q4	27.2131	0.1904	0.3815	-1.0488	-6.0872	0.1250	31.2937
THAILAND	2021Q1	2.0989	0.2015	0.3815	-0.0800	-2.4000	0.5000	31.2210
THAILAND	2021Q2	2.1738	0.2045	0.3815	1.2500	7.8000	0.5000	32.0670
THAILAND	2021Q3	2.2487	0.2076	0.3815	1.6800	-0.2000	0.5000	33.9450
THAILAND	2021Q4	2.3235	0.2106	0.3815	2.1700	2.0000	0.5000	33.1780
SOUTH_AFRICA	2000Q1	6.7320	0.0000	0.3487	2.7513	3.0191	12.0000	6.5615
SOUTH_AFRICA	2000Q2	6.1491	0.0000	0.3487	4.8103	3.1284	12.0000	6.7715
SOUTH_AFRICA	2000Q3	5.6433	0.0000	0.3487	6.3902	3.2545	12.0000	7.2565
SOUTH_AFRICA	2000Q4	5.2139	0.0000	0.3487	6.7783	3.4029	12.0000	7.5685
SOUTH_AFRICA	2001Q1	7.1610	0.0000	0.3487	7.1577	3.0138	9.5000	8.0145
SOUTH_AFRICA	2001Q2	9.1732	0.0000	0.3487	6.2104	2.6362	9.5000	8.0645
SOUTH_AFRICA	2001Q3	11.2385	0.0000	0.3487	4.6640	2.2535	9.5000	9.0015
SOUTH_AFRICA	2001Q4	13.3394	0.0000	0.3487	4.2138	1.8484	9.5000	12.1265
SOUTH_AFRICA	2002Q1	6.3129	0.0000	0.3487	5.6084	2.3129	13.5000	11.3865
SOUTH_AFRICA	2002Q2	-0.7355	0.0000	0.3487	7.5406	2.7577	13.5000	10.2515
SOUTH_AFRICA	2002Q3	-7.8427	0.0000	0.3487	10.1809	3.2027	13.5000	10.5385
SOUTH_AFRICA	2002Q4	-15.0449	0.0000	0.3487	12.7244	3.6678	13.5000	8.6400
SOUTH_AFRICA	2003Q1	-14.0693	0.0000	0.3487	10.7984	3.4496	8.0000	7.9200
SOUTH_AFRICA	2003Q2	-13.2424	0.0000	0.3487	7.9595	3.2625	8.0000	7.5550
SOUTH_AFRICA	2003Q3	-12.5776	0.0000	0.3487	4.2913	3.0981	8.0000	6.9250
SOUTH_AFRICA	2003Q4	-12.0797	0.0000	0.3487	-0.7312	2.9491	8.0000	6.6400
SOUTH_AFRICA	2004Q1	-10.2646	0.0000	0.3487	-1.7771	3.2970	7.5000	6.3700
SOUTH_AFRICA	2004Q2	-8.6033	0.0000	0.3487	-1.7349	3.6692	7.5000	6.2700
SOUTH_AFRICA	2004Q3	-7.0790	0.0000	0.3487	-0.8863	4.0822	7.5000	6.4500
SOUTH_AFRICA	2004Q4	-5.6696	0.0000	0.3487	1.6368	4.5546	7.5000	5.6300
SOUTH_AFRICA	2005Q1	-4.3092	0.0000	0.3487	1.9150	4.6044	7.0000	6.2350
SOUTH_AFRICA	2005Q2	-3.0070	0.0000	0.3487	1.8439	4.7369	7.0000	6.6500
SOUTH_AFRICA	2005Q3	-1.7300	0.0000	0.3487	2.3676	4.9578	7.0000	6.3600
SOUTH_AFRICA	2005Q4	-0.4437	0.0000	0.3487	2.0390	5.2771	7.0000	6.3250
SOUTH_AFRICA	2006Q1	2.7853	0.0000	0.3487	2.0261	5.1931	9.0000	6.2050
SOUTH_AFRICA	2006Q2	6.0956	0.0000	0.3487	2.5091	5.2180	9.0000	7.1700
SOUTH_AFRICA	2006Q3	9.5215	0.0001	0.3487	3.6931	5.3528	9.0000	7.7650
SOUTH_AFRICA	2006Q4	13.0935	0.0003	0.3487	4.5013	5.6037	9.0000	6.9700

SOUTH_AFRICA	2007Q1	12.9192	0.0009	0.3487	5.0117	5.3715	11.0000	7.2750
SOUTH_AFRICA	2007Q2	12.9320	0.0027	0.3487	5.8399	5.2562	11.0000	7.1100
SOUTH_AFRICA	2007Q3	13.1402	0.0076	0.3487	6.0929	5.2526	11.0000	6.8700
SOUTH_AFRICA	2007Q4	13.5441	0.0217	0.3487	6.9831	5.3605	11.0000	6.8100
SOUTH_AFRICA	2008Q1	8.4047	0.0617	0.3487	8.5740	4.7114	11.5000	8.1000
SOUTH_AFRICA	2008Q2	3.4366	0.0757	0.3487	9.4798	4.1498	11.5000	7.8150
SOUTH_AFRICA	2008Q3	-1.3820	0.0807	0.3487	10.6452	3.6504	11.5000	8.2700
SOUTH_AFRICA	2008Q4	-6.0750	0.0824	0.3487	9.5749	3.1911	11.5000	9.3050
SOUTH_AFRICA	2009Q1	-4.3237	0.0830	0.3487	8.4765	2.1030	7.0000	9.4950
SOUTH_AFRICA	2009Q2	-2.4889	0.0832	0.3487	7.8149	0.9860	7.0000	7.6850
SOUTH_AFRICA	2009Q3	-0.5871	0.0833	0.3487	6.1720	-0.2099	7.0000	7.4400
SOUTH_AFRICA	2009Q4	1.3668	0.0833	0.3487	5.6845	-1.5381	7.0000	7.3800
SOUTH_AFRICA	2010Q1	0.5501	0.0833	0.3652	5.2126	-0.2439	5.5000	7.3350
SOUTH_AFRICA	2010Q2	-0.2445	0.0833	0.3652	4.1178	0.9166	5.5000	7.6400
SOUTH_AFRICA	2010Q3	-1.0327	0.0833	0.3652	3.3025	1.9951	5.5000	6.9800
SOUTH_AFRICA	2010Q4	-1.8302	0.0833	0.3652	3.3368	3.0398	5.5000	6.6316
SOUTH_AFRICA	2011Q1	-3.3250	0.0833	0.3652	3.6693	3.0768	5.5000	6.7925
SOUTH_AFRICA	2011Q2	-4.8577	0.0834	0.3652	4.5462	3.1282	5.5000	6.8164
SOUTH_AFRICA	2011Q3	-6.4403	0.0834	0.3652	5.2728	3.1963	5.5000	8.0828
SOUTH_AFRICA	2011Q4	-8.0813	0.0837	0.3652	6.0487	3.2842	5.5000	8.1429
SOUTH_AFRICA	2012Q1	-6.2048	0.0843	0.3652	6.0054	3.0109	5.0000	7.6733
SOUTH_AFRICA	2012Q2	-4.3912	0.0860	0.3652	5.6856	2.7492	5.0000	8.2028
SOUTH_AFRICA	2012Q3	-2.6360	0.0909	0.3652	5.0511	2.4872	5.0000	8.3052
SOUTH_AFRICA	2012Q4	-0.9324	0.1050	0.3652	5.5367	2.2134	5.0000	8.5012
SOUTH_AFRICA	2013Q1	-0.4335	0.1450	0.3652	5.6651	2.3080	5.0000	9.1979
SOUTH_AFRICA	2013Q2	0.0318	0.1591	0.3652	5.4608	2.3827	5.0000	10.0892
SOUTH_AFRICA	2013Q3	0.4728	0.1640	0.3652	6.0949	2.4409	5.0000	10.0488
SOUTH_AFRICA	2013Q4	0.8992	0.1657	0.3652	5.2462	2.4852	5.0000	10.4899
SOUTH_AFRICA	2014Q1	1.2949	0.1664	0.3652	5.7113	2.3588	5.7500	10.5792
SOUTH_AFRICA	2014Q2	1.6936	0.1666	0.3652	6.4031	2.2147	5.7500	10.6025
SOUTH_AFRICA	2014Q3	2.1033	0.1667	0.3652	6.1588	2.0466	5.7500	11.3347
SOUTH_AFRICA	2014Q4	2.5306	0.1670	0.3652	5.5484	1.8470	5.7500	11.5810
SOUTH_AFRICA	2015Q1	2.0683	0.1676	0.3652	4.1070	1.7762	6.2500	12.1768
SOUTH_AFRICA	2015Q2	1.6329	0.1693	0.3652	4.3922	1.6618	6.2500	12.1983
SOUTH_AFRICA	2015Q3	1.2270	0.1743	0.3652	4.4452	1.4987	6.2500	13.8790
SOUTH_AFRICA	2015Q4	0.8521	0.1883	0.3652	4.6861	1.2795	6.2500	15.5450
SOUTH_AFRICA	2016Q1	0.4215	0.2283	0.3652	6.3513	1.2112	7.0000	14.7321
SOUTH_AFRICA	2016Q2	0.0229	0.2424	0.3652	6.3149	1.0740	7.0000	14.7752
SOUTH_AFRICA	2016Q3	-0.3437	0.2473	0.3652	6.1998	0.8619	7.0000	13.8876
SOUTH_AFRICA	2016Q4	-0.6783	0.2491	0.3652	6.6749	0.5654	7.0000	13.6845
SOUTH_AFRICA	2017Q1	0.3935	0.2497	0.3652	6.2679	0.8710	6.7500	13.2736
SOUTH_AFRICA	2017Q2	1.4980	0.2499	0.3652	5.0661	1.0926	6.7500	13.0625
SOUTH_AFRICA	2017Q3	2.6356	0.2500	0.3652	4.4907	1.2401	6.7500	13.4991
SOUTH_AFRICA	2017Q4	3.8059	0.2500	0.3652	4.4175	1.3201	6.7500	12.3350
SOUTH_AFRICA	2018Q1	2.1349	0.2518	0.3652	4.9297	1.2576	1.6875	13.3013
SOUTH_AFRICA	2018Q2	0.4896	0.2547	0.3652	4.5920	1.1008	1.6875	13.2789
SOUTH_AFRICA	2018Q3	-1.1359	0.2581	0.3652	4.2543	0.9439	1.6875	13.2564
SOUTH_AFRICA	2018Q4	-2.7478	0.2616	0.3652	3.9165	0.7871	1.6875	13.2339
SOUTH_AFRICA	2019Q1	-2.2627	0.2651	0.3652	3.9427	0.6284	1.6250	13.5376
SOUTH_AFRICA	2019Q2	-1.7734	0.2686	0.3652	3.9689	0.4698	1.6250	13.8412
SOUTH_AFRICA	2019Q3	-1.2823	0.2721	0.3652	3.9951	0.3112	1.6250	14.1448
SOUTH_AFRICA	2019Q4	-0.7907	0.2755	0.3652	4.0212	0.1526	1.6250	14.4484
SOUTH_AFRICA	2020Q1	-0.3323	0.2790	0.3652	4.3375	-1.6255	0.8750	14.9511
SOUTH_AFRICA	2020Q2	0.1353	0.2825	0.3652	4.6537	-3.4035	0.8750	15.4538
SOUTH_AFRICA	2020Q3	0.4487	0.2859	0.3652	4.9699	-5.1816	0.8750	15.9564
SOUTH_AFRICA	2020Q4	0.3328	0.2894	0.3652	5.2862	-6.9596	0.8750	16.4591
SOUTH_AFRICA	2021Q1	-0.3587	0.2791	0.3652	3.2000	-2.4000	3.5000	14.7787
SOUTH_AFRICA	2021Q2	-0.3707	0.2832	0.3652	4.9000	19.6000	3.5000	14.7787
SOUTH_AFRICA	2021Q3	-0.3827	0.2873	0.3652	5.0000	3.0000	3.5000	14.7787
SOUTH_AFRICA	2021Q4	-0.3946	0.2913	0.3652	5.8000	1.7000	3.5000	14.7787
SRILANKA	2000Q1	-0.9313	0.0833	0.5755	2.2071	4.8031	16.0000	73.6710

SRILANKA	2000Q2	-1.1568	0.0833	0.5755	4.1933	5.2107	16.0000	78.9720
SRILANKA	2000Q3	-1.3754	0.0833	0.5755	9.0675	5.5892	16.0000	79.4010
SRILANKA	2000Q4	-1.5905	0.0833	0.5755	8.3789	6.0000	16.0000	82.5800
SRILANKA	2001Q1	-1.2246	0.0833	0.5755	14.2575	4.5784	25.0000	86.7923
SRILANKA	2001Q2	-0.8594	0.0833	0.5755	13.9206	3.0252	25.0000	91.5277
SRILANKA	2001Q3	-0.4961	0.0833	0.5755	12.4333	1.0886	25.0000	90.1358
SRILANKA	2001Q4	-0.1348	0.0833	0.5755	12.4249	-1.5454	25.0000	93.1587
SRILANKA	2002Q1	-0.1748	0.0833	0.6055	9.1673	0.7367	18.0000	95.6416
SRILANKA	2002Q2	-0.2168	0.0833	0.6055	9.2327	2.2643	18.0000	96.0965
SRILANKA	2002Q3	-0.2608	0.0833	0.6055	9.0076	3.2851	18.0000	96.2764
SRILANKA	2002Q4	-0.3064	0.0833	0.6055	9.0834	3.9647	18.0000	96.7250
SRILANKA	2003Q1	-0.5009	0.0833	0.6055	10.2770	4.5794	15.0000	96.9457
SRILANKA	2003Q2	-0.6959	0.0833	0.6055	6.0348	5.0628	15.0000	97.1350
SRILANKA	2003Q3	-0.8906	0.0833	0.6055	3.8713	5.4931	15.0000	94.4310
SRILANKA	2003Q4	-1.0834	0.0833	0.6055	4.3680	5.9403	15.0000	96.7382
SRILANKA	2004Q1	-0.7900	0.0833	0.6055	1.2706	5.7491	15.0000	97.4178
SRILANKA	2004Q2	-0.4902	0.0833	0.6055	5.6410	5.6162	15.0000	102.3086
SRILANKA	2004Q3	-0.1809	0.0833	0.6055	9.7949	5.5200	15.0000	103.6519
SRILANKA	2004Q4	0.1413	0.0833	0.6055	12.2373	5.4451	15.0000	104.6050
SRILANKA	2005Q1	0.2422	0.0833	0.6055	13.3673	5.5722	15.0000	99.4111
SRILANKA	2005Q2	0.3630	0.0833	0.6055	11.8308	5.7289	15.0000	100.0050
SRILANKA	2005Q3	0.5071	0.0833	0.6055	10.7974	5.9406	15.0000	101.3098
SRILANKA	2005Q4	0.6775	0.0833	0.6055	8.2581	6.2417	15.0000	102.1172
SRILANKA	2006Q1	1.1030	0.0833	0.6055	7.5235	6.3250	15.0000	102.8006
SRILANKA	2006Q2	1.5600	0.0833	0.6055	8.8953	6.5600	15.0000	103.9759
SRILANKA	2006Q3	2.0500	0.0833	0.6055	9.8456	6.9848	15.0000	103.8197
SRILANKA	2006Q4	2.5736	0.0833	0.6055	11.7670	7.6683	15.0000	107.7056
SRILANKA	2007Q1	2.6241	0.0833	0.6055	13.6876	7.0808	15.0000	109.4056
SRILANKA	2007Q2	2.7054	0.0833	0.6055	12.8303	6.7675	15.0000	111.4150
SRILANKA	2007Q3	2.8137	0.0833	0.6055	14.8711	6.6777	15.0000	113.4708
SRILANKA	2007Q4	2.9434	0.0833	0.6055	17.1850	6.7968	15.0000	108.7194
SRILANKA	2008Q1	2.0207	0.0833	0.6055	20.0794	6.3807	15.0000	107.8164
SRILANKA	2008Q2	1.1029	0.0833	0.6055	23.5354	6.1252	15.0000	107.6900
SRILANKA	2008Q3	0.1798	0.0833	0.6055	22.5415	5.9890	15.0000	107.9071
SRILANKA	2008Q4	-0.7599	0.0833	0.6055	15.4570	5.9501	15.0000	113.1398
SRILANKA	2009Q1	-1.5035	0.0833	0.6055	7.3951	5.4257	15.0000	115.1458
SRILANKA	2009Q2	-2.2855	0.0833	0.6055	2.2702	4.9073	15.0000	114.9110
SRILANKA	2009Q3	-3.1158	0.0833	0.6055	1.0430	4.3106	15.0000	114.8091
SRILANKA	2009Q4	-4.0027	0.0833	0.6055	3.1260	3.5389	15.0000	114.3844
SRILANKA	2010Q1	-4.0584	0.0833	0.6055	7.0045	4.7631	15.0000	114.0449
SRILANKA	2010Q2	-4.1810	0.0833	0.6055	5.6864	5.8857	15.0000	113.5197
SRILANKA	2010Q3	-4.3720	0.0833	0.6055	4.9167	7.0888	15.0000	111.9277
SRILANKA	2010Q4	-4.6302	0.0833	0.6055	6.5241	8.5673	15.0000	110.9530
SRILANKA	2011Q1	-2.5218	0.0833	0.6055	6.8344	7.9164	15.0000	110.3929
SRILANKA	2011Q2	-0.4697	0.0833	0.6055	7.7014	7.6753	15.0000	109.6071
SRILANKA	2011Q3	1.5344	0.0833	0.6055	6.7078	7.8047	15.0000	110.1920
SRILANKA	2011Q4	3.4994	0.0833	0.6055	4.8027	8.3258	15.0000	113.9013
SRILANKA	2012Q1	3.0614	0.0833	0.6055	3.8914	7.4173	15.0000	128.1878
SRILANKA	2012Q2	2.5975	0.0833	0.6055	7.1915	6.8375	15.0000	133.3044
SRILANKA	2012Q3	2.1114	0.0833	0.6055	9.0761	6.4926	15.0000	129.7943
SRILANKA	2012Q4	1.6052	0.0833	0.6055	8.8047	6.3265	15.0000	127.1608
SRILANKA	2013Q1	1.0103	0.0833	0.6055	8.6809	6.4678	6.5000	126.8528
SRILANKA	2013Q2	0.3957	0.0834	0.6055	6.6111	6.7840	6.5000	129.9606
SRILANKA	2013Q3	-0.2394	0.0834	0.6055	6.0093	7.3264	6.5000	131.9809
SRILANKA	2013Q4	-0.8964	0.0837	0.6055	5.5129	8.1827	6.5000	130.7530
SRILANKA	2014Q1	-1.2285	0.0843	0.6055	2.9530	7.0902	6.5000	130.6949
SRILANKA	2014Q2	-1.5838	0.0860	0.6055	3.1258	6.2734	6.5000	130.3018
SRILANKA	2014Q3	-1.9620	0.0909	0.6055	2.9834	5.6000	6.5000	130.3707
SRILANKA	2014Q4	-2.3619	0.1050	0.6055	3.4492	4.9607	6.5000	131.0486
SRILANKA	2015Q1	-1.6299	0.1450	0.6055	6.2658	5.0510	6.5000	132.9000
SRILANKA	2015Q2	-0.9130	0.1591	0.6055	2.7845	5.0865	6.5000	133.7000

SRILANKA	2015Q3	-0.2061	0.1641	0.6055	1.8281	5.0728	6.5000	141.2335
SRILANKA	2015Q4	0.4962	0.1661	0.6055	3.9340	5.0077	6.5000	144.0623
SRILANKA	2016Q1	0.7108	0.1673	0.6055	1.0573	4.9877	6.0000	143.9000
SRILANKA	2016Q2	0.9320	0.1692	0.6055	5.2003	4.9026	6.0000	145.2500
SRILANKA	2016Q3	1.1649	0.1742	0.6055	4.8834	4.7385	6.0000	146.7229
SRILANKA	2016Q4	1.4138	0.1883	0.6055	4.3593	4.4688	6.0000	149.8000
SRILANKA	2017Q1	1.2406	0.2283	0.6055	7.4945	4.4108	7.0000	151.7354
SRILANKA	2017Q2	1.0898	0.2424	0.6055	7.0125	4.1941	7.0000	153.5100
SRILANKA	2017Q3	0.9635	0.2472	0.6055	7.3272	3.7835	7.0000	153.0983
SRILANKA	2017Q4	0.8631	0.2487	0.6055	7.8434	3.1126	7.0000	152.8548
SRILANKA	2018Q1	0.9874	0.2557	0.6055	6.4059	3.5016	2.2500	154.9510
SRILANKA	2018Q2	1.1385	0.2675	0.6055	5.5461	3.4251	2.2500	157.4556
SRILANKA	2018Q3	1.3159	0.2819	0.6055	4.6864	3.3485	2.2500	159.9602
SRILANKA	2018Q4	1.5183	0.2975	0.6055	3.8267	3.2720	2.2500	162.4649
SRILANKA	2019Q1	0.9588	0.3139	0.6055	3.5539	3.0178	2.0000	166.5349
SRILANKA	2019Q2	0.4196	0.3314	0.6055	3.2811	2.7636	2.0000	170.6049
SRILANKA	2019Q3	-0.1029	0.3500	0.6055	3.0084	2.5094	2.0000	174.6749
SRILANKA	2019Q4	-0.6126	0.3701	0.6055	2.7356	2.2552	2.0000	178.7449
SRILANKA	2020Q1	-0.9214	0.3917	0.6055	2.9080	0.7991	2.0000	180.4568
SRILANKA	2020Q2	-1.1867	0.4149	0.6055	3.0804	-0.6569	2.0000	182.1687
SRILANKA	2020Q3	-1.4610	0.4400	0.6055	3.2528	-2.1130	2.0000	183.8806
SRILANKA	2020Q4	-1.7660	0.4670	0.6055	3.4252	-3.5691	2.0000	185.5926
SRILANKA	2021Q1	0.1678	0.2666	0.6055	4.1000	4.0000	4.5000	198.3800
SRILANKA	2021Q2	0.1740	0.2696	0.6055	5.2000	16.3000	4.5000	199.0200
SRILANKA	2021Q3	0.1801	0.2727	0.6055	5.7000	-5.8000	5.0000	198.9700
SRILANKA	2021Q4	0.1862	0.2757	0.6055	12.1000	2.0000	5.0000	202.7200
TANZANIA	2000Q1	-0.0140	0.0000	0.5326	6.1541	7.2643	21.5000	800.5000
TANZANIA	2000Q2	-0.0934	0.0000	0.5326	5.7842	7.2651	21.5000	799.5400
TANZANIA	2000Q3	-0.1979	0.0000	0.5326	5.5579	7.2643	21.5000	799.1200
TANZANIA	2000Q4	-0.3265	0.0000	0.5326	5.5102	4.9338	21.5000	803.2600
TANZANIA	2001Q1	-0.2077	0.0000	0.5326	5.1480	7.2643	20.0000	860.0600
TANZANIA	2001Q2	-0.1101	0.0000	0.5326	5.1254	7.2651	20.0000	888.0300
TANZANIA	2001Q3	-0.0321	0.0000	0.5326	5.0022	7.2647	20.0000	890.0000
TANZANIA	2001Q4	0.0281	0.0000	0.5326	4.7949	5.9978	20.0000	916.3000
TANZANIA	2002Q1	0.0987	0.0000	0.5326	2.2471	4.4089	16.2500	981.2800
TANZANIA	2002Q2	0.1550	0.0000	0.5326	3.5937	5.9415	16.2500	946.9300
TANZANIA	2002Q3	0.1986	0.0000	0.5326	7.0632	8.6922	16.2500	969.4500
TANZANIA	2002Q4	0.2310	0.0000	0.5326	7.8728	9.4479	16.2500	976.3000
TANZANIA	2003Q1	0.2186	0.0000	0.5326	4.7295	5.7956	14.5000	1030.1500
TANZANIA	2003Q2	0.1978	0.0000	0.5326	5.3555	6.2904	14.5000	1047.3900
TANZANIA	2003Q3	0.1698	0.0000	0.5326	5.0046	7.1200	14.5000	1044.6500
TANZANIA	2003Q4	0.1354	0.0000	0.5326	5.5690	8.3284	14.5000	1063.6200
TANZANIA	2004Q1	0.0830	0.0000	0.5326	4.8700	7.7715	14.0000	1108.4100
TANZANIA	2004Q2	0.0261	0.0000	0.5326	3.7248	6.2375	14.0000	1107.3200
TANZANIA	2004Q3	-0.0344	0.0000	0.5326	4.5779	7.3445	14.0000	1060.4500
TANZANIA	2004Q4	-0.0980	0.0000	0.5326	5.3179	10.2453	14.0000	1042.9600
TANZANIA	2005Q1	-0.2133	0.0000	0.5326	4.0972	5.6715	15.2500	1104.2900
TANZANIA	2005Q2	-0.3300	0.0000	0.5326	5.3005	7.6675	15.2500	1126.3000
TANZANIA	2005Q3	-0.4469	0.0000	0.5326	5.4912	8.3478	15.2500	1136.2500
TANZANIA	2005Q4	-0.5629	0.0000	0.5326	4.7569	7.4445	15.2500	1165.5100
TANZANIA	2006Q1	-0.3650	0.0000	0.5872	7.4230	9.8224	15.7500	1223.8200
TANZANIA	2006Q2	-0.1628	0.0000	0.5872	8.7708	8.8701	15.7500	1253.0800
TANZANIA	2006Q3	0.0456	0.0000	0.5872	5.7171	5.6904	15.7500	1272.7000
TANZANIA	2006Q4	0.2625	0.0000	0.5872	6.0982	2.9244	15.7500	1261.6400
TANZANIA	2007Q1	0.4184	0.0000	0.5872	6.9324	4.4852	16.0000	1241.9600
TANZANIA	2007Q2	0.5869	0.0000	0.5872	5.4965	5.8627	16.0000	1264.9700
TANZANIA	2007Q3	0.7696	0.0000	0.5872	8.0518	7.1538	16.0000	1229.8900
TANZANIA	2007Q4	0.9678	0.0000	0.5872	6.6991	11.2141	16.0000	1132.0900
TANZANIA	2008Q1	0.8732	0.0000	0.5872	8.4840	7.8938	15.0000	1236.8200
TANZANIA	2008Q2	0.7953	0.0000	0.5872	8.9612	6.1494	15.0000	1180.9000
TANZANIA	2008Q3	0.7337	0.0000	0.5872	9.8041	7.7307	15.0000	1162.8900

TANZANIA	2008Q4	0.6877	0.0000	0.5872	11.8069	8.0245	15.0000	1280.3000
TANZANIA	2009Q1	0.3845	0.0000	0.5872	12.9730	5.5697	15.0000	1313.7000
TANZANIA	2009Q2	0.0940	0.0000	0.5872	11.4326	3.9493	15.0000	1314.2300
TANZANIA	2009Q3	-0.1858	0.0000	0.5872	11.7524	5.9154	15.0000	1312.7000
TANZANIA	2009Q4	-0.4569	0.0000	0.5872	9.7532	8.7476	15.0000	1326.8300
TANZANIA	2010Q1	-0.4475	0.0000	0.5872	6.0684	7.5525	14.5000	1330.2135
TANZANIA	2010Q2	-0.4329	0.0000	0.5872	7.2093	7.5576	14.5000	1379.3769
TANZANIA	2010Q3	-0.4143	0.0000	0.5872	5.8400	6.4491	14.5000	1483.8219
TANZANIA	2010Q4	-0.3927	0.0000	0.5872	4.9621	6.7803	14.5000	1453.5378
TANZANIA	2011Q1	-0.3225	0.0000	0.5872	7.0679	6.1286	15.0000	1490.7519
TANZANIA	2011Q2	-0.2506	0.0000	0.5872	9.2984	7.0628	15.0000	1572.0903
TANZANIA	2011Q3	-0.1774	0.0000	0.5872	13.6752	5.8529	15.0000	1631.2032
TANZANIA	2011Q4	-0.1031	0.0000	0.5872	17.3558	6.8311	15.0000	1566.6552
TANZANIA	2012Q1	-0.0709	0.0000	0.5872	17.6947	7.2962	15.5000	1575.0207
TANZANIA	2012Q2	-0.0376	0.0001	0.5872	16.6314	6.1695	15.5000	1568.9145
TANZANIA	2012Q3	-0.0032	0.0003	0.5872	13.7125	7.2385	15.5000	1569.2627
TANZANIA	2012Q4	0.0325	0.0010	0.5872	11.6440	7.0169	15.5000	1571.6210
TANZANIA	2013Q1	-0.1166	0.0028	0.5872	9.8492	7.4518	15.7500	1590.5170
TANZANIA	2013Q2	-0.2644	0.0081	0.5872	8.1156	6.6609	15.7500	1602.6566
TANZANIA	2013Q3	-0.4106	0.0230	0.5872	6.5571	6.3078	15.7500	1604.8755
TANZANIA	2013Q4	-0.5550	0.0656	0.5872	5.8635	7.2631	15.7500	1578.5664
TANZANIA	2014Q1	-0.4352	0.1869	0.5872	5.8545	7.2651	16.2500	1629.6025
TANZANIA	2014Q2	-0.3121	0.2325	0.5872	6.2004	7.2651	16.2500	1649.6727
TANZANIA	2014Q3	-0.1846	0.2572	0.5872	6.4122	7.2650	16.2500	1665.0661
TANZANIA	2014Q4	-0.0513	0.2905	0.5872	5.3425	6.9651	16.2500	1725.7840
TANZANIA	2015Q1	0.2251	0.3724	0.5872	4.0614	7.2650	16.0000	1788.0642
TANZANIA	2015Q2	0.5104	0.4011	0.5872	5.1661	7.2651	16.0000	2020.3485
TANZANIA	2015Q3	0.8058	0.4112	0.5872	6.1052	7.2650	16.0000	2149.0283
TANZANIA	2015Q4	1.1125	0.4148	0.5872	6.3847	6.9593	16.0000	2148.5200
TANZANIA	2016Q1	0.8676	0.4160	0.5872	5.6962	7.2650	16.0000	2179.6000
TANZANIA	2016Q2	0.6344	0.4164	0.5872	5.1317	7.2651	16.0000	2178.8600
TANZANIA	2016Q3	0.4122	0.4166	0.5872	4.6988	7.2650	16.0000	2175.3175
TANZANIA	2016Q4	0.1998	0.4166	0.5872	4.6736	6.9681	16.0000	2172.6209
TANZANIA	2017Q1	0.0804	0.4167	0.5872	5.5178	7.2650	17.5000	2223.9157
TANZANIA	2017Q2	-0.0318	0.4167	0.5872	5.8056	7.2651	17.5000	2230.1448
TANZANIA	2017Q3	-0.1385	0.4167	0.5872	5.0229	7.2650	17.5000	2237.7668
TANZANIA	2017Q4	-0.2412	0.4167	0.5872	4.3911	7.1042	17.5000	2230.0651
TANZANIA	2018Q1	-0.2813	0.4194	0.5872	8.2584	7.0712	15.1324	2095.6257
TANZANIA	2018Q2	-0.3200	0.4228	0.5872	8.2903	7.0724	15.1060	2114.7410
TANZANIA	2018Q3	-0.3584	0.4253	0.5872	8.3222	7.0736	15.0796	2133.8563
TANZANIA	2018Q4	-0.3975	0.4279	0.5872	8.3540	7.0747	15.0531	2152.9716
TANZANIA	2019Q1	-0.3675	0.4317	0.5872	8.3859	7.0759	15.0267	2172.0869
TANZANIA	2019Q2	-0.3393	0.4362	0.5872	8.4178	7.0770	15.0003	2191.2021
TANZANIA	2019Q3	-0.3128	0.4411	0.5872	8.4497	7.0782	14.9738	2210.3174
TANZANIA	2019Q4	-0.2882	0.4462	0.5872	8.4816	7.0794	14.9474	2229.4327
TANZANIA	2020Q1	-0.1068	0.4515	0.5872	8.5135	7.0805	14.9210	2248.5480
TANZANIA	2020Q2	0.0733	0.4568	0.5872	8.5453	7.0817	14.8945	2267.6633
TANZANIA	2020Q3	0.2528	0.4622	0.5872	8.5772	7.0828	14.8681	2286.7786
TANZANIA	2020Q4	0.4320	0.4676	0.5872	8.6091	7.0840	14.8417	2305.8939
TANZANIA	2021Q1	-0.0065	0.4155	0.5872	3.2000	5.0000	5.0000	2313.4000
TANZANIA	2021Q2	-0.0071	0.4221	0.5872	3.6000	3.8000	5.0000	2314.0000
TANZANIA	2021Q3	-0.0078	0.4287	0.5872	4.0000	5.5000	5.0000	2304.6000
TANZANIA	2021Q4	-0.0084	0.4354	0.5872	4.2000	5.5000	5.0000	2301.3000
