# Supplementary material for: Evaluation of central bank independence, macroprudential policy, and credit gap in developing countries
Source: PLoS One. 2023 May 16;18(5):e0285800. doi: 10.1371/journal.pone.0285800 (PMC10187913; doi:10.1371/journal.pone.0285800)
Supplement: Attachment — Submitted filename: Response to Reviewers.docx [file pone.0285800.s002.docx]

**Response to Reviewers**

**Title: Evaluation of Central Bank Independence, Macroprudential Policy, and Credit Gap in Developing Countries**

**No: PONE-D-23-00739**

| **Editor/**  **Reviewer** | **No** | **Reviewer’s Comment** | **Respond to Reviewer** |
| --- | --- | --- | --- |
| **Editor** | 1 | Please ensure that your manuscript meets PLOS ONE's style requirements, including those for file naming. | We have set the style of the manuscript by following the PLOS ONE’s style. |
|  | 2 | We note that you have stated that you will provide repository information for your data at acceptance. Should your manuscript be accepted for publication, we will hold it until you provide the relevant accession numbers or DOIs necessary to access your data. If you wish to make changes to your Data Availability statement, please describe these changes in your cover letter and we will update your Data Availability statement to reflect the information you provide. | We changed the Data Availability Statement to “The data available upon request” |
|  | 3 | Please review your reference list to ensure that it is complete and correct. If you have cited papers that have been retracted, please include the rationale for doing so in the manuscript text, or remove these references and replace them with relevant current references. Any changes to the reference list should be mentioned in the rebuttal letter that accompanies your revised manuscript. If you need to cite a retracted article, indicate the article’s retracted status in the References list and also include a citation and full reference for the retraction notice. | We have rewritten the citations and references. |
| **Editor** | 1 | The paper’s introduction section is not well organized therefore not well written at all. Too much reference has been cited in the introduction section which makes the reading uninteresting. There is hardly any linking conjunction, adverbs, propositions used in the whole manuscript which makes the paper incoherent. It seems that some references have been picked up from difference sources and jumbled up together. It’s also obsolete to use past tenses while stating a reference. There are no concluding sentences at the end of each paragraph of the introduction section. So, the introduction must be rewritten. | We have rewritten the introduction  [See Introduction section, pages 1-3] |
|  | 2. | The proof reading has not been done professionally. A lot of grammatical mistakes are also evident. | This manuscript has been checked by a professional proof reader. |
|  | 3. | The authors must clearly state why this study is important to conduct in the introduction section. The objective of the study is not mentioned in the introduction. | We have added the reasons why this study important and also the objectives of this study in the introduction section.  [See introduction section, page 2] |
|  | 4. | The contribution of the study is very poorly stated in two sentences and shockingly understated and  disorganized. | We have added contributions of this study in the introduction section.  [See introduction section, page 2-3] |
|  | 5. | It is utterly confusing the purpose of the literature mentioned in the introduction. Are they to mention the shortcomings of the existing literature or for mentioning the importance of conducting this study? | We have split the introduction and literature review.  [See introduction section, pages 1-3, and literature review pages 3-4] |
|  | 6. | The gap or shortcomings of the literature has not been mentioned anywhere in the literature review section. The authors continue to describe different studies but did not mention the gap in the literature. This section must be rewritten as well. It’s really hard to understand what authors mean by mentioning these studies. | We have added the research gap of the existing literature in the introduction section.  [See introduction section, page 2] |
|  | 7. | The result and discussion section are very much disorganized. The authors should clearly state which studies are in line with their findings and which contradicts. | We have rewritten the result and discussion of this study in the result section.  [See Empirical Result section, Comparation sub-sample subsection pages 11-12] |
|  | 8. | The paper does not mention any theoretical or anecdotal evidences which actually justify the selection of the variables in the context of used economies. | We have added theoretical and prior empirical studies for selection the variables in this study in the Methodology section, Data subsection  [See Methodology section, Data subsection, pages 6-7] |
|  | 9. | The robustness of the results has not been checked. The robustness can be checked by changing model  idealizations or changing background conditions. | The robustness check has a different model with the initial model.  [See Robustness Test subsection, pages 12-13] |
|  |  |  |  |
| **Reviewer 1** | 1. | A stronger motivation and economic intuition for the topic should be provided in introduction section. Scarcity does not mean necessity. You need to prove your paper is valuable enough and distinguish from others | We have added motivations and contributions of this study in the introduction section.  [See introduction section, page 2-3] |
|  | 2. | Separate the literature review from introduction section. | We have separated the literature review from introduction section  [See introduction section, pages 1-3, and literature review pages 3-4] |
|  | 3. | Based upon your empirical results, propose concrete policy recommendations and provide future directions for researchers. | We have included the recommendations and future research in the conclusion section.  [See conclusion section, page 14] |
