# Supplementary material for: Evaluation of central bank independence, macroprudential policy, and credit gap in developing countries
Source: PLoS One. 2023 May 16;18(5):e0285800. doi: 10.1371/journal.pone.0285800 (PMC10187913; doi:10.1371/journal.pone.0285800)
Supplement: Attachment — Submitted filename: Response to Reviewers.docx [file pone.0285800.s003.docx]

**Response to Reviewers**

**Title: Evaluation of Central Bank Independence, Macroprudential Policy, and Credit Gap in Developing Countries**

**No: PONE-D-23-00739R1**

| **Editor/**  **Reviewer** | **No** | **Reviewer’s Comment** | **Respond to Reviewer** |
| --- | --- | --- | --- |
| **Reviewer 2** | 1 | Title is adequate | We do not change the title |
|  | 2 | It would be helpful to provide more context about the credit gap and its significance in developing countries, as well as a more detailed explanation of the panel threshold nonlinear model used in the study. Additionally, the abstract could benefit from more specific and concise language to improve clarity and understanding for readers. | We have added more explanation regarding the credit gap in developing countries.  [See Introduction section, pages 2-3]  We have added more explanation regarding the panel non-linear model.  [See Introduction section, page 3] |
|  | 3 | Introduction looks adequate but following can be improved: |  |
|  | a | Providing a more detailed context for the global monetary crisis of 2007-2008 and its impact on the role of central banks in ensuring financial stability. | We have added more explanation for the global monetary crisis of 2007-2008 and its impact on the role of central banks in ensuring financial stability  [See Introduction section, page 1] |
|  | b | Citing more recent and relevant empirical studies on the relationship between CBI, MAPP, and financial stability, in addition to those mentioned in the introduction. | We have added more recent and relevant empirical studies on the relationship between CBI, MAPP, and financial stability  [See Introduction section, page 3] |
|  | c | Providing a more specific and concise definition of the credit gap and its relevance to financial stability, to help readers better understand the study's main criteria for measuring stability. | We have added more specific and concise definition of the credit gap and its relevance to financial stability  [See introduction section, page 3] |
|  | d | Explaining the rationale for using a panel threshold nonlinear model and the dummy variable technique, as these are relatively technical and may require more explanation for readers who are not familiar with them. | We have added the explanation the rationale for using a panel threshold nonlinear model and the dummy variable technique [See introduction section, page 3] |
|  | 4. | Literature needs to be detailed. Very few studies. | We have added literature review.  [See literature review pages 4-6] |
|  | 5. | No research questions or hypothesis ? provide them | We have added the research questions or hypothesis in the introduction section.  [See Methodologysection, page 6] |
|  | 6. | Overall, the econometric methodology is appropriate for this research, but there may be some gaps in addressing potential heterogeneity across nations and providing a more detailed explanation of the non-standard poolability test. Why not fixed model? Generalized Method of Moments (GMM) | The fixed effect estimation does not solve heterogeneity explanatory variabels coefficients. We apply GMM estimation for robustness test.  [See Robustness subsection, pages 14-15] |
|  | 7. | The data collection and sources are adequate for this study. But provide more information on the selection of the 20 developing nations included in the analysis. Were they selected randomly or based on specific criteria? What was the rationale behind the selection? | We have added more information on the selection of the 20 developing nations included in the analysis Methodology section, Data subsection  [See Methodology section, Data subsection, page 8] |
|  | 8. | Tests are adequate but no proper discuss instead of repeating the same information from earlier sections, the conclusion should provide a brief and straightforward overview of the study's key results and implications. The language could also be made more accessible to readers who may not have a background in econometrics. Additionally, the conclusion could suggest future research directions, potential limitations of the study, and areas where policymakers could focus their efforts based on the study's findings | We have rewritten the conclusion section  [See Conclusion Section, pages 15-16] |
|  | 9. | Practical implications are missing. | We have added the practical implications.  [See Conclusion Section, page 16] |
